# A Bioinformatics Model of Human Diseases on the Basis of Differentially Expressed Genes (of Domestic Versus Wild Animals) That Are Orthologs of Human Genes Associated with Reproductive-Potential Changes

**DOI:** 10.3390/ijms22052346

**Published:** 2021-02-26

**Authors:** Gennady Vasiliev, Irina Chadaeva, Dmitry Rasskazov, Petr Ponomarenko, Ekaterina Sharypova, Irina Drachkova, Anton Bogomolov, Ludmila Savinkova, Mikhail Ponomarenko, Nikolay Kolchanov, Alexander Osadchuk, Dmitry Oshchepkov, Ludmila Osadchuk

**Affiliations:** 1 Novosibirsk State University, 630090 Novosibirsk, Russia; genn@bionet.nsc.ru; 2 Institute of Cytology and Genetics, Siberian Branch of Russian Academy of Sciences, 630090 Novosibirsk, Russia; ichadaeva@bionet.nsc.ru (I.C.); rassk@bionet.nsc.ru (D.R.); pon.petr@gmail.com (P.P.); sharypova@bionet.nsc.ru (E.S.); drachkova@bionet.nsc.ru (I.D.); mantis_anton@bionet.nsc.ru (A.B.); lksav@bionet.nsc.ru (L.S.); kol@bionet.nsc.ru (N.K.); osadchuk@bionet.nsc.ru (A.O.); diman@bionet.nsc.ru (D.O.); losadch@bionet.nsc.ru (L.O.)

**Keywords:** reproductive potential, animal model of human disease, domestication, in vivo verification, RNA-seq, differentially expressed gene

## Abstract

Earlier, after our bioinformatic analysis of single-nucleotide polymorphisms of TATA-binding protein-binding sites within gene promoters on the human Y chromosome, we suggested that human reproductive potential diminishes during self-domestication. Here, we implemented bioinformatics models of human diseases using animal in vivo genome-wide RNA-Seq data to compare the effect of co-directed changes in the expression of orthologous genes on human reproductive potential and during the divergence of domestic and wild animals from their nearest common ancestor (NCA). For example, serotonin receptor 3A (*HTR3A*) deficiency contributes to sudden death in pregnancy, consistently with *Htr3a* underexpression in guinea pigs (*Cavia porcellus)* during their divergence from their NCA with cavy (*C. aperea*). Overall, 25 and three differentially expressed genes (hereinafter, DEGs) in domestic animals versus 11 and 17 DEGs in wild animals show the direction consistent with human orthologous gene-markers of reduced and increased reproductive potential. This indicates a reliable association between DEGs in domestic animals and human orthologous genes reducing reproductive potential (Pearson’s χ^2^ test *p* < 0.001, Fisher’s exact test *p* < 0.05, binomial distribution *p* < 0.0001), whereas DEGs in wild animals uniformly match human orthologous genes decreasing and increasing human reproductive potential (*p* > 0.1; binomial distribution), thus enforcing the norm (wild type).

## 1. Introduction

As Theodore Dobzhansky noted, “man is genetically specialized to be unspecialized” [1]. People have settled down, indeed, in all landscapes and all climates of all continents [2,3]. To this end, humans did not adapt anthropometrically to the natural environment like animals do but rather adapted it (as an artificial anthropogenic environment) to their own lives, including the domestication of other organisms to satisfy their own needs and even whims [4,5]. Of note, many behavioral and anatomical differences between domestic and wild animals can correspond to human disease symptoms. For example, the diminished weakened heart of domestic ducks versus the large powerful heart of wild ducks reflects settledness versus spring-and-autumn migrations [6], and this relation may correspond to myocardial infarction as the most common cause of death in humans, as rated by the World Health Organization (WHO) [7]. Currently, translational medicine researchers [8] already use domestic animals, including transgenic ones [9], in preclinical trials of drugs intended for human treatments. Thus, bioinformatics models of human diseases on the basis of differentially expressed genes (DEGs) of domestic versus wild animals (from animal genome-wide transcriptomes determined in vivo) are relevant and useful in the post-genomic era of life sciences.

According to Ernst Mayer’s concept [10], the cornerstone for the existence of a biological species is a set of populations related to one another due to reproduction, as follows: each previous generation (parents) must leave behind the next generation (offspring). In 1987, the WHO classified human reproduction biomedical risk factors (in decreasing order): female infertility (38%), seeming infertility in biologically parental couples (27%), male infertility (20%), and everything else (15%) [11]; this ranking has remained true to date [12]. That is why the Centers for Disease Control and Prevention (USA) are uniformly systematizing and coordinating world-wide studies on female infertility, whereas male infertility studies are still scattered across diverse local databases that collect anecdotal information only on those individual patients who are anxious about their own reproductive disorders [12].

At the same time, phylogenetic reconstruction of the coevolution of chromosomes X and Y of a wide range of species (opossums, cattle, rats, mice, marmosets, rhesus monkeys, chimpanzees, and humans) on the basis of data from whole-genome high-throughput sequencing [13] indicates that in addition to reproductive function, these chromosomes determine gender dimorphism in both predisposition to diseases and in organism viability as a whole. With this in mind, at either a populational or individual scale, reproductive potential is the most common conventional indicator of the probability to survive, produce offspring, and bring it to a reproductive state under the best conditions [14,15]. As for human biomedicine, in the narrowest sense, reproductive potential means only a set of infertility risk factors (e.g., [16]), whereas its broadest interpretation includes a wide variety of physiological, mental, behavioral, social, anthropometric, and genetic indicators contributing to humankind’s reproduction as a whole [17].

According to Konrad Lorenz’s ideas [18], if there is a need to protect one’s own life, offspring, home, territory, or access to limited resources, then reproductive behavior can also be accompanied by aggressiveness. In social animals (including humans), the aggressiveness of an individual determines his/her socio-hierarchical rank, which affects the quality of his/her life and his/her lifespan [19]. Moreover, in many human diseases, aggressiveness can be a risk factor (e.g., [20]), a symptom (e.g., [21]), or a complication of treatment (e.g., [22]). On a whole-genome scale, many animals are already subjected to aggressiveness studies, including the trout [23], chickens [24], foxes [25,26], dogs [27,28], pigs [29,30], rats [29,31], mice [32], rabbits, and guinea pigs [29]; however, this research still does not take into account the reference human genome [33], human variome [34], or the classification of diseases and health problems ICD-11 [35].

Nevertheless, now among the most challenging problems is how to restore many critically endangered animal species within the unclear natural limitations of population restoration by captive breeding [36]. One possible step towards solving this urgent problem could be a bioinformatics model of human diseases on the basis of differentially expressed genes (of domestic versus wild animals) that are orthologs of human genes associated with reproductive potential changes, in order to determine how the anthropogenic environment, compared to natural wildlife, can alter the animal gene expression on the genome-wide scale and thus affect reproductive potential.

In our previous works, we performed genome-wide analyses of single-nucleotide polymorphisms (SNPs) of TATA-binding protein (TBP)-binding sites in the promoters of genes associated with human reproductive health [37,38,39,40,41]. As a result of these bioinformatic estimates, we proposed that human reproductive potential diminishes during self-domestication [42]. This is why, here, we, for the first time, proposed to utilize domestic versus wild animal transcriptomes determined in vivo as an animal bioinformatics model of human diseases, and, then, within this model framework, verified our above-mentioned hypothesis in silico on experimental datasets of the DEGs of domestic versus wild animals. Finally, we discussed the results obtained with respect to how it could be possible to domesticate new economically valuable animals without reducing their reproductive potential as well as why the return from anthropogenic habitat conditions to natural ones (in the wild) is actually contributing to the restoration of endangered animal species.

## 2. Results and Discussion

**Figure 1:**
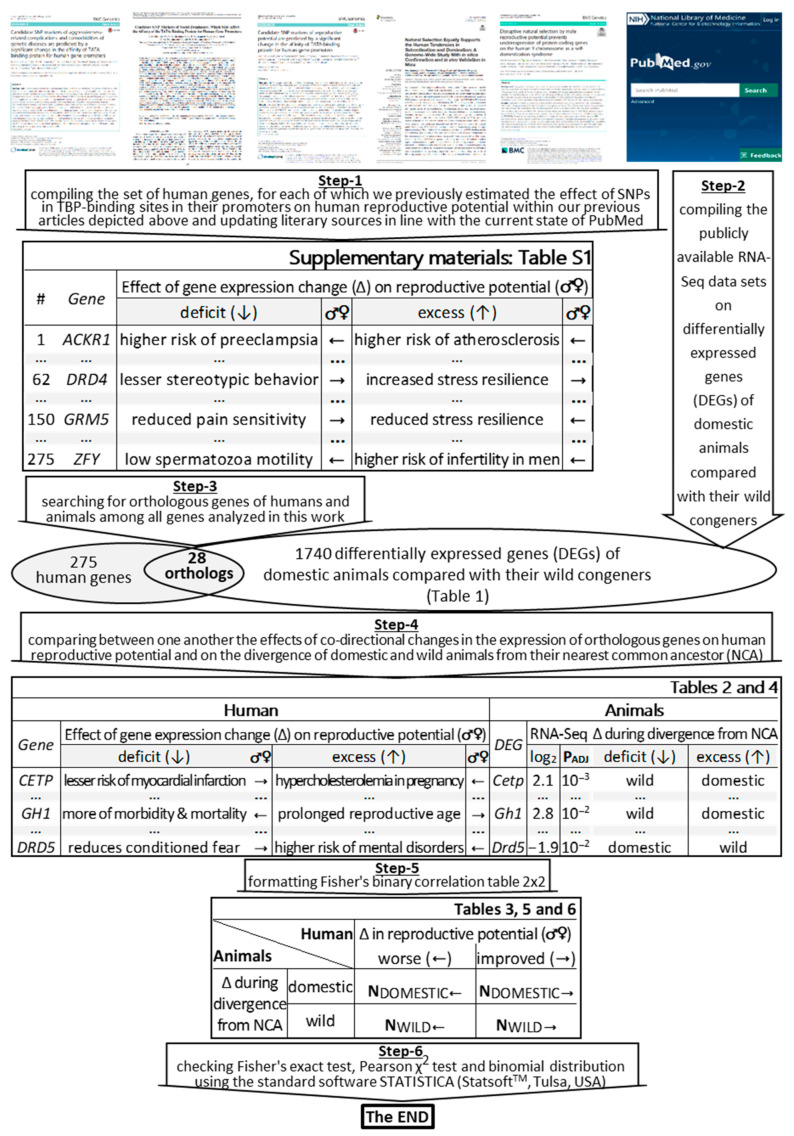
An algorithmic flow chart of the bioinformatics model of human diseases on the basis of differentially expressed genes (of domestic versus wild animals) that are orthologs of human genes associated with reproductive potential changes. Legend: Δ, gene expression change: deficit (↓), excess (↑); ♂♀, reproductive potential change: worse (←), improved (→); NCA, nearest common ancestor; *P_ADJ_*, significance (Fisher’s Z-test with those corrections on multiple comparisons, which are available within the RNA-Seq data, as published by their authors [26,28,29]).

In this work, we verified our whole-genome sequence-based a priori hypothesis (derived in silico [41]) that human reproductive potential can diminish during self-domestication [42], as depicted in Figure 1 and described in the Section 3.

### 2.1. The Bioinformatics Model Developed

As the bioinformatics model development, first of all, we compiled the set of all the 275 human genes whose effects on human reproductive potential have been a priori estimated by means of SNPs within their 70 bp proximal promoters in our previous articles on this matter [37,38,39,40,41] and, next, updated literary sources in line with the current state of PubMed [43], as shown in Figure 1 (Step-1) and presented in Table S1 (hereinafter, see Supplementary Materials). Then, by means of PubMed [43], we compiled independent publicly available experimental in vivo RNA-Seq data sets on domestic versus wild animals [26,28,29], as depicted in Figure 1 (Step-2) and presented in Table 1. For the RNA-Seq data analysis, here, we, for the first time, resumed the use of one of the eldest [44] commonly accepted (e.g., see [45]) and widely used (e.g., see [46]) concepts of “divergence from the nearest common ancestor (NCA)” within the framework of the microevolution theory in relation to domesticated and wild animals, whose differentially expressed genes (DEGs) were compared with orthologous human genes under this study, as depicted in Figure 1. After that, we found 28 pairs of orthologous genes in humans and animals among all genes analyzed in this work, as depicted by a Venn diagram in Figure 1 (Step-3). The largest part (13) of the 28 DEGs (46%) characterizes the guinea pig (*Cavia porcellus*) in comparison with the cavy (*C. aperea*) [29]. Additionally, the remaining 15 DEGs (54%) characterize domestic rabbits (*Oryctolagus cuniculus*) [29], dogs (*Canis familiaris*) [28,29], and foxes (*Vulpes vulpes*) [26] versus their wild congeners (including the wolf, *C. lupus*). Next, we compared the effects of co-directional changes in the expression of orthologous genes on human reproductive potential and on the divergence of domestic and wild animals from their NCA, as shown in Figure 1 (Step-4). On this basis, we formatted Fisher’s binary correlation tables 2 × 2, depicted in Figure 1 (Step-5). Finally, we used the standard software STATISTICA (Statsoft^TM^, Tulsa, OK, USA), the pathway “Statistics” → “Nonparametric” → “table 2 × 2” of which was provided by the Fisher’s exact test, Pearson χ2 test and binomial distribution test to verify our findings (Figure 1: Step-6).

Each of these DEGs is discussed in the following.

### 2.2. DEGs of the Guinea Pig Versus Cavy and How Their Human Orthologous Genes Change Reproductive Potential 

Human gene *CETP* encodes cholesteryl ester transfer protein, whose deficit lowers the risk of myocardial infarction [47] and improves human reproductive potential consistently with cavy gene *Cetp* downregulation during this wild animal’s microevolution from the above NCA [29]. Conversely, *CETP* overexpression is a risk factor of hypercholesterolemia during late pregnancy [48] and reduces human reproductive potential; the direction of expression change is consistent with the guinea pig gene *Cetp* overexpression in microevolution [29], as shown in Table 2. 

Human genes *CHRNA3* and *CHRNA6* correspond to cholinergic receptor nicotinic subunits α3 and α6, deficiencies of which elevate human reproductive potential owing to improvements in finding opposite sex congeners [49] and in maternal behavior [51], respectively, in agreement with expression changes of cavy orthologous genes during microevolution [29]. Conversely, CHRNA3 overexpression and CHRNA6 overexpression worsen human reproductive potential through worse effects of nicotine compounds on primordial oocytes [50] and via a higher risk of social defeats [52], in agreement with the direction of expression change of the guinea pig orthologous genes in microevolution [29] (Table 2). 

Human gene *DRD5* codes for dopamine receptor D5, whose deficit reduces conditioned fear [53] and improves reproductive potential (Table 2) co-directedly with guinea pig *Drd5* underexpression during microevolution [29]. As an alternative, DRD5 upregulation raises risks of mental disorders [54] and worsens human reproductive potential, co-directedly with the cavy gene Cyp17a1 decrease in expression during microevolution [29]. 

Human gene *FLT4* produces feline McDonough sarcoma-like tyrosine kinase 4, a deficit of which suppresses melanoma metastasis [55] and enhances human reproductive potential consistently with the cavy gene *Flt4* expression change as compared with the NCA of the guinea pig and cavy [29]. On the other hand, FLT4 excess exacerbates post-traumatic inflammatory neovascularization in humans [56] and worsens human reproductive potential co-directedly with the guinea pig orthologous gene during microevolution [29] (Table 2).

Human genes *GFRA3* and *GFRA4* correspond to GDNF family receptors α3 and α4, low expression of which worsens reproductive potential via accelerated neurodegeneration [57] and premature adolescent bone formation [59], respectively, in agreement with the direction of expression change of the corresponding guinea pig and cavy orthologous genes during their microevolution (Table 2). Conversely, both GFRA3 and GFRA4 excesses can increase human reproductive potential because of improvements in neural regeneration [58] and neuronal survival [60], respectively.

Human gene *HTR3A* encodes serotonin receptor 3A, whose deficit elevates the risk of sudden cardiac death in pregnant women [61] consistently with the guinea pig orthologous gene in microevolution [29] (Table 2), whereas HTR3A overexpression improves mood and behavior [62].

Human gene *IL1B* encodes interleukin 1β, the decreased expression of which prevents bone deformations in infections [63] and improves reproductive potential in agreement with cavy Il1b downregulation in microevolution [29]. Contrariwise, IL1B overexpression enhances circadian pain hypersensitivity [64], worsening reproductive potential in line with Il1b excess in guinea pig microevolution [29], as shown in Table 2.

Human gene *NR5A1* encodes steroidogenic factor 1, whose underexpression and overexpression correspond to impaired [65] and improved [66] gonadal development, respectively, as well as to expression changes of the guinea pig and cavy orthologous genes during their divergence from their NCA [29] (Table 2).

Human gene *PDGFRL* (platelet-derived growth factor receptor–like protein) underexpression reduces tumor mutation burden [67], thus raising reproductive potential consistently with the cavy ortholog expression alteration during microevolution [29], whereas a PDGFRL excess leads to hypertensive behavior and myocardial hypertrophy [68] (Table 2).

Human gene *PDYN* encodes prodynorphin, underexpression of which reduces reproductive potential through obesity-related subfertility [69] in agreement with the direction of expression change of the cavy ortholog downregulation in the course of microevolution [29], but a PDYN excess prevents conditioned fear behavior [70] (Table 2).

Human gene *SLC6A4* produces serotonin transporter 1, whose deficiency improves small-intestine function [71], thus elevating reproductive potential consistently with the cavy orthologous gene expression change during microevolution [29], whereas SLC6A4 overexpression worsens depression, anxiety, and inertia [72] (Table 2). 

Table 3 summarizes the results of the comparison of the above orthologous genes of humans and guinea pigs, namely, 11 and two of these guinea pig DEGs were found to correspond to human gene-markers of worsened and improved reproductive potential, and the same is true for three and ten DEGs in the cavy. This means that the DEGs in guinea pigs significantly correspond to the human orthologous genes reducing human reproductive potential according to three independent tests, namely, Pearson’s χ^2^ test (*p* < 0.01), Fisher’s exact test (*p* < 0.05), and binomial distribution (*p* < 0.05), in contrast to cavy DEGs, which almost equally fit human orthologous genes worsening and improving this human trait (*p* > 0.05, binomial distribution), thus enforcing the wild-type norm. 

To verify that this phenomenon is not species-specific among animals, we considered the DEGs of domestic rabbits, dogs, and foxes as compared with their wild congeners in relation to human gene-markers of reproductive potential changes, as revealed in this work (Table 4).

### 2.3. DEGs of Domestic Versus Wild Animals and How the Human Orthologous Genes Alter Reproductive Potential

Human gene *F7* encodes coagulation factor VII, and its underexpression correlates with life-threatening bleeding [73], thus reducing reproductive potential in agreement with the direction of expression change of domestic rabbit F7 relative to the NCA of domestic and wild rabbits [29], whereas recombinant F7 is a drug saving life and fertility during intractable obstetric bleeding in women [74] (Table 4).

Human gene *PDGFRA* encodes platelet-derived growth factor receptor α, both a deficit and excess of which worsen reproductive potential through skeletal defects in newborns [75] and predisposition to infertility after infections [76] (Table 4).

Human gene *GABARAPL2* produces GABA type A receptor-associated protein-like 2, whose downregulation retards wound healing [77] and reduces reproductive potential consistently with the dog orthologous gene’s expression change during both dog and wolf divergence from their NCA [28]. Conversely, a GABARAPL2 excess improves tooth injury healing [78], as shown in Table 4.

Human gene *GH1* encodes growth hormone 1, whose underexpression raises the risks of morbidity and mortality from cardiovascular diseases [79] and reduces reproductive potential, in agreement with the wolf *Gh1* underexpression in microevolution [28] (Table 4). On the contrary, recombinant GH1 is used as a drug to prolong the reproductive age in women [80].

Human gene *HBB* codes for hemoglobin subunit β, whose deficit (thalassemia) worsens women’s reproductive health [81], in agreement with the direction of expression change of the dog orthologous gene—deficit during microevolution [28] (Table 4). Conversely, in traditional Chinese medicine, the Jian-Pi-Yi-Sheng decoction (JPYS) is employed to raise the HBB level for treating anemia in chronic kidney diseases [82]

Human gene *NRP2* produces neuropilin 2, the downregulation of which improves survival after radiochemotherapy [83], thereby raising reproductive potential consistently with wolf orthologous gene downregulation in microevolution [28] (Table 4). Conversely, an NRP2 excess causes post-traumatic vascular neointimal hyperplasia [84] (Table 4).

Human gene *TAC3* codes for tachykinin precursor 3, whose deficit and excess correspond to high and low risks of socially induced subfertility [85] as well as to expression changes of the *Tac3* gene during the divergence of dogs and wolfs from their NCA [28] (Table 4).

Human gene *TGFB3* encodes transforming growth factor β3, whose deficit and excess worsen reproductive potential in men [86] and women [87], respectively (Table 4).

Human gene *ESR2* corresponds to estrogen receptor 2; both deficiency and overabundance in adolescents worsen spermatogenesis in adult males [88] (Table 4).

Human gene *GRIN3A* codes for glutamate ionotropic receptor NMDA type subunit 3A whose underexpression prevents cocaine addiction [89], thus improving reproductive potential consistently with the wild fox Grin3a deficiency in microevolution [26] (Table 4). Oppositely, a GRIN3A excess increases the risk of inattentive behavior [90] and, therefore, reduces reproductive potential that fits a Grin3a excess during the tame fox microevolution [26], as shown in Table 4.

Human gene *HTR3B* encodes serotonin receptor 3B, the downregulation of which reduces anger-resolutive behavior [91], thereby reducing reproductive potential in agreement with the expression alteration of the tame fox orthologous gene during microevolution [26] (Table 4), whereas *HTR3B* overexpression decreases the risk of pulmonary embolism [92].

Human gene *IL6ST* codes for interleukin 6 signal transducer; both deficit and excess worsen reproductive potential through increased risk of mortality during sepsis [93] and sensitivity to fatigue [94], respectively, as shown in Table 4.

Human gene *IL9R* encrypts interleukin 9 receptor; both downregulation and overabundance impair reproductive potential via impaired trophoblast implantation in preeclampsia [95] and increased risk of life-threatening anaphylaxis [96].

Human gene NPY codes for neuropeptide Y, whose deficit [97] and excess [98] cause subfertility, as presented in Table 4.

Human gene *TGFB2* produces transforming growth factor β2; its downregulation and upregulation reduce reproductive potential through increased risk of perinatal mortality [99] and impaired wound healing [100], respectively (Table 4).

Table 5 sums up the findings of the comparative analysis of the above orthologous genes from humans, rabbits, dogs, wolfs, and foxes. For example, 14 and one of these domestic animal DEGs were found to correspond to human gene-markers of reduced and elevated reproductive potential, and the same is true for eight and seven DEGs in the wild animals. Accordingly, once again, we observed that the DEGs in domestic animals reliably correspond to their human orthologous genes impairing human reproductive potential, according to Pearson’s χ^2^ test (*p* < 0.05), Fisher’s exact test (*p* < 0.05), and binomial distribution (*p* < 0.001). On the other hand, DEGs of wild animals correlate equally to human orthologous genes, which weaken and enhance human reproductive potential (*p* > 0.5, binomial distribution), which corresponds to the wild-type norm, as indicated in Table 5.

### 2.4. DEGs in Domestic Animals Reliably Correspond to Their Human Orthologs Reducing Reproductive Potential

Generalizing Table 3 and Table 5, we found that 25 and three DEGs in domestic animals as compared with 11 and 17 DEGs in wild animals correspond to the human orthologous gene-markers of reduced and increased reproductive potential (Table 6). Therefore, DEGs in domestic animals reliably correspond to their human orthologous genes that diminish reproductive potential, judging by Pearson’s χ^2^ test (*p* < 0.001), Fisher’s exact test (*p* < 0.05), and binomial distribution (*p* < 0.0001). By contrast, DEGs of wild animals are equally fitting human orthologous genes decreasing and increasing reproductive potential (in terms of binomial distribution, *p* > 0.1), thereby possibly enforcing the wild-type norm.

This is why it is important to underscore that our bioinformatic hypothesis examined here was confirmed in vivo in animal models of human diseases by means of independent experimental RNA-Seq data from domestic and wild animals and was formulated in accordance with the concept of human reproductive potential as the most comprehensive indicator of chances for successful survival, production of offspring, and bringing these offspring to reproductive age [10,14,15]. As for the actual realization of these chances in practice, we found literary evidence both in favor and against one-to-one correspondence between domestication and reduced reproductive potential in animals. First of all, the observed correspondence between the dog-versus-wolf DEGs and clinically proven markers of reduced human reproductive potential (Table 4) is consistent with the correspondence between some deviations characteristic of autism spectrum disorders in humans and both physiological and behavioral differences of dogs from wolves [42]. Moreover, tame foxes (Table 4) as compared to wild ones have a worse female endocrine system [101], reduced sexual activity in first-year males [102,103], accelerated extinction of testicle hormonal function [104], impaired reproductive seasonality [105], and reduced gonad mass together with heterochrony of pituitary–spermatic complex development in the embryo [106]. Additionally, compared to aggressive male rats, tame ones show delayed puberty [107]. Additionally, the results of this work are supported by the finding that a return of Saiga antelope (*Saiga tatarica*) [108] and Amur tigers (*Panthera tigris altaica*) [109] from anthropogenic habitat conditions to natural ones (in the wild) has already successfully helped to restore these endangered species.

Curiously, as a counterargument to these five observations, there is a good example of domestic pigs, which outperform wild boars on both sperm quality and spermatogenesis [110]. This phenomenon may be due to artificial selection aimed at improving their fertility for greater meat production. This means that if during the domestication of new economically valuable animal species (e.g., the musk deer *Moschus berezovskii* [111]), artificial selection for improved target traits is supplemented with selection for higher fertility, then this approach may compensate the risks of the domestication process, e.g., reduced reproductive potential, as reported by many authors elsewhere [42,101,102,103,104,105,106,107].

## 3. Materials and Methods

### 3.1. Human Genes under Study

Here, we studied 275 human genes, which are described in Table S1 (see Supplementary Materials) according to the results of our in silico analysis of the effect of the SNPs (located in proximal promoters) on human reproductive health [37,38,39,40,41]; the literature supporting these data was updated according to the current state of the PubMed database [43], as depicted in Figure 1 (Step-1). 

### 3.2. DEGs of Domestic Animals Compared to Their Wild Congeners

In this work, we used publicly available independent experimental RNA-Seq datasets on transcriptomes of domestic versus wild animals [26,28,29]. Although, here, we compare the DEG of each animal with one human orthologous gene, we nevertheless limited our analysis to only those DEGs that were statistically significant according to Fisher’s Z-test, with those corrections on multiple comparisons (P_ADJ_ < 0.05), which are publicly available within the RNA-Seq data studied here, as published by their authors [26,28,29]. As a result, a total of 1740 DEGs were analyzed, namely, within the frontal cortex, there were 883 DEGs of guinea pigs versus cavies, 30 DEGs of pigs versus boars, 20 DEGs of tame versus aggressive rats, 17 DEGs of domesticated versus wild rabbits, and 13 DEGs of dogs versus wolves [29], as well as 450 DEGs in the blood of dogs versus wolves [28] and 327 DEGs in the pituitary gland of tame versus aggressive foxes [26] (Table 1 and Figure 1: Step-2).

The only novelty of this work is that for the RNA-Seq data analysis, we, for the first time, resumed the use of one of the most ancient [44] conventional (e.g., see [45]) and actual (e.g., see [46]) microevolutional concepts of “divergence from the nearest common ancestor (NCA)” in the case of domestic and wild animals, whose differentially expressed genes (DEGs) were compared with orthologous human genes under this study (Figure 1: Step-3). This allows us to compare the effects of co-directional changes in the expression of orthologous genes on human reproductive potential and on the divergence of domestic and wild animals from their NCA, as shown in Table 2 and Table 4, as well as in Figure 1 (Step-4).

### 3.3. Statistical Analysis

Using Table 2 and Table 4, we made the standard statistical tables 2 × 2 (Figure 1: Step-5), which are the input data for the standard software STATISTICA (Statsoft^TM^, Tulsa, OK, USA), where its pathway “Statistics” → “Nonparametric” → “table 2 × 2” led to the Fisher’s exact test, Pearson χ2 test, and binomial distribution test to verify the significance of our results (Figure 1: Step-6).

### 3.4. The Knowledge Base on Domestic Animals’ DEGs Whose Human Orthologous Genes Can Change Reproductive Potential

We Excel-compatibly formatted all the identified associations between DEGs of domestic versus wild animals and the effects of the human orthologous genes on reproductive potential as a textual flat file and finally converted it into the PetDEGsDB knowledge base format in Web environment MariaDB 10.2.12 (MariaDB Corp AB, Espoo, Finland). The PetDEGsDB knowledge base made by this work is publically available on the web page www.sysbio.ru/domestic-wild (accessed on 26 February 2021).

## 4. Conclusions

In this work, we, for the first time, proposed to utilize differences between domestic and wild animal transcriptomes as a bioinformatics model of human diseases. Within this model framework, we successfully confirmed (Table 2 and Table 4) our a priori hypothesis (derived in silico) that human reproductive potential can diminish during self-domestication [42]. This hypothesis was formulated on the basis of our genome-wide sequence-based analysis of SNPs within 70 bp proximal promoters of human Y-linked genes [41]. As we presented in Table 2, Table 3, Table 4, Table 5 and Table 6, DEGs of domestic versus wild animals of the same species indicate genetic differences among all intergroup differences that are statistically significant in comparison with the variability within the species in question. (Some variability is necessary for the existence of a species.) This property makes these DEGs a promising tool for microevolution research (Table 2 and Table 4). 

Finally, in this work, we fully analyzed the genes of human neurotransmitter and neurotrophinergic systems as well as genes on the human Y chromosome. Therefore, in the future, an expansion of the list of such genes to, for example, the human endocrine and immune systems may increase the completeness of the reproductive potential analysis. With this in mind, in the future, a similar extended bioinformatic analysis of RNA-Seq data on the multifactorial human diseases will be interesting, because it will estimate the effects of predisposition and resistance to such diseases on human reproductive potential.

## Figures and Tables

**Table 1:** The investigated genome-wide RNA-Seq transcriptomes of domestic animals with their wild congeners, publicly available in database PubMed.

#	Domestic Animals	Wild Animals	Tissue	Number of DEGs	[Ref]
1	tame foxes (*Vulpes vulpes*) 6 males:	aggressive foxes (*V. vulpes*) 6 males:	pituitary	327	[26]
2	dogs (*Canis familiaris*): 1 female and 1 male	wolves (*C. lupus*): 2 females and 1 male	blood	450	[28]
3	dogs (*C. familiaris*): 2 females and 3 males	wolves (*C. lupus*): 2 females and 1 male	frontal cortex	13	[29]
4	pigs (*Sus scrofa*): 5 females	boars (*S. scrofa*): 5 females	frontal cortex	30	[29]
5	guinea pigs (*Cavia porcellus*): 3 females and 3 males	cavy (*C. aperea*): 3 females and 3 males	frontal cortex	883	[29]
6	domesticated rabbits (*Oryctolagus cuniculus domesticus*): 3 females and 3 males	wild rabbits (*Oryctolagus cuniculus*): 3 females and 3 males	frontal cortex	17	[29]
7	tame rats (*Rattus norvegicus*): 3 females and 3 males	aggressive rats (*R. norvegicus*): 3 females and 3 males	frontal cortex	20	[29]
Σ	6 domesticated animal species: 17 females and 19 males	6 wild animal species: 18 females and 17 males	3 tissues	1740	3 Refs

Note: DEG, differentially expressed gene; Ref, reference.

**Table 2:** Comparing the effects of changes in the expression of orthologous genes on human reproductive potential and on the divergence of the guinea pig and cavy from their nearest common ancestor (NCA) [29].

Humans					Animals				
Gene	Effect of Gene Expression Change (Δ) on Reproductive Potential (♂♀)				*DEG*	*RNA-Seq*		Δ during Diver-Gence from NCA	
	Deficit (↓)	♂♀	Excess (↑)	♂♀		*log*	*P_ADJJ_*	Deficit (↓)	Excess (↑)
*CETP*	lesser risk of myo-cardial infarction [47]	**→**	hypercholesterolemia in pregnancy [48]	**←**	*Cetp*	2.1	10^−3^	wild	domestic
*CHRNA3*	improved finding oppo-site sex congeners [49]	**→**	greater nicotine effects on oocytes [50]	**←**	*Chrna3*	0.9	0.05	wild	domestic
*CHRNA6*	improved maternal behavior [51]	**→**	higher risk of social defeats [52]	**←**	*Chrna6*	0.9	0.05	wild	domestic
*DRD5*	reduces conditioned fear [53]	**→**	higher risk of mental disorders [54]	**←**	*Drd5*	–1.9	10^−2^	domestic	wild
*FLT4*	suppressed melanoma metastasis [55]	**→**	worse post-injury neo- vascularization [56]	**←**	*Flt4*	0.8	0.05	wild	domestic
*GFRA3*	accelerated neuro-degeneration [57]	**←**	improved neural regeneration [58]	**→**	*Gfra3*	–1.0	0.05	domestic	wild
*GFRA4*	premature adolescent bone formation [59]	**←**	improved neuronal survival [60]	**→**	*Gfra4*	1.5	0.05	wild	domestic
*HTR3A*	higher risk of death during pregnancy [61]	**←**	improved mood and behavior [62]	**→**	*Htr3a*	–2.9	10^−14^	domestic	wild
*IL1B*	less bone deformation in infections [63]	**→**	circadian pain hypersensitivity [64]	**←**	*Il1b*	2.3	10^−2^	wild	domestic
*NR5A1*	higher risk of gonadal dysgenesis in men [65]	**←**	improved gonadal development [66]	**→**	*Nr5a1*	–2.2	0.05	domestic	wild
*PDGFRL*	reduced tumor mutation burden [67]	**→**	myocardial hypertrophy [68]	**←**	*Pdgfrl*	1.3	10^−8^	wild	domestic
*PDYN*	obesity-related subfertility [69]	**←**	prevented conditioned fear behavior [70]	**→**	*Pdyn*	0.9	10^−2^	wild	domestic
*SLC6A4*	improved small-intestinal function [71]	**→**	worse depression, anxiety, inertia [72]	**←**	*Slc6a4*	2.9	10^−2^	wild	domestic

Note: see the caption of Figure 1; log, the ratio of a domestic-animal gene expression level to that in wild animals (log_2_ units); *P_ADJ_*, significance (Fisher’s Z-test with those corrections on multiple comparisons, which are available within the RNA-Seq data, as published by their authors [26,28,29]).

**Table 3:** Correlations between the effects of the co-directed changes in the expression of orthologous genes on human reproductive potential and on the divergence of the guinea pig and cavy from their NCA.

	Humans	Change in Reproductive Potential (♂♀)		Binomial Distribution	χ^2^-Test		Fisher’s Test	
Animals		Worse (←)	Improved (→)		χ^2^	*p*	Value	*p*
change during divergence from NCA	domestic	11	2	0.05	9.91	10^−2^	0.003	0.05
	wild	3	10	0.06				

Note: see Figure 1 caption.

**Table 4:** Comparing the effects of changes in the expression of orthologous genes on human reproductive potential and on the divergence of domestic and wild animals from their nearest common ancestor (NCA).

Humans					Animals				
Gene	Effect of Gene Expression Change (Δ) on Reproductive Potential (♂♀)				*DEG*	*RNA-Seq*		Δ during Diver-Gence from NCA	
	Deficit (↓)	♂♀	Excess (↑)	♂♀		*log*	*P_ADJJ_*	Deficit (↓)	Excess (↑)
					domesticated *versus* wild rabbits [29]				
*F7*	higher risk of bleeding [73]	**←**	recombinant F7 treats obstetric bleeding [74]	**→**	*F7*	–2.7	0.05	domestic	wild
					dog versus wolf (frontal cortex) [29]				
*PDGFRA*	skeletal defects in newborns [75]	**←**	higher risk of infertility [76]	**←**	*Pdgfra*	1.5	10^−3^	wild	domestic
					dog *versus* wolf (blood) [28]				
*GABARAPL2*	impaired wound healing [77]	**←**	improved tooth injury healing [78]	**→**	*Gabarapl2*	–2.0	10^−2^	domestic	wild
*GH1*	higher risk of mortality [79]	**←**	prolonged reproductive age in women [80]	**→**	*Gh1*	2.8	10^−2^	wild	domestic
*HBB*	worse reproductive health in women [81]	**←**	relieved anemia in kidney diseases [82]	**→**	*Hbbl*	–5.9	10^−8^	domestic	wild
*NRP2*	better survival after radiochemotherapy [83]	**→**	vascular neointimal hyperplasia [84]	**←**	*Nrp2*	1.8	0.05	wild	domestic
*TAC3*	higher risk of subfertility [85]	**←**	lesser socially induced subfertility [85]	**→**	*Tac3*	–4.7	10^−8^	domestic	wild
*TGFB3*	lowers semen quality and infertility [86]	**←**	higher risk of female infertility [87]	**←**	*Tgfb3*	3.3	0.05	wild	domestic
					tame *versus* aggressive foxes [26]				
*ESR2*	impaired spermatogenesis [88]	**←**	impaired spermatogenesis [88]	**←**	*Esr2*	–0.3	0.05	domestic	wild
*GRIN3A*	prevents cocaine addiction [89]	**→**	higher risk of inatten-tive behavior [90]	**←**	*Grin3a*	0.5	10^−2^	wild	domestic
*HTR3B*	reduced anger-reso-lutive behavior [91]	**←**	lesser risk of pulmo-nary embolism [92]	**→**	*Htr3b*	–0.5	0.05	domestic	wild
*IL6ST*	higher risk of mortality during sepsis [93]	**←**	increased sensitivity to fatigue [94]	**←**	*Il6st*	0.3	0.05	wild	domestic
*IL9R*	impaired trophoblast implantation [95]	**←**	higher risk of anaphylaxis [96]	**←**	*Il9r*	0.4	0.05	wild	domestic
*NPY*	higher risk of infertility [97]	**←**	higher risk of obesity and subfertility [98]	**←**	*Npy*	0.4	10^−2^	wild	domestic
*TGFB2*	higher risk of perinatal mortality [99]	**←**	impaired wound healing [100]	**←**	*Tgfb2*	0.5	10^−2^	wild	domestic

Note: see the caption of Figure 1 and the footnote of Table 2.

**Table 5:** Correlations between the effects of the co-directed changes in the expression of orthologous genes on human reproductive potential and on the divergence of domestic and wild animals from their NCA (without guinea pig and cavy, the correlations for which are outlined in Table 2 and Table 3).

	Humans	Change in Reproductive Potential (♂♀)		Binomial Distribution	χ^2^-Test		Fisher’s Test	
Animals		Worse (←)	Improved (→)		χ^2^	*p*	Value	*p*
change during divergence from NCA	domestic	14	1	10^−3^	6.14	0.05	0.04	0.05
	wild	8	7	0.5				

Note: see Figure 1 caption.

**Table 6:** Correlations between the effects of the co-directed changes in the expression of orthologous genes on human reproductive potential and on the divergence of domestic and wild animals from their NCA.

	Humans	Change in Reproductive Potential (♂♀)		Binomial Distribution	χ^2^-Test		Fisher’s Test	
Animals		Worse (←)	Improved (→)		χ^2^	*p*	Value	*p*
change during divergence from NCA	domestic	25	3	10^−4^	15.2	10^−3^	10^−4^	0.05
	wild	11	17	0.1				

Note: see Figure 1 caption.

## Supplementary Materials

The following are available online at https://www.mdpi.com/1422-0067/22/5/2346/s1.

## Abbreviations

DEG	differentially expressed gene
NCA	nearest common ancestor
SNP	single-nucleotide polymorphism
WHO	World Health Organization
