# Supplementary material for: A Bioinformatics Model of Human Diseases on the Basis of Differentially Expressed Genes (of Domestic Versus Wild Animals) That Are Orthologs of Human Genes Associated with Reproductive-Potential Changes
Source: Int J Mol Sci. 2021 Feb 26;22(5):2346. doi: 10.3390/ijms22052346 (PMC7956675; doi:10.3390/ijms22052346)
Supplement: Supplementary file 1 [file ijms-22-02346-s001.pdf]

International Journal of Molecular Sciences

MDPI

*SUPPLEMENTARY MATERIALS*

# A Bioinformatics Model of Human Diseases on the basis of Differentially Expressed Genes (of Domestic versus Wild Animals) That Are Orthologs of Human Genes Associated with Reproductive-Potential Changes

**Vasiliev[1,2] G, Chadaeva[2] I, Rasskazov[2] D, Ponomarenko[2] P, Sharypova[2] E, Drachkova[2] I, Bogomolov[2] A, Savinkova[2] L, Ponomarenko[2,*] M, Kolchanov[2] N, Osadchuk[2] A, Oshchepkov[2] D, Osadchuk[2] L**

1 Novosibirsk State University, Novosibirsk 630090, Russia;
2 Institute of Cytology and Genetics, Siberian Branch of Russian Academy of Sciences, Novosibirsk 630090, Russia;
* Correspondence: pon@bionet.nsc.ru. Tel.: +7 (383) 363-4963 ext. 1311 (M.P.)

## Supplementary data on effects of the human gene underexpression or overexpression under this study on the reproductive potential

**Table S1.** Effects of underexpression or overexpression of the human genes under this study on the reproductive potential according to our estimates [1-5].

| # | Human Gene | Deficit (↓) | | | Excess (↑) | | |
|---|---|---|---|---|---|---|---|
| | | *N*SNP | Effect on reproductive potential [Reference] | ♂♀ | *N*SNP | Effect on reproductive potential [Reference] | ♂♀ |
| 1 | *ACKR1* | 1 [3] | increased risks of preeclampsia as one of the most challenging problems of modern obstetrics [8] | ← | | increased risk of atherosclerosis and other coronary artery disease [9] | ← |
| 2 | *ADCYAP1* | 1 [4] | within a model of human diseases using Adcyap1-knockout mice, decreased fertility [10] | ← | 3 [4] | in a model of human health using transgenic mice overexpressing Adcyap1 within only pancreatic β-cells, ameliorated diabetes [11] | → |
| 3 | *ADCYAP1R1* | 2 [4] | within a model of human diseases using Adcyap1r1-knockout female mice, decreased fertility [12] | ← | 4 [4] | increased risks of increased chronic post-traumatic nociceptive pain-related behavior [13] | ← |
| 4 | *ADORA1* | 5 [4] | within a model of human fertility using Adora1-knockout male mice, delayed sperm maturation and, ultimately, fewer offspring [14] | ← | 5 [4] | in a human health model using norm rather than Adora1-knockout mice, Adora1-agonist improves post-ejaculation sperm ripening [15] | → |
| 5 | *ADORA2A* | 3 [4] | within a model of human diseases using adult male mice, predisposition to fearfulness, helplessness, and fatigue [16] | ← | 10 [4] | within a model of human diseases using mice, improved survival in post-traumatic endotoxemia and sepsis [17] | → |
| 6 | *ADORA2B* | | in models of men fertility using mice, increased risks of insufficiencies in maturation and storage of spermatozoa within epididymis [18] | ← | 1 [4] | within a model of human diseases using mice, increased risks of voluntary physically-inactive behavior [19] | ← |
| 7 | *ADORA3* | 2 [4] | within a model of human diseases using Adora3-knockout mice, platelet deficit with bleeding without blood coagulation in trauma [20] | ← | 9 [4] | within models of human diseases using mice, chronic pain insensitivity [21] | → |
| 8 | *ADRA1A* | 2 [4] | within a model of human diseases using ADRA1A-knockout mice, twice reduced pregnancy rate [22] | ← | 9 [4] | within a model of human diseases using mice carrying constitutively more bioactive ADRA1A-mutant, antidepressant-like behavior [23] | → |
| 9 | *ADRA1D* | | within a model of human diseases using triple ADRA1A-, ADRA1B-, and ADRA1D-knockout mice, drastic reduced pregnancy rate [22] | ← | 1 [4] | reduced risks of muscle atrophy after trauma and diseases, as well as during ageing [24] | → |

***Note***: **N**SNP, as the number of candidate SNP markers that significantly decrease or increase the affinity of the TATA-binding protein (TBP) for the promoters of the considered gene according to estimates cited as [Ref] and, thereby, decrease (↓) or increase (↑) the expression of this gene, as has been repeatedly proven by many independent experiments (e.g., [6], for a review, see [7]); ♂♀, as effects on human reproductive potential: deterioration (←) or improvement (→). ***Genes***: *ACKR1*, atypical chemokine receptor 1 (synonym: Duffy blood group); *ADCYAP1*, adenylate cyclase activating polypeptide 1; *ADCYAP1R1*, pituitary adenylate cyclase-activating polypeptide type 1 receptor; *ADORA1*, *ADORA2A*, *ADORA2B*, and *ADORA3*, adenosine receptors A1, A2a, A2b, and A3, respectively; *ADRA1A and ADRA1D*,, adrenoceptors α1A. and α1D, respectively,

**Table S1.** Cont.

| # | Human Gene | Deficit (↓) | | | Excess (↑) | | |
|---|---|---|---|---|---|---|---|
| | | $N_{SNP}$ | Effect on reproductive potential [Reference] | ♂♀ | $N_{SNP}$ | Effect on reproductive potential [Reference] | ♂♀ |
| 10 | *ADRA2C* | | in human disease models using Adra2c-knockout mice, higher startle responses, lesser startle reflex inhibition and attack-respond delay [25] | ← | 2 [4] | in human disease models using Adra2c-excessive mice, lesser startle responses, more startle reflex inhibition and attack-respond delay [25] | → |
| 11 | *ADRB2* | 2 [4] | within models of human fertility using aging female rats, propranolol blocks Adrb2 and thus successfully prolongs their reproductive age [26] | → | | within models of human fertility using aging female rats, the age-induced increase of Adrb2 level reduces their fertility indicators [26] | ← |
| 12 | *ADRB3* | | in a model of human behavior using Adrb3-knockout mice, resistance to antidepressant-treatment against post-stress worsen physical state [27] | ← | 1 [4] | within models of human diseases using rats and mice, synthetic Adrb3-agonist SR58611A causes antidepressant-like behavior [28] | → |
| 13 | *AKAP17A* | 6 [5] | predisposition to accelerated aging in men [29] | ← | 13 [5] | increased risk of azoospermia through testicular degeneration [30] | ← |
| 14 | *AMELY* | 1 [5] | increased risk of suicide in men [31] | ← | | exogenous recombinant amelogenin is a wound healing drug [32] | → |
| 15 | *APOA1* | 1 [3] | increased risk of mental disorders according to low score of Montreal Cognitive Assessment (MoCA) [33] | ← | | unexplained infertility in women [34] | ← |
| 16 | *AR* | | increased risk of early mortality through metabolic diseases because of disturbed gut microbiota [35] | ← | 3 [3] | androgen-induced premature aging in adult men [36] | ← |
| 17 | *ARTN* | 3 [4] | within a model of human embryogenesis using mouse embryos, impaired neurotrophic support of tissue innervation [37] | ← | 9 [4] | in models of human behavior using Artn-knockdown mice, exogenous Artn has antidepressant-like effect at 30 min after administration [38] | → |
| 18 | *ASMT* | 3 [5] | increased risks of inflammatory airway diseases such as asthma because of melatonin deficiency [39] | ← | 10 [5] | melatonin excess protects sperm from oxidative DNA damage [40] | → |
| 19 | *ASMTL* | 5 [5] | increased risk of prostate cancer [41] | ← | 13 [5] | increased risk of autism spectrum disorders [42] | ← |
| 20 | *AVPR1A* | | within a model of human pregnancy using Avpr1a-deficient female mice, fewer pups, labor initiation delay, stronger postpartum bleeding [43] | ← | 2 [4] | within a human cohort-based comparative clinical study, increased risks of depression-like behavior [44] | ← |
| 21 | *AVPR2* | 1 [4] | within a model of human preeclampsia using co-infusion of vasopressin with Avpr2-antagonist into mice, prevented fetal growth restriction [45] | → | 1 [4] | in human preeclampsia models using gravid mice norm injected by only vasopressin without Avpr2-antagonist, reduced fetal mass [45] | ← |
| 22 | *BDNF* | 4 [4] | within a female human cohort-based comparative clinical study, increased risks of both moderate and severe depressive behavior [46] | ← | 10 [4] | within retrospective meta-analysis of BDNF-related publications in-between January 2018 and February 2019, better woman fertility [47] | → |
| 23 | *CC2D1A* | | in human disease models using mice with either knockout or conditional knockdown of Cc2d1a, perinatal lethality or cognitive deficit, respectively [48] | ← | 6 [2] | increased risks of both anxious and depressive behavior [49] | ← |
| 24 | *CC2D1B* | 4 [2] | within a model of human diseases using Cc2d1b-knockout mice, increased risks of cognitive deficit [50] | ← | 8 [2] | within human behavior models using rats, increased level of fear-induced aggressive response [51] | → |
| 25 | *CD99* | 3 [5] | anti-CD99 drugs retard atherogenesis that reduce risks of stroke and myocardial infarction as two most often causes of human death [52] | → | 20 [5] | increased mortality from septic shock in men [53] | ← |
| 26 | *CDNF* | 1 [4] | within a model of human diseases using CDNF-knockout mice, increased risks of degenerated enteric neurons [54] | ← | 4 [4] | within human disease models using exogenous CDNF injected into normal mice brain, prevented dopaminergic neuron degeneration [55] | → |

***Genes***: *ADRA2C, ADRB2,* and *ADRB3,* adrenoceptors α2C, β2, and β3, respectively; *AKAP17A,* A-kinase anchoring protein 17A; *AMELY,* amelogenin Y-linked; *APOA1,* apolipoprotein A1; *AR,* androgen receptor; *ARTN,* artemin; *ASMT,* acetylserotonin O-methyltransferase; *ASMTL,* N-acetylserotonin O-methyltransferase-like protein; *AVPR1A* and *AVPR2,* arginine vasopressin receptors 1A and 2, respectively; *BDNF,* brain derived neurotrophic factor; *CC2D1A* and *CC2D1B,* Freud-1 and Freud-2, respectively; *CD99,* CD99 molecule (synonym: Xg blood group); *CDNF,* cerebral dopamine neurotrophic factor.

**Table S1.** Cont.

| # | Human Gene | Deficit (↓) | | | Excess (↑) | | |
|---|---|---|---|---|---|---|---|
| | | $N_{SNP}$ | Effect on reproductive potential [Reference] | ♂♀ | $N_{SNP}$ | Effect on reproductive potential [Reference] | ♂♀ |
| 27 | *CDY2A* | 1 [5] | male maturation arrest [56] | ← | | partly repaired fertility in men, who have AZFc-deletion containing the *CDY1* gene-paralog necessary to finalize spermatogenesis [57] | → |
| 28 | *CETP* | 1 [3] | retarded atherogenesis that reduce risks of both stroke and myocardial infarction as two most often causes of human death [58] | → | 3 [3] | increased risks of hypercholesterolemia in late pregnancy [59] | ← |
| 29 | *CHRM1* | 1 [4] | within a model of human diseases using Chrm1-knockout mice, larger pancreatic intraepithelial neoplasia area and shorter overall survival [60] | ← | 1 [4] | increased risks for chronic fatigue syndrome [61] | ← |
| 30 | *CHRM2* | 1 [4] | within a model of human diseases using rats inoculated by Japanese encephalitis virus, worse spatio temporal learning and memory [62] | ← | 2 [4] | within a human cohort-based clinical study, improved susceptibility to antidepressant mood stabilizers in depressive disorders [63] | ← |
| 31 | *CHRM3* | | within a model of human post-injury cure using human neurons, CHRM3-antagonist improved oligodendrocyte repair in brain and spinal cord [64] | → | 1 [4] | within a human cancer cell clinical study ex vivo, increased risks of benign prostatic hyperplasia as a prostate disease precursor [65] | ← |
| 32 | *CHRM4* | 1 [4] | within a model of human diseases using rats, increased risks of both acute and chronic arthritis [66] | ← | 2 [4] | within clinical study using cell lines of prostate cancer vs norm, increased risks of neuroendocrine prostate cancer [67] | ← |
| 33 | *CHRM5* | 1 [4] | based on a clinical case of patient carrying *de novo* interstitial 5,3 Mb-deletion of chromosome 15 containing *CHRM5*, raised risks of mental retardation [68] | ← | 4 [4] | in human behavior models using mice, antidepressant imipramine upregulated CHRM5 to treat for chronic stress complications [69] | → |
| 34 | *CHRNA1* | 1 [4] | within a model of human neuromuscular hyperactivity complications using zebrafish, motor axonal extension and muscular degeneration [70] | ← | 2 [4] | in human amyotrophic lateral sclerosis models using mice: skeletal gastrocnemius, quadriceps and soleus muscles denervation [71] | ← |
| 35 | *CHRNA2* | | in human pregnancy models using Chrna2-null mice, prevented negative effects of maternal nicotine exposure on learning and memory in pups [72] | → | 7 [4] | within clinical study of pedigree segregating sleep-related epilepsy: higher risk of seizures, fear sensation, and nocturnal wanderings [73] | ← |
| 34 | *CHRNA3* | 2 [4] | within a model of human diseases using songbirds, improved finding an opposite sex tribesman [74] | → | | enhanced adverse effects of nicotine compounds on primordial oocytes [75] | ← |
| 35 | *CHRNA4* | | within a model of human behavior using Chrna4-knockout mice, increased risks of anxiety [76] | ← | 13 [4] | within a model of human diseases using mice, increased risks of congestive heart failure [77] | ← |
| 36 | *CHRNA5* | 4 [4] | within a model of human cord brain injury using Chrna5-knockdown rats, relieved mechanical pain [78] | → | 5 [4] | within a model of human cord brain injury using rats, hypersensitivity to mechanical pain [78] | ← |
| 37 | *CHRNA6* | 2 [4] | enhanced maternal behavior [79] | → | | within a model of human diseases using mice, increased risks of both neuropsychiatric disorders and social defeats [80] | ← |
| 38 | *CHRNA7* | 2 [4] | within a model of human behavior using mice administered by Chrna7-antagonist, improved antidepressant-like behavior [81] | → | 2 [4] | according to the retrospective exhaustive review, behavioral and cognitive benefits compared to norm due to excessive Ca(2+) ions [82] | → |
| 39 | *CHRNA9* | 1 [4] | in human pain models using Chrna9-knockout mice, both the development and maintenance of chronic mechanical hyperalgesia were reduced [83] | → | 1 [4] | based on the bioinformatics meta-analysis of microarray datasets from ArrayExpress database, increased risks of gliomas and glioblastoma [84] | ← |
| 40 | *CHRNA10* | 1 [4] | in human pain sensitivity models using adult male rats administered by Chrna10-antagonist, chronic neuropathic hyperalgesia reduced [85] | → | 1 [4] | in human viability models using human and rat blood cells stimulated with Chrna10-agonists, innate immune response estimate reduced [86] | ← |
| 41 | *CHRNB1* | 3 [4] | based on the family history of calves carrying "Chrna1-loss of function" gene: increased risks of neuromuscular disorders and fetal lethality [87] | ← | 3 [4] | within a model of human motor activity using mice, reduced muscle size with increased efficiency of muscle functioning [88] | → |

***Genes***: *CDY2A*, chromodomain Y-linked 2A; *CETP*, cholesteryl ester transfer protein; *CHRM1, CHRM2, CHRM3, CHRM4*, and *CHRM5*, cholinergic muscarinic receptors 1, 2, 3, 4, and 5, respectively; *CHRNA1, CHRNA2, CHRNA3, CHRNA4, CHRNA5, CHRNA6, CHRNA7, CHRNA9, CHRNA10, and CHRNB1*, cholinergic nicotinic receptor subunits α1, α2, α3, α4, α5, α6, α7, α9, α10, and β1, respectively.

**Table S1.** Cont.

| # | Human Gene | Deficit (↓) | | | Excess (↑) | | |
|---|---|---|---|---|---|---|---|
| | | $N_{SNP}$ | Effect on reproductive potential [Reference] | ♂♀ | $N_{SNP}$ | Effect on reproductive potential [Reference] | ♂♀ |
| 43 | *CHRNB3* | 1 [4] | within a model of human depressive behavior using rats, increased risks of stress-induced both anhedonia and mood despair [89] | ← | | in human health models using Chrnb3-null mice, Chrnb3-excess may raises voluntary nicotine intake as a leading cause of preventable human death [90] | ← |
| 44 | *CHRNB4* | 3 [4] | within a model of human depressive behavior using rats, increased risks of stress-induced both anhedonia and mood despair [89] | ← | 2 [4] | in human behavior models using transgenic mice carrying CHRNB4 on bacterial artificial chromosome: reduced work memory & impulsiveness [91] | ← |
| 45 | *CHRNE* | | based on a case of a patient carrying genomic mutation reducing CHRNE level: predisposition to limb weakness and ophthalmoplegia [92] | ← | 3 [4] | within a cohort-based clinical study of human myasthenia gravis: predisposition to authoimmune-related muscle weakness [93] | ← |
| 46 | *CHRNG* | 1 [4] | within a model of human embryogenesis using Chrng-knockout mice, increased risks of fetal lethality [94] | ← | | within a cohort-based clinical study, increased risks of rhabdomyosarcoma (embryonal rhabdomyosarcoma, 60% of cases) [95] | ← |
| 47 | *CNR1* | 1 [4] | within a model of human behavior using Cnr1-knockout adult male mice, increased risks of anxiety and depression in men [96] | ← | 6 [4] | in human behavior models using mice carrying artificial "gain-of-function" mutation of Cnr1 gene: adolescent behavior in adulthood [97] | → |
| 48 | *CNTF* | 1 [4] | within a model of human behavior using Cntf-knockout mice, increased risks of both anxiety and depression [98] | ← | | in human post-injury vision repair models using CNTF-overexpressing neural stem cells injected into the mice eye injured, neuroprotection [99] | ← |
| 49 | *COMT* | 6 [4] | within a model of human behavior using the Comt-knockout female mice, increased risks of stress-induced both anxiety and depression [100] | ← | 17 [4] | in human depression models using the Flinders Sensitive Line (FSL) of rats, increased risks of anxiety and depressive-like behavior [101] | ← |
| 50 | *CRLF2* | 2 [5] | weakened symptoms of acute respiratory tract infections in children and the elderly [102] | → | 4 [5] | increased risks of B-cell acute lymphoblastic leukemia in children [103] | ← |
| 51 | *CSF2RA* | 9 [5] | Csf2ra-knockout mice (as models of human diseases using laboratory animals) have respiratory failure [104] | ← | 4 [5] | lentiviral vectors carrying the mouse Csf2ra gene have passed preclinical trials in mice for the treatment of respiratory failure [104] | → |
| 52 | *CXCR4* | 1 [4] | within a model of human behavior using CXCR4-null mice, increased risks of both motor coordination and balance impaired [105] | ← | 3 [4] | in human behavior models using bee venom injection into rats, development and maintenance of persistent pain hypersensitivity [106] | ← |
| 53 | *CYP2A6* | 2 [3] | reduced damage from passive smoking for non-smoking pregnant women [107] | → | | *Smilax china L.* root extract increases CYP2A6 levels to detoxify nicotine from tobacco smoke condensate in the lungs [108] | → |
| 54 | *CYP2B6* | 2 [3] | increased risk of hepatocellular carcinoma [109] | ← | | improved detoxification of toxins in the liver [110] | → |
| 55 | *CYP17A1* | 1 [3] | increased risk of reduced fertility [111] | ← | 1 [3] | Malaysian propolis increases CYP17A1 level in the testes as a drug to overcome subfertility in diabetics [112] | → |
| 56 | *DHFR* | 3 [3] | DHFR-inhibitors are anti-mycobacterial drugs for tuberculosis [113] | → | 2 [3] | increased risks of ectopic pregnancy, metastatic choriocarcinoma, and gestational trophoblastic disease [114] | ← |
| 57 | *DHRSX* | 6 [5] | within a human disease model using HeLa cells, DHRSX knockdown reduces autophagy level as response to starvation [115] | ← | 3 [5] | increased risk of stroke in men in middle age (i.e., reproductive age) [116] | ← |
| 58 | *DNMT1* | 2 [3] | small doses of decitabine (a nucleoside analog) deplete the epigenetic DNMT1 regulator as a treatment for myeloid tumor [117] | → | 7 [3] | within model of human disease using mice, increased risks of epigenetic disorders of fetal brain development under stress [118] | ← |
| 59 | *DRD1* | | within a model of human embryogenesis using osteoblast-specific Ddr1-knockdown mice, reduced body weight and body length in newborns [119] | ← | 1 [4] | within a model of human behavior using adult male rats inoculated with a lentiviral vector carrying DRD1 gene, more sexual activity [120] | → |

***Genes***: *CHRNB3, CHRNB4, CHRNE,* and *CHRNG*, cholinergic nicotinic receptor subunits β3, β4, ε, and γ, respectively; *CNR1,* cannabinoid receptor 1; *CNTF,* ciliary neurotrophic factor; *COMT,* catechol-O-methyltransferase; *CRLF2,* cytokine receptor like factor 2; *CSF2RA,* colony stimulating factor 2 receptor subunit α; *CXCR4,* Fusin; *CYP17A1,* steroid 17α-monooxygenase; *CYP2A6,* xenobiotic monooxygenase; *CYP2B6,* 1,4-cineole 2-exo-monooxygenase; *DHFR,* dihydrofolate reductase; *DHRSX,* *d*ehydrogenase/reductase X-linked; *DNMT1,* DNA methyltransferase 1; *DRD1,* dopamine receptors D1.

**Table S1.** Cont.

| # | Human Gene | Deficit (↓) | | | Excess (↑) | | |
|---|---|---|---|---|---|---|---|
| | | $N_{SNP}$ | Effect on reproductive potential [Reference] | ♂♀ | $N_{SNP}$ | Effect on reproductive potential [Reference] | ♂♀ |
| *60* | *DRD2* | 3 [4] | within a model of human behavior using Drd2-knockout mice, decreased risks of depressive-like behavior [121] | → | 10 [4] | within a model of human behavior using rats, increased risks of the chronic stress-induced depression-like behavior [122] | ← |
| *61* | *DRD3* | 1 [4] | in human behavior models using Drd3-null mice, increased risks of depression in those adult who were exposed to stress in childhood [123] | ← | 3 [4] | within a model of human behavior using mice, increased motor activity and behavior motivation [124] | → |
| *62* | *DRD4* | 1 [4] | within a model of human behavior using Drd4-knockout mice: increased locomotor activity and reduced stereotypic behavior [125] | → | 3 [4] | within a human cohort-based clinical psychiatric study, increased stress resilience [126] | → |
| *63* | *DRD5* | | in models of human behavior using rats, Drd5-antagonist (rather Drd5-agonist) blocks freezing behavior in conditioned fear [127] | → | 10 [4] | in models of human diseases using rats, prenatal nicotine exposure rises Drd5 level in the striatum as a cause of newborn mental disorders [128] | ← |
| *64* | *ESR2* | 2 [3] | within model of human disease using rats, ESR2-deficiency in adolescents reduces sperm quality in adults [129] | ← | | within model of human disease using rats, ESR2-excess in adolescents reduces sperm quality in adults [129] | ← |
| *65* | *F2* | | $\alpha$1-antitrypsin inhibits F2 and, thereby, prevents thromboembolism and micro- and macrothrombosis in order to relieve COVID-19 [130] | → | 2 [3] | increased risks of preeclampsia as one of the most challenging problems of modern obstetrics [131] | ← |
| *66* | *F3* | 2 [3] | ozone therapy suppresses F3 and, thereby, prevents thrombotic ischemic intestinal damage [132] | → | 5 [3] | increased risks of stroke and myocardial infarction as two most often causes of human death [133] | ← |
| *67* | *F7* | 2 [3] | increased risks of episodic spontaneous difficult to stop life-threatening bleeding [134] | ← | 5 [3] | recombinant activated F7 is a life-saving drug for obstetric life-threatening bleeding [135] | → |
| *68* | *F8* | | hemophilia A: spontaneous hemorrhages in the brain, joints, muscles, internal organs and, as a result, disability [136] | ← | 1 [3] | increased risks of thrombosis provoking stroke and myocardial infarction as the two most frequent causes of death in humans [137] | ← |
| *69* | *F9* | 1 [3] | hemophilia B: spontaneous hemorrhages in the brain, joints, muscles, internal organs and, as a result, disability [138] | ← | 1 [3] | increased risks of myocardial fibrosis causing tachyarrhythmias, disability *via* heart failure and, ultimately, cardiovascular death [139] | ← |
| *70* | *F11* | 1 [3] | coagulation factor XI insufficiency provoking spontaneous bleeding and, ultimately, disability [140] | ← | 5 [3] | increased risks of angioedema provoking hypercapnic coma in case of carbon dioxide poisoning and, as a result, death [141] | ← |
| *71* | *FGF1* | 6 [4] | within a retrospective review of publications on wound healing in rats and mice as human disease models, delayed skin wound healing [142] | ← | 5 [4] | in human disease models using mice with artificial skin wounds treated with bacterial plasmid carrying FGF1 gene, improved wound healing [143] | → |
| *72* | *FGF3* | | within a model of human cord brain injury using zebrafish, inhibited formation of so-called "glial bridge" and prevented axon regeneration [144] | ← | 1 [4] | within a model of human cord brain injury using zebrafish, improved post-traumatic neuron regeneration [144] | → |
| *73* | *FGF4* | | within human disease models in vitro, miR-511 inhibits breast cancer proliferation and metastasis by down-regulating FGF4 expression [145] | → | 2 [4] | within a model of human diseases using mice with artificial brain injury, improved post-traumatic neural tissue survival [146] | → |
| *74* | *FGF5* | 1 [4] | in human cancer models *in vitro* using non-small cell lung cancer cells, prevented the cancer cell proliferation, migration and invasion [147] | → | 1 [4] | in human disease models using mice treated with intranasal *Aspergillus fumigatis*, tissue remodeling as complications of chronic inflammation [148] | ← |
| *75* | *FGF6* | 2 [4] | in human disease models using mice at artificial injury treated with clodronate-containing liposomes, worsen skeletal muscle regeneration [149] | ← | 1 [4] | in human disease models using mice treated with intranasal *Aspergillus fumigatis*, tissue remodeling as complications of chronic inflammation [148] | ← |
| *76* | *FGF8* | 2 [4] | within a model of human diseases using Fgf8-deficient mice, increased risks of stress-induced anxiety-like behavior [149] | ← | 4 [4] | in human disease models using transgenic mice carrying Fgf8 under mouse mammary tumor virus promoter, increased risks of breast cancer [150] | ← |

***Genes***: *DRD2, DRD3, DRD4,* and *DRD5,* dopamine receptors D2, D3, D4, and D5, respectively; *ESR2,* estrogen receptor 2 (β); *F2, F3, F7, F8, F9,* and *F11,* coagulation factors II (synonym: thrombin), III (synonyms: thromboplastin, tissue factor), VII (synonym: proconvertin), VIII (synonym: hemophilia A), IX (synonym: hemophilia B), and XI, respectively; *FGF1, FGF3, FGF4, FGF5, FGF6,* and *FGF8,* fibroblast growth factors 1, 3, 4, 5, 6, and 8, respectively.

**Table S1.** Cont.

| # | Human Gene | Deficit (↓) | | | Excess (↑) | | |
|---|---|---|---|---|---|---|---|
| | | $N_{SNP}$ | Effect on reproductive potential [Reference] | ♂♀ | $N_{SNP}$ | Effect on reproductive potential [Reference] | ♂♀ |
| 77 | *FGF9* | 1 [4] | in human disease models using young and aged mice carrying artificial skin injury, worsen post-traumatic wound healing [151] | ← | | in human male infertility models using adult male mice whos testis were injected with lentivirus carrying *Fgf9*, arrested spermatogenesis [152] | ← |
| 78 | *FGF10* | | in human newborn model using Fgf10-null mice, a congenital duodenum obstruction that needs surgery in just the 1st day of life [153] | ← | 2 [4] | in human disease models using young and aged mice carrying artificial skin injury, improved post-traumatic wound healing [151] | → |
| 79 | *FGF11* | 1 [4] | in human embryogenesis models using mice embryo carrying siRNA-caused Fgf11-knockdown, hampered forelimb bud development [154] | ← | 1 [4] | in human disease models using fibroblast cells embedded into alginate scaffold with interconnected pores, better post-injury skin repair [155] | → |
| 80 | *FGF12* | 3 [4] | in human embryogenesis models using rats, slowed increase in aversive behavior from generation to generation as response to prenatal stress [156] | → | 5 [4] | within a human subfertile female cohort-based clinical study, raised risks of adiposity worsening folliculogenesis & oocyte quality [157] | ← |
| 81 | *FGF13* | 3 [4] | within a model of human diseases using FGgf13-knockout mice, increased risks of both obesity and inability to keep core temperature [158] | ← | 4 [4] | in human disease models using rats infected with lentivirus carrying *Fgf13*, improved post-injury axon repair, regeneration and regrowth [159] | → |
| 82 | *FGF14* | | in human disease models using in vivo FGF14-knockdown in adult Purkinje neurons, worsen motor activity, coordination and balance [160] | ← | 12 [4] | in human tumorigenesis models using lung tumor cells *ex vivo*, improved tumor suppression [170] | → |
| 83 | *FGF17* | 1 [4] | in human behavior models using Fgf17-deficient mice, reduced social contacts within opposite-sex pairs to explore a novel environment [171] | ← | 2 [4] | within a human male cohort-based clinical study, increased risks of prostate cancer compared to benign prostatic hyperplasia [172] | ← |
| 84 | *FGF18* | 1 [4] | within a model of human newborn using mice carrying a germline Fgf18-knockdown: died shortly after birth [173] | ← | | in human behavior models using Fgf18-infusion into mice with cerebral ischemia: better cerebral blood flow, memory and motor abilities [174] | → |
| 85 | *FGF19* | | within a human cohort-based clinical study, increased risks of coronary artery disease, which severity raises with FGF19-deficit raising [175] | ← | 1 [4] | clinical ursodeoxycholic acid treatment reduces both itch and bile acid level in intrahepatic cholestasis of pregnancy through FGF19-upregulation [176] | → |
| 86 | *FGF20* | 1 [4] | within a model of human health models using Fgf20-deficient mice, impaired mammary gland morphogenesis during puberty [177] | ← | 9 [4] | in human disease models using rats with artificial brain trauma treated with Fgf20 supra-nigral infusion: neuroprotection for fine motor movement [178] | → |
| 87 | *FGF21* | | within a model of human health using muscle-specific Fgf21-null mice, reduced risks of muscle loss and weakness during fasting [179] | → | 2 [4] | clinical valproate treatment against depression improves mood & metabolic states simultaneously in patients through FGF21-level increase [180] | → |
| 88 | *FGF22* | | within a cohort-based clinical patient study, increased risks of depression [181] | ← | 4 [4] | in human chronic unpredictable mild stress models using mice treated with injections of lentiviral vector carrying FGF22, alleviated depression [181] | → |
| 89 | *FGFR1* | 3 [4] | within a model of human embryogenesis using Fgfr1-null mice, where embryos displayed early growth defects and, eventually, lethality [182] | ← | 10 [4] | in human disease models using mice with artificial vocal fold injury treated by platelet-rich plasma: wound healing without scar due to *Fgfr1* upregulation [183] | → |
| 90 | *FGFR2* | 10 [4] | within a model of women firtility using defective Fgfr1-mutant female mice, increased risks of both subfertility and pregnancy loss [184] | ← | 11 [4] | within a cohort-based clinical gastric cancer patient study, higher risks of the primary gastric tumors with poor relapse-free survival [185] | ← |
| 91 | *FGFR3* | | within a model of human diseases using mice carrying fibroblast-specific Fgfr3-knockdown, attenuated experimental skin fibrosis [186] | → | 8 [4] | within a model of human diseases using teratoma-susceptible mice strain 129/SvJ, higher risks of mitotic arrest in fetal male germ cells [187] | ← |
| 92 | *FGFR4* | 4 [4] | in human disease models using adult Fgfr4-null mice: increased risks of airway inflammation, bronchial obstruction, and right ventricular hypertrophy [188] | ← | 1 [4] | within a cohort-based clinical facioscapulohumeral muscular dystrophy study, increased risks of both muscle fibrosis and disease severity [189] | ← |
| 93 | *FGFRL1* | 3 [4] | in human embryogenesis models using Fgfrl1-knockout mice, newborn lethality through malformed diaphragm & lack metanephric kidneys [190] | ← | 6 [4] | within a model of lung cancer using a qPCR-based comparison between human's lung cancer and lung norm cell lines: suppressed metastasis [191] | → |
| 94 | *FLT1* | 3 [4] | within a cohort-based clinical study, reduced risks of preeclampsia as one of the main causes of maternal and neonatal mortality in the world [192] | → | 3 [4] | within a cohort-based clinical study, increased risks of preeclampsia as one of the main causes of maternal and neonatal mortality in the world [192] | ← |

***Genes***: *FGF9*, *FGF10*, *FGF11*, *FGF12*, *FGF13*, *FGF14*, *FGF17*, *FGF18*, *FGF19*, *FGF20*, *FGF21*, and *FGF22*, fibroblast growth factors 9, 10, 11, 12, 13, 14, 17, 18, 19, 20, 21, and 22, respectively; *FGFR1*, *FGFR2*, *FGFR3*, and *FGFR4*, fibroblast growth factor receptors 1, 2, 3, and 4, respectively; *FGFRL1*, fibroblast growth factor receptor like protein 1; *FLT1*, Fms-related receptor tyrosine kinase 1,.

**Table S1.** Cont.

| # | Human Gene | Deficit (↓) | | | Excess (↑) | | |
|---|---|---|---|---|---|---|---|
| | | $N_{SNP}$ | Effect on reproductive potential [Reference] | ♂♀ | $N_{SNP}$ | Effect on reproductive potential [Reference] | ♂♀ |
| 95 | *FLT4* | 2 [4] | FLT4-blockade suppresses metastasis of melanoma cells by impaired lymphatic vessels [193] | → | 11 [4] | increased risks of inflammatory neovascularization after injury [194] | ← |
| 96 | *FLT3LG* | 2 [4] | within a model of human diseases using Flt2lg-knockout mice: reduced severity of artificial hepatic ischemia/reperfusion injury [195] | → | 4 [4] | in human disease models using artificially burn-injured mice with injection of recombinant FLT3LG, resistant to wound infections [196] | → |
| 97 | *FOS* | 4 [2] | in human obesity model using mice hypothalamic cells, Fos-inhibitor T-5224 regains gonadotropin-releasing hormone level as fertility [197] | → | 8 [2] | improved maturation of oocytes [198] | → |
| 98 | *GABARAP* | 1 [4] | within a model of human diseases using adult male rats , reduced both fear-potentiated startle and fear-memory [199] | → | 3 [4] | within a model of human diseases using HEK293 cells, hypersensitivity to pain caused by physical stimuli [200] | ← |
| 99 | *GABARAPL1* | 9 [4] | in human disease models using rats with low-protein diet during pregnancy and lactation, higher risks of arterial hypertension in adult offsprings [201] | ← | 10 [4] | within a model of human health using mice performing a low-intensity running exercise, reduced muscle endurance [202] | ← |
| 100 | *GABARA PL2* | 4 [4] | retarded both neutrophilic differentiation and wound healing [203] | ← | 10 [4] | improved autophagy within odontogenic differentiation of dental pulp cells during healing a tooth injury [204] | → |
| 101 | *GABBR1* | 1 [4] | within a cohort-based clinical study, incresed risks of schizophrenia , bipolar disorder, and major depression [205] | ← | 1 [4] | in human disease models using transgenic mice injected with pCI-vector (Promega) carrying Gabbr1, reduced pathological pain sensitivity [206] | → |
| 102 | *GABBR2* | 1 [4] | within a cohort-based clinical study, incresed risks of schizophrenia , bipolar disorder, and major depression [205] | ← | 5 [4] | in human disease models using mice with artificial injury of vestibular labyrinth and Gabbr2-agonist: accelerated repair of gait and reflexes [207] | → |
| 103 | *GABRA1* | 4 [4] | in human disease models using pregnant mice injected with valproic acid as autism spectrum disorder inductor: social disorders in offsprings [208] | ← | 4 [4] | in human disease models using mutant mice Gabba1:270S>H increasing GABA-sensitivity: less body size, motor coordination and viability [209] | ← |
| 104 | *GABRA2* | 5 [4] | in human disease models using mice with artificial sciatic nerve injury treated, where Gabra2-antisence worsens pain hypersensitivity [210] | ← | 7 [4] | in human behavior models using chronic social defeat stress in male mice, increased risks of mixed anxiety/depression-like state [211] | ← |
| 105 | *GABRA5* | 2 [4] | in human behavior models using rats infused with chloroform, increased risks of both memory impairment and learned helplessness [212] | ← | 12 [4] | within a model of human neuropsychiatric and neurodevelopmental disorders using mutant mice, higher risk of anxiety-like behavior [213] | ← |
| 106 | *GABRA6* | 1 [4] | in human pregnancy models using vitamin C-deficient pregnant mice supplemented with vitamin C (norm) and without it: higher risk of stillbirths [214] | ← | 6 [4] | a cohort-based clinical study of SNPs within miRNAs, norm of which represses GABRA6, associated them with panic disorder [215] | ← |
| 107 | *GABRB1* | 2 [4] | within a retrospective review of many cohort-based clinical studies, increased risks of epilepsy, autism, bipolar disorder and schizophrenia [216] | ← | | only simultaneous silencing of gabrb1 expression and gabrb1-protein inhibition lows the prion protein level in neuroblastoma cells [217] | ← |
| 108 | *GABRB2* | 3 [4] | in human disease models using pregnant mice injected with valproic acid as autism spectrum disorder inductor: social disorders in offsprings [208] | ← | 3 [4] | in human pregnancy models using rats with artificial flurothyl-induced neonatal recurrent seizures: accelerated developing brain injury [218] | ← |
| 109 | *GABRB3* | 5 [4] | within a model of human embryogenesis using Gabrb3-knockout mice, increased risks of either newborn lethality or reduced life span [219] | ← | 7 [4] | in human disease models using pregnant mice at ethanol diet, higher risk of autism-like asocial behavior and memory deficits in male offsprings [220] | ← |
| 110 | *GABRD* | | within a cohort-based clinical spermatozoa-related study, increased risks of male infertility [221] | ← | 1 [4] | within a model of human activity-based anorexia using adolescent mice, higher risk of stress-caused anxiety and weight loss in adolescence [222] | ← |
| 111 | *GABRE* | 2 [4] | in human pregnancy model using female rats administered with Gabre-agonist, increased risks of life-threatening respiratory rhythm disorder [223] | ← | | within a model of human pentobarbital side effects using rat neurons transfected with adenovirus carrying Gabre, cardioprotection [224] | → |

__*Genes:*__ *FLT4*, Fms-related receptor tyrosine kinase 4; *FLT3LG*, Fms-related receptor tyrosine kinase 3 ligand; *FOS*, AP-1 transcription factor subunit Fos proto-oncogene; *GABARAP*, gamma-aminobutyric acid type A (GABA(A)) receptor-associated protein; *GABARAPL1* and *GABARAPL2*, GABA(A) receptor-associated protein like proteins 1 and 2, respectively; *GABBR1* and *GABBR2*, GABA(B) receptor subunits 1 and 2, respectively; *GABRA1, GABRA2, GABRA5,* and *GABRA6,* GABA(A) receptor subunits $\alpha$1, $\alpha$2, $\alpha$5, and $\alpha$6, respectively; *GABRB1, GABRB2, GABRB3, GABRD, and GABRE,* GABA(A) receptor subunits β1, β2, β3, δ, and ε, respectively.

**Table S1.** Cont.

| # | Human Gene | Deficit (↓) | | | Excess (↑) | | |
|---|---|---|---|---|---|---|---|
| | | $N_{SNP}$ | **Effect on reproductive potential [Reference]** | ♂♀ | $N_{SNP}$ | **Effect on reproductive potential [Reference]** | ♂♀ |
| *112* | *GABRG1* | | in human disease models using sodium-butyrate treated mice administered with Gabg1-agonist, higher risk of asocial autism-like behavior [225] | ← | 1 [4] | within a model of human disease using sodium-butyrate treated mice, improved social behavior [225] | → |
| *113* | *GABRG2* | 2 [4] | within a model of human diseases using Gabrg2-knockout mice, increased risks of asocial depressive behavior [226] | ← | 1 [4] | within a model of human diseases using male mice stressed in infant age, increased risks of anxiety-like asocial behavior in adult age [227] | ← |
| *114* | *GABRG3* | 3 [4] | within a model of human diseases using mice carrying natural Gabrg3-deletion, higher risk of both Angelman's and Prader-Willi's syndromes [228] | ← | 9 [4] | in human disease models using artificial temporal hearing loss in rats, increased risks of hyperacusis up to asocial depressive behavior [229] | ← |
| *115* | *GABRP* | 3 [4] | within a cohort-based clinical study of cervical cancer women overexpressing microRNA-320c targeting GABRP, reduced risks of metastasis [230] | → | 1 [4] | within a metastatic xenograft mouse model using human ovarian carcinoma SK-OV-3 cells, increased risks of metastasis [231] | ← |
| *116* | *GABRR1* | | within a model of human behavior using Gabrr1-knockout mice, increased risks of mechanical pain hypersensitivity [232] | ← | 1 [4] | in human health models using CD34+ cells treated with GABRR1 agonists: improved megakaryocyte colonies as sources for platelet in blood [233] | → |
| *117* | *GABRR2* | 1 [4] | within a model of human diseases using rats studied with polyclonal GABRR2-antibodies: increased threshold for mechanical pain [234] | → | | within a model of human pregnancy using pregnant rats: improved "maternal brain" development and, thus, "maternal behavior" [235] | → |
| *118* | *GABRR3* | 2 [4] | within a cohort-based clinical human blood samples study, worsen healing of wounds owing to accelerated platelet senescence [236] | ← | 2 [4] | in human disease models using mice treated with polyinosinic-polycytidylic acid, hypersusceptibility to excitotoxic brain insult [237] | ← |
| *119* | *GCG* | 2 [3] | reduced pregnancy rate [238] | ← | | reduced pregnancy rate [239] | ← |
| *120* | *GDNF* | 1 [4] | within a model of human spermatogenesis using mice: hindered spermatogonial stem cells self-renewal and impaired fertility [240] | ← | 1 [4] | in human spermatogenesis models using mice spermatogonial progenitor cells and GDNF: improved proliferation of these cells & male fertility [241] | → |
| *121* | *GFRA1* | 2 [4] | in human spermatogenesis models using adult male rats treated with pyrethroids: impaired sperm production and development[242] | ← | 1 [4] | in human spermatogenesis models using mice pup spermatogonial progenitor cells treated with diethylstilbestrol: testicular cancer in adult [243] | ← |
| *122* | *GFRA2* | | within a model of human diseases using Gfra2-knockout mice, impaired gastrointestinal transit rate [244] | ← | 1 [4] | within human disease models using mice, improved post-injury survival of all motoneurons except oculomotor and abducens nerves [245] | → |
| *123* | *GFRA3* | 1 [4] | increased risks of somatosensory system neurodegeneration [246] | ← | | improved neural regeneration after injury [247] | → |
| *124* | *GFRA4* | 3 [4] | within a model of human diseases using GFRA4-knockout mice, calcitonin deficit rises prematurely bone formation rate in adolescent [248] | ← | 2 [4] | improved neuronal survival and neurite outgrowth [249] | → |
| *125* | *GH1* | 2 [3] | increased mortality from cardiovascular disease [250] | ← | 2 [3] | somatotropin is used as a drug to prolong the reproductive age in women [251] | → |
| *126* | *GJA5* | 3 [3] | increased risks of the heart morphogenesis disorders, which result in arrhythmias and cardiovascular diseases [252] | ← | | increased arteriogenesis as the human body response to a low oxygen level at chronic hypoxia [253] | → |
| *127* | *GMFB* | 2 [4] | within a model of human diseases using Gmfb-knockout mice, reduced neurodegenerative effects on motor coordination in brain injury [254] | → | | in human disease models using male mice with artificial brain injury: increased risks of lung injury as a complication of brain injury [255] | ← |
| 128 | GMFG | 2 [4] | within a model of human muscle ischemic injury complications using human cardiomyocyte cells, increased risks of necrosis [256] | ← | 4 [4] | in women fertility models using both granulosa and theca cells from antral bovine follicles, improved ovarian functions [257] | → |

***Genes***:, *GABRG1, GABRG2, GABRG3, GABRP, GABRR1, GABRR2,* and *GABRR3*, GABA(A) receptor subunits γ1, γ2, γ3, π, ϱ1, ϱ2, and ϱ3, respectively; *GCG*, glucagon; *GDNF*, glial cell derived neurotrophic factor; *GFRA1, GFRA2, GFRA3,* and *GFRA4*, glial cell line-derived neurotrophic factor receptor α1, α2, α3, and α4, respectively; *GH1*, growth hormone 1 (synonym: somatotropin); *GJA5*, connexin 40 (synonym: gap junction protein α5); *GMFB* and *GMFG*,, glia maturation factor β and γ, respectively.

**Table S1.** Cont.

| # | Human Gene | Deficit (↓) | | | Excess (↑) | | |
|---|---|---|---|---|---|---|---|
| | | *N<sub>SNP</sub>* | Effect on reproductive potential [Reference] | ♂♀ | *N<sub>SNP</sub>* | Effect on reproductive potential [Reference] | ♂♀ |
| *129* | *GPR18* | 2 [4] | in human disease models using rats with artificial ischemic stroke administered with GPR18-antagonist, worsen post-injury repair [258] | ← | 1 [4] | in human disease models using rats with artificial injury treated with N-arachidonoyl-serotonin, increased pain sensitivity threshold [259] | → |
| *130* | *GPR55* | 1 [4] | within a model of women infertility using female mice administered with GPR55-antagonist, impaired oocyte maturation [260] | ← | 4 [4] | in human disease models using rats with intravenous administration of GPR55-receptor agonist, reduced pain-related behavior [261] | → |
| *131* | *GPR119* | 2 [4] | within a model of human diseases using Gpr119-knockout mice: resistant to hypophagia due to normalized gut hormone levels and food intake [262] | → | 2 [4] | in human obesity models using high-fat-fed rats with oral administration of GPR119-agonist: reduced both food intake and body weight gain [263] | → |
| *132* | *GRIA1* | 2 [4] | within a retrospective clinical cohort-based transcriptome meta-analysis, increased risks of polycystic ovary syndrome [264] | ← | 3 [4] | in human behavior models using pregnant rats at a high-caloric palatable diet, increased risks of anxiety in male offsprings [265] | ← |
| *133* | *GRIA2* | 7 [4] | within a model of human behavior models using Gria2-knockout mice: impaired stimulus-reward learning with conditioned stimuli[266] | ← | 10 [4] | within a retrospective clinical cohort-based transcriptome meta-analysis, increased risks of uterine leiomyomas and infertility [267] | ← |
| *134* | *GRIA4* | 2 [4] | within a model of human behavior using Gria4-knockout mice, improved spatial working memory and reduced fright behavior [268] | → | 6 [4] | within a clinical cohort-based transcriptome of postmortem subjects, increased risks of major depressive disorder up to suicide [269] | ← |
| *135* | *GRIK1* | 1 [4] | within a model of human behavior using Grik1-knockout mice: reduced sensitivity to itching and scratch pain that improves wound healing [270] | → | 5 [4] | within a model of human behavior using mice treated with pharmacological Grik1-activation: pain hypersensitivity [271] | ← |
| *136* | *GRIK2* | 6 [4] | based on a case of father carrying a GRIK2-damage: increased risks of stillbirth, miscarriage, as well as son severe intellectual disability [272] | ← | 3 [4] | in human behavior model using rats injected with formalin into rectum mucosa: hypersensitivity to acute inflammatory pain [273] | ← |
| *137* | *GRIK3* | | based on a case of girl carrying a GRIK3-damage: increased risks severe developmental delay affecting language and fine motor skills [274] | ← | 1 [4] | within a clinical cohort-based transcriptome of postmortem subjects, increased risks suicide in major depressive disorder [269] | ← |
| *138* | *GRIK5* | 1 [4] | within a model of human aging using mice administered with D-galactose in order to induce aging artificially: delayed aging [275] | → | 3 [4] | according to a pharmaceuticals report, $\gamma$-aminobutyric acid treats bipolar disorder through an GRIK5-upregulation [276] | ← |
| *139* | *GRIN1* | 1 [4] | within a model of human pregnancy using pregnancy rats under artificial hypoxia, impaired learning and memory ability in adolescent offspring [277] | ← | 8 [4] | in human behavior models using pregnant rats at a high-caloric palatable diet, increased risks of anxiety in male offspring [265] | ← |
| *140* | *GRIN2A* | 2 [4] | in human behavior models using mice under scream sound stress during 21 postnatal days, impaired spatial learning and memory in adult males [278] | ← | 2 [4] | in human behavior models using transgene mice overexpressing Grin2a in neurons, improved long-term fear memory [279] | ← |
| *141* | *GRIN2C* | 2 [4] | in human pregnancy models using pregnant mice with voluntary alcohol drinking, anxiety and learning deficits in adolescent male offspring [280] | ← | | within a model of human disease using mice infused with Grin2C-agonists, improved motor function [281] | → |
| *142* | *GRIN2D* | | within a model of human behavior using Grin2d-knockout male mice: exacerbated negative emotional behavior [282] | ← | 4 [4] | within a model of human disease using rats with artificial pulpitis with bacterial infection, neuropathic pain hypersensitivity [283] | ← |
| *143* | *GRIN3A* | | in a model of human drug addiction using Grin3a-knockout mice, no cocaine-caused long-term burst firing of dopamine neurons [284] | → | 1 [4] | increased risks of inattentive behavior [285] | ← |
| *144* | *GRIN3B* | | within a model of human behavior using Grin3b-knockout mice: significant impairment in motor learning or coordination [286] | ← | 4 [4] | in human behavior models using norm *versus* Grin3b-knockout mice: improved motor learning or coordination and reduced anxiety [286] | ← |
| *145* | *GRINA* | 3 [4] | within a clinical cohort-based pharmacological study, topiramate as a drug reduces drug addiction through GRINA-downregulation [287] | ← | 8 [4] | within a clinical cohort-based postmortem study, increased risks of major depressive disorder [288] | ← |

***Genes***: *GPR18*, *GPR55*, and *GPR119*, G protein-coupled receptors 18, 55, and 119, respectively; *GRIA1*, *GRIA2*, and *GRIA4*, glutamate ionotropic receptor AMPA type subunits 1, 2, and 4, respectively; *GRIK1*, *GRIK2*, *GRIK3*, and *GRIK5*, glutamate ionotropic receptor kainate type subunits 1, 2, 3, and 5, respectively; *GRIN1*, *GRIN2A*, *GRIN2C*, *GRIN2D*, *GRIN3A* and *GRIN3B*, glutamate ionotropic receptor NMDA type subunits 1, 2A, 2C 2D, 3A, and 3B; *GRINA*, glutamate ionotropic receptor NMDA type subunit associated protein 1.

**Table S1.** Cont.

| # | Human Gene | Deficit (↓) | | | Excess (↑) | | |
|---|---|---|---|---|---|---|---|
| | | $N_{SNP}$ | Effect on reproductive potential [Reference] | ♂♀ | $N_{SNP}$ | Effect on reproductive potential [Reference] | ♂♀ |
| *146* | *GRM1* | | within a pharmacological clinical study, isoflurane as a drug protects the myocardium against ischemia and injury by upregulating GRM1 [289] | ← | 4 [4] | within a clinical cohort-based transcriptome of postmortem subjects, increased risks of major depressive disorder up to suicide [269] | ← |
| *147* | *GRM2* | 1 [4] | within a model of human behavior using rats microinjected with GRM2-antagonist: increased risks of fearful motivation in form of burying [290] | ← | 1 [4] | within a model of human diseases using human sensory neurons from donors without a history of chronic pain: reduced pain sensitivity [291] | → |
| *148* | *GRM3* | 1 [4] | within a model of human behavior using Grm3-knockout mice: increased risks of schizophrenia-like hyperactive asocial aggressive behavior [292] | ← | 3 [4] | within a model of human behavior using normal mice administered with selective negative allosteric Grm3-modulator: worsen learning [293] | → |
| *149* | *GRM4* | 2 [4] | weakened microglial inflammation during post-injury brain repair [294] | → | 5 [4] | increased risks of depression-like behavior [295] | ← |
| *150* | *GRM5* | 2 [4] | in human behavior models using mice administered with Grm5-antagonist: reduced pain sensitivity [296] | → | 3 [4] | in human behavior models using pregnant rats exposed to repeated episodes of restraint stress: reduced stress resilient in adult offspring [297] | ← |
| *151* | *GRM7* | 3 [4] | within a model of men subfertility using adult male Grm7-knockout mice: subfertility, lowed insemination capability, excess defective spermatozoa [298] | ← | 6 [4] | in human embryo models using *Grm7*-knockdown pregnant mice injected with plasmid carrying *Grm7* into fetal brain: ameliorated neurogenesis defects [299] | → |
| *152* | *GRM8* | 4 [4] | within a model of human behavior using Grm8-deficient mice: dramatic reduction in contextual fear [300] | → | 5 [4] | in human behavior models using rats exposed with artificial injury and Grm8-agonist microinjection into brain: reduced pain sensitivity [301] | ← |
| *153* | *GSTM3* | 2 [3] | increased risk of non-obstructive azoospermia [302] | ← | 2 [3] | within human diseases model using cows, increased frequency of natural fertilization compared to artificial fertilization [303] | → |
| *154* | *GTPBP6* | 3 [5] | increased intelligence quotient IQ scores in men [304] that is negatively significantly associated with amount of their siblings and cousins [305] | ← | 3 [5] | reduced intelligence quotient IQ scores in men [304] that is positively significantly associated with amount of their siblings and cousins [305] | → |
| *155* | *HBB* | 9 [3] | thalassemia impairs women's reproductive health [306] | ← | | within traditional Chinese medicine, Jian-Pi-Yi-Sheng decoction (JPYS) rises hemoglobin to treat anemia in chronic kidney diseases [307] | → |
| *156* | *HBD* | 2 [3] | thalassemia impairs women's reproductive health [306] | ← | | within code for hemoglobin subunits α, β [307] | → |
| *157* | *HBG2* | 1 [3] | thalassemia impairs women's reproductive health [306] | ← | | within traditional Chinese medicine, Jian-Pi-Yi-Sheng decoction (JPYS) rises hemoglobin to treat anemia in chronic kidney diseases [307] | → |
| *158* | *HSD17B1* | 3 [3] | increased risk of breast cancer [308] | ← | 1 [3] | increased risk of breast cancer [309] | ← |
| *159* | *HTR1A* | 1 [4] | within a model human behavior using Htr1a-knockout mice: increased risks of anxiety-like behavior [310] | ← | 1 [4] | in human behavior models using chicken embryos exposured with corticosterone during incubation: aggressiveness in chicks [311] | → |
| *160* | *HTR1B* | | within a model human behavior using Htr1b-knockout mice: increased risks of aggressive behavior [312] | → | 3 [4] | within human behavior models using transgene mice infected with viral vector carrying Htr1b: increased risks of stress-induced anxiety [313] | ← |
| *161* | *HTR1F* | 1 [4] | in human milk feeding baby models using dairy calves fed with milk and serotonin precursor: better serotonergic regulation of energy metabolism [313] | → | | within a model human behavior using rats injected with formalin and, next, administered Htr1f-agonist: reduced inflammatory pain [314] | ← |
| *162* | *HTR2A* | 1 [4] | within a model human disease using adipose tissue-specific Htr2a-knockout mice: resistance to obesity during high-fat diet [315] | → | | within a clinical cohort-based study, increased risks of hypertrophic hearts as the leading cause of sudden death in young athletes [316] | ← |

***Genes***: *GRM1*, *GRM2*, *GRM3*, *GRM4*, *GRM5*, *GRM7*, and *GRM8*, glutamate metabotropic receptors 1, 2, 3, 4, 5, 7, and 8, respectively; *GSTM3*, glutathione S-transferase μ3; *GTPBP6*, GTP-binding protein 6; *HBB*, *HBD*, and *HBG2*, hemoglobin subunits β, δ, and γ2, respectively; *HSD17B1*, hydroxysteroid 17β dehydrogenase 1; *HTR1A*, *HTR1B*, *HTR1F*, and *HTR2A*, 5-hydroxytryptamine receptor 1A., 1B, 1F, and 2A, respectively.

**Table S1.** Cont.

| # | Human Gene | Deficit (↓) | | | Excess (↑) | | |
|---|---|---|---|---|---|---|---|
| | | $N_{SNP}$ | Effect on reproductive potential [Reference] | ♂♀ | $N_{SNP}$ | Effect on reproductive potential [Reference] | ♂♀ |
| 163 | *HTR2C* | | in human health models using neuroblastoma cells treated with psychoactive drugs at environmental levels: higher risk of autism spectrum disorders [317] | ← | 1 [4] | within a model human behavior using tame and aggressive rats bred artificially, inhibited fear-evoked aggressive behavior [318] | ← |
| 164 | *HTR3A* | 3 [4] | increased risks of sudden cardiac death during pregnancy [319] | ← | | within a model of human health using mice, improved hippocampal neurogenesis and antidepressant effects caused by physical exercise [320] | → |
| 165 | *HTR3B* | 1 [4] | within a model of human's both 'anger-in' and 'anger-out' emotions using rats, decreased risks of anger-related resolute behavior [321] | ← | | reduced risks of pulmonary embolism, severe cases of which can lead to passing out, abnormally low blood pressure, and sudden death [322] | → |
| 166 | *HTR3C* | 1 [4] | within a model of human diseases using HEK293 cell line treated with antibody against HTR3C: enhanced aggressive behavior [323] | → | 2 [4] | within a model of human diseases using HEK293 cell line treated with antibody against HTR3C: reduced aggressive behavior [323] | ← |
| 167 | *HTR3D* | 1 [4] | within a model of human diseases using HEK293 cell line treated with antibody against HTR3D: reduced aggressive behavior [323] | ← | 2 [4] | within a model of human diseases using HEK293 cell line treated with antibody against HTR3D: enhanced aggressive behavior [323] | → |
| 168 | *HTR3E* | 4 [4] | within a model of human diseases using HEK293 cell line treated with antibody against HTR3E: reduced aggressive behavior [323] | ← | 1 [4] | within a model of human diseases using HEK293 cell line treated with antibody against HTR3E: enhanced aggressive behavior [323] | → |
| 169 | *HTR4* | 2 [4] | within a model of human behavior using mice, increased risks of depression, anxiety and affective disorders [324] | ← | 13 [4] | within a model of human newborns using neonatal calves, improved development of the immune system and gastrointestinal tract [325] | → |
| 170 | *HTR5A* | | within a model of human newborns using pregnant Brahman cows under stress, elevated temperament scores in male offspring [326] | → | 1 [4] | within a clinical cohort-based pregnant women study, increased risks of fetal growth restriction [327] | ← |
| 171 | *HTR7* | | within a clinical cohort-based irritable bowel syndrome study, visceral abdominal pain hypersensitivity up to sexual disfunction [328] | ← | 1 [4] | in human lactogenesis models using pregnant rats under high-fat diet, delayed lactogenesis onset leading to mammary gland inflammation [329] | ← |
| 172 | *IGF1* | 2 [4] | within a model of women fertility using Holstein Friesian cows, imbalanced transition from pregnancy to lactation up to subfertility in future [330] | ← | | within a model of women fertility using female rats injected with human amnion epithelial cells in uterine, increase pregnancy rate [331] | → |
| 173 | *IGF2* | 2 [4] | within bioinformatics retrospective meta-analysis of the public biomedical databases, higher risk of diminished ovarian reserve up to subfertility [332] | ← | 1 [4] | in women fertility models using IGF2 supplementation in oocyte cultures from aged female mice, improved oocyte developmental competence [333] | → |
| 174 | *IGF1R* | 5 [4] | within a model of human embryogenesis using blastocysts from female rabbites with artificially induced type 1 diabetes: subfertility [334] | ← | 5 [4] | within a model of human embryogenesis using mice embryos under hypoxia: improved symmetric division during embryogenesis [335] | → |
| 175 | *IL1B* | 1 [3] | reduced risks of bone marrow hyperplasia and bone deformation in case of bacterial invasion [336] | → | 1 [3] | increased circadian hypersensitivity to pain [337] | ← |
| 176 | *IL3RA* | 2 [5] | within a human cancer model using acute myeloid leukemia cells, SS30 thioaptamer inhibites IL3RA that increases survival [338] | → | 3 [5] | increased risks of acute myeloid leukemia in children [339] | ← |
| 177 | *IL6* | 3 [4] | in human "mother-offspring" relationship models using pregnant pigs under alfalfa meal diet reducing IL6 level: more fertility and offspring survival [340] | → | 3 [4] | in women reproductive ageing models using immune cell populations from mice ovaries: accelerated decline in follicle number and oocyte quality [341] | ← |
| 178 | *IL6R* | 5 [4] | within a model of human diseases using transgenic mice under bacterial infection: reduced risks of tumorigenesis in chronic inflammation [342] | → | 5 [4] | within a clinical cohort-based study using peritoneal fluid from patients with versus without endometriosis, higher risk of endometriosis [343] | ← |
| 179 | *IL6ST* | 7 [4] | in a model of human diseases using mice, exacerbated inflammatory responses that is eventually, increased mortality during sepsis [344] | ← | 8 [4] | increases sensitivity to fatigue during submaximal exercise in sedentary middle-aged men (i.e., reproductive age men) [345] | ← |

***Genes***: *HTR2C, HTR3A, HTR3B, HTR3C, HTR3D, HTR3E, HTR4, HTR5A,* and *HTR7*, 5-hydroxytryptamine receptors 2C, 3A, 3B, 3C, 3D, 3E, 4, 5A, and 7, respectively; *IGF1* and *IGF2*, insulin-like growth factors 1 and 2, respectively (synonyms: somatomedin C and preptin, respectively); *IGF1R*, insulin like growth factor 1 receptor; *IL1B*, interleukin 1β; *IL3RA*, interleukin 3 receptor subunit α; *IL6, and IL6R*, interleukin 6 (synonym: interferon β2) and its receptor, respectively; *IL6ST*, interleukin 6 signal transducer.

**Table S1.** Cont.

| # | Human Gene | Deficit (↓) | | | Excess (↑) | | |
|---|---|---|---|---|---|---|---|
| | | $N_{SNP}$ | **Effect on reproductive potential [Reference]** | ♂♀ | $N_{SNP}$ | **Effect on reproductive potential [Reference]** | ♂♀ |
| *180* | *IL9R* | 1 [5] | trophoblast implantation impaired within preeclampsia [346] | ← | 1 [5] | increased risks of life-threatening anaphylactic shock [347] | ← |
| *181* | *IL11* | | in women fertility models using pregnant mice administered with IL11-blockator: reduced risks of pregnancy and preserved fertility in cancer [348] | → | 5 [4] | within a model of women fertility using transgenic female mice: enhanced decidualization [349] | → |
| *182* | *IL11RA* | | within a model of women fertility using Il11ra-knockout female mice: infertility through impaired decidualization [350] | ← | 7 [4] | within a model of human diseases using mice: increased risks of osteosarcoma [351] | ← |
| *183* | *IL27* | 1 [4] | within a clinical cohort-based study: increased risks of autoimmunity-related recurrent pregnancy loss [352] | ← | 1 [4] | within a clinical cohort-based study: increased risks of preterm birth through excessive inflammatory response in fetal membranes [353] | ← |
| *184* | *INS* | 1 [3] | within a model of human diseases using sheeps, hypoinsulinemia slows down fetal growth and development [354] | ← | 2 [3] | increased risks of neonatal diabetes mellitus, which can often progress to type I diabetes mellitus [355] | ← |
| *185* | *KDM5D* | 3 [5] | increased risks of aggressive prostate cancer [356] | ← | | increased risks of cardiovascular diseases [357] | ← |
| *186* | *LEP* | 1 [1] | increased risks of hypothalamic amenorrhea with dysfunction of hypothalamus endocrine axes and, ultimately, subfertility [358] | ← | 1 [1] | increased risks of subfertility as an obesity complication [359] | ← |
| *187* | *LGI4* | | within a clinical cohort-based loss-of-functuion LGI4 family study: increased risks of arthrogryposis multiplex congenita [360] | ← | 2 [2] | within a model of human behavior using tame and aggressive rats: increased risks of aggressive behavior [361] | → |
| *188* | *LIFR* | | within a clinical cohort-based infertile versus fertle women study: increased risks of infertility [360] | ← | 6 [4] | in human disease models using female rhesus macaque administered with soluble LIFR: increased risks of blocked ovulation [361] | ← |
| *189* | *MBL2* | 2 [3] | increased risks of recurrent late pregnancy losses at unclear etiology [362] | ← | 1 [3] | exogenous recombinant human MBL2 is used as a nonspecific immunomodulatory within adjuvant therapy against COVID-19 [363] | → |
| *190* | *MMP12* | 2 [3] | within models of human diseases using MMP12-knockout mice, low differentiation of oligodendrocytes of the central nervous system [364] | ← | | trophoblast implantation improved within pregnancy [365] | → |
| *191* | *MTHFR* | 2 [3] | increased risks of adverse pregnancy outcomes [366] | ← | 4 [3] | increased risks of preeclampsia as one of the most challenging problems of modern obstetrics [367] | ← |
| *192* | *NGFR* | | within a model of human embryogenesis using rat embryos: increased risks of fetal death [368] | ← | 3 [1] | in human disease models using newborn rats administered with estradiol valerate: increased risks of infertility in adul [369] | ← |
| *193* | *NLGN4Y* | 1 [5] | increased risks of both primary prostate cancer and its biochemically-induced recurrence [370] | ← | 2 [5] | increased risks of male infertility [371] | ← |
| *194* | *NOS2* | | in a model of human diseases using triple NOS1,2,3-knockout (because of their interchangeability) mice, reduced survival and fertility [372] | ← | 1 [3] | increased risks of diabetes mellitus in pregnancy, which is conventionally considered as pre-diabetes of both type I and II [374] | ← |
| *195* | *NPY* | 3 [4] | in a model of human health using Vgf-knockout mice, small size, low fat stores, hypermetabolism, hyperactivity, hypoleptinemia, infertility [375] | ← | 1 [4] | within a model of human obese using obese mice ob/ob line, rised risks of obesity, type 2 diabetes, and, eventually, subfertility [376] | ← |
| *196* | *NPY1R* | 7 [4] | within a model of human "mother-offspring" relationship using pregnant rats under high-fat diet embryos: increased risks of obesity in offspring [377] | ← | 5 [4] | within a model of human diseases using streptozotocin-induced type I diabetes in male rats: increased risks of type I diabetes [378] | ← |

***Genes***: *IL9R*, interleukin 9 receptor; *IL11* and *IL11RA*, interleukin 11 and its receptor subunit α, respectively; *IL27*, interleukin 27; *INS*, insulin; *KDM5D*, lysine demethylase 5D; *LEP*, leptin; *LGI4*, leucine-rich glioma-inactivated gene 4; *LIFR*, leukemia inhibitory factor receptor α; *MBL2*, mannose binding lectin 2; *MMP12*, matrix metallopeptidase 12 (synonym: macrophage elastase); *MTHFR*, methylenetetrahydrofolate reductase; *NGFR*, nerve growth factor receptor; *NLGN4Y*, neuroligin 4 Y-linked;; *NOS2*, nitric oxide synthase (inducible, hepatocytes, macrophage;); *NPY*, neuropeptide Y; *NPY1R*, neuropeptide Y receptors Y1.

**Table S1.** Cont.

| # | Human Gene | Deficit (↓) | | | Excess (↑) | | |
|---|---|---|---|---|---|---|---|
| | | $N_{SNP}$ | Effect on reproductive potential [Reference] | ♂♀ | $N_{SNP}$ | Effect on reproductive potential [Reference] | ♂♀ |
| 197 | *NPY2R* | | in human disease models using mice with artificial albuminuria treated with NPY2R-inhibitor: lesser risks of kidney failure and premature death [379] | → | 2 [4] | within a clinical cohort-based study: increased risks of depression-like behavior, which may be relieved due to antidepressants [380] | ← |
| 198 | *NPY4R* | | in human newborn models using newborn mice milk-fed with polychlorinated biphenyls at environmental levels: anxiety-like behavior in adulthood [381] | ← | 1 [4] | within a clinical cohort-based study on NPY4R copy number natural variation in-between 2 and 4: increased risks of obesity [382] | ← |
| 199 | *NPY5R* | | within a model of human obesity using Npy5r-knockout mice at high-fat diet with anorectic agents: resistance to anorectic drugs in obesity [383] | ← | 5 [4] | within a model of human pregnancy using mid- and late-pregnant rats: improved maternal behavior [79] | → |
| 200 | *NR5A1* | | gender-specifically increased risks of gonadal dysgenesis in men [384] | ← | 4 [3] | gender-specifically improved sexual determination/differentiation, adrenal and gonadal development in men [385] | → |
| 201 | *NRG1* | 5 [4] | within a model of human embryogenesis using tissue-specific Nrg1-knockout male mice embryos: impaired testis development [386] | ← | 18 [4] | in human disease models using juvenile mice administered with exogenous NRG1: higher risk of schizophrenia neuropathology [387] | ← |
| 202 | *NRG2* | 3 [4] | within a model of human behavior using Ng2-knockout mice: reduced anxiety-like behavior, hyperactivity and prepulse inhibition deficit [388] | → | 7 [4] | in human behavior models using high- and low-anxious male rats according to behavioral tests: higher risk of anxiety-like behavior [389] | ← |
| 203 | *NRG3* | 3 [4] | within a model of human behavior using Nrg3-knockout mice: hyperactivity, impaired prepulse inhibition and fear deficiency [390] | → | 1 [4] | within a model of human spermatogenesis using mice testis fragments cultured with NRG3: improved spermatogonia proliferation [391] | → |
| 204 | *NRG4* | | within a model of human obesity using tissue-specific conditional Nrg4-knockout mice: vascular rarefaction within adipose tissue in obesity [392] | → | 2 [4] | within a clinical cohort-based women study: increased risks of obesity-related polycystic ovary syndrome up to subfertility [393] | ← |
| 205 | *NRP1* | 12 [4] | within a model of women reproductive health using tissue-spesific Nrp1-knockdown mice: impaired ovariogenesis [394] | ← | 16 [4] | in human cancerogenesis models using human glioma cells exposed with synthetic NRP1-blocking peptides: retarded glioma growth [395] | ← |
| 206 | *NRP2* | 3 [4] | in models of human diseases using NRP2-knockout bladder cancer cell lines, improved patient survival in antitumor radiochemotherapy [396] | → | 1 [4] | increased risks of post-traumatic vascular neointimal hyperplasia [397] | ← |
| 207 | *NRTN* | 2 [4] | within a model of human diseases using Nrtn-knockout mice: the enteric nervous system defects (e.g., reduced gastrointestinal motility) [398] | ← | 1 [4] | in human behavior models using transgenic mice overexpressing Nrtn: behavioral hypersensitivity to environmental stimuli [399] | ← |
| 208 | *NTF3* | 1 [4] | within a clinical cohort-based study: increased risks of depressive behavior [400] | ← | 3 [4] | in human disease models using mice with artificial multiple sclerosis treated with full-term human placenta: Ntf3 excess biomarks this disease relief [401] | → |
| 209 | *NTF4* | | within a clinical cohort-based post-mortem brain study: increased risks of cognitive impairment [402] | ← | 2 [4] | in human "mother-offspring" relationship models using pregnant rats of low and high physical activity: improved learning and memory in offspring [403] | → |
| 210 | *NTRK1* | 1 [4] | within a clinical cohort-based study of women with silent NTRK1 gene versus norm: increased risks of external genital endometriosis [404] | ← | 1 [4] | in human pain sensitivity models using mice with artificially induced mechanical pain: reduced neuropathic allodynia [405] | ← |
| 211 | *NTRK2* | | within a model of women reproductive health using mutant Ntrk2-deficient female mice: post-pubertal oocyte death and early adulthood infertility [406] | ← | 4 [4] | in human "mother-offspring" relationship models using pregnant rats of low & high physical activity: tendency to improve learning & memory in offspring [403] | → |
| 212 | *NTRK3* | 3 [4] | in human diseases models using Ntrk3-knockout and Ntrk3-excessive mice: loss of kidney podocytes that accelerates aging through glomerular disease [407] | ← | 19 [4] | in human "mother-offspring" relationship models using pregnant mice infected artificially: higher risk of schizophrenia and autism in offspring [408] | ← |
| 213 | *OGFR* | 1 [4] | within a clinical case report on human male newborn carrying loss-of-function mutation within OGFR gene: lifelong inflammation of skin, bowel, & lungs [409] | ← | 4 [4] | within a model of human diseases using transgenic mice overexpressing Ogfr: impaired wound healing [410] | ← |
| 214 | *OPRD1* | | within a model of human behavior using Oprd1-knockout mice: increased risks of both anxiogenic- and depressive-like behavior [411] | ← | 1 [4] | in human men subfertility model using mice sperm incubated with OPRD1-agonist: reduced both fertilization rate and number of reached blastocysts [412] | ← |

***Genes***: *NPY2R, NPY4R,* and *NPY5R*, neuropeptide Y receptors Y2, Y4, and Y5, respectively; *NR5A1*, steroidogenic factor 1; *NRG1, NRG2, NRG3,* and *NRG4*, neuregulins 1, 2, 3, and 4, respectively; *NRP1* and *NRP2*, neuropilin 1 and 2, respectively; *NRTN*, neurturin; *NTF3* and *NTF4*, neurotrophins 3 and 4, respectively; *NTRK1, NTRK2,* and *NTRK3*, neurotrophic receptor tyrosine kinase 1, 2, and 3, respectively; *OGFR*, opioid growth factor receptor; *OPRD1*, opioid receptor δ1.

**Table S1.** Cont.

| # | Human Gene | Deficit (↓) | | | Excess (↑) | | |
|---|---|---|---|---|---|---|---|
| | | $N_{SNP}$ | Effect on reproductive potential [Reference] | ♂♀ | $N_{SNP}$ | Effect on reproductive potential [Reference] | ♂♀ |
| 215 | *OPRK1* | 1 [4] | within a model of human behavior using flocks of male starlings during the breeding season: improved sexual/agonistic aggressiveness [413] | → | 1 [4] | within a clinical cohort-based study: increased risks of breast cancer [414] | ← |
| 216 | *OPRL1* | 1 [4] | within a model of human behavior using mice: increased risks of fear-related behavior [415] | ← | 1 [4] | within a clinical cohort-based study: increased risks of pain hypersensitivity [416] | ← |
| 217 | *OPRM1* | 6 [4] | in human behavior models using Oprm1-knockout mice administered with morphine as OPRM1-agonist: pain hypersensitivity [417] | ← | 11 [4] | in human men subfertility model using mice sperm incubated with OPRD1-agonist: reduced both fertilization rate and number of reached blastocysts [418] | ← |
| 218 | *OSM* | | within a model of women fertility using pregnant mice with artificial injury in endometrium: reduced fertility [419] | ← | 1 [4] | in human disease models using mice with artificial spinal cord injury treated with exogenous OSM: improved post-injury recovery [417] | → |
| 219 | *OSMR* | 1 [4] | in traditional Chinese medicine, Fei-Yang-Chang-Wei-Yan capsule (FYC) reduces OSMR level to treat gastroenteritis and dysentery [420] | → | 6 [4] | worsened skin wound healing because of severe pruritus [421] | ← |
| 220 | *OXTR* | 1 [4] | within a model of human behavior using Oxtr-knockout mice: increased intermale aggressive behavior [422] | → | 2 [4] | in human maternal behavior models using lactating female rats versus nulliparous female mice: increased maternal aggression [423] | → |
| 221 | *PDGFA* | 1 [4] | in human disease models using mice with artificial atrial fibrillation treated with anti-PDGFA antibody: attenuated atrial fibrosis [424] | → | | in women fertility models using endometrial biopsies from women undergoing curettage for benign conditions: improved embryo implantation [425] | → |
| 222 | *PDGFB* | 2 [4] | within a clinical cohort-based ovarian ageing study: increased risks of diminished ovarian reserve [426] | ← | 6 [4] | within a model of human behavior using transgenic mice overexpressing Pdgfb: increased risks of locomotor dysfunction [427] | ← |
| 223 | *PDGFC* | 1 [4] | in women subfertility models using mice with artificial intrauterine adhesions injected with human amnion epithelial cells: increased pregnancy rate [428] | → | | within a clinical cohort-based surgical thyroid tissue biopsie study: increased risks of papillary thyroid carcinomas [429] | ← |
| 224 | *PDGFD* | 1 [4] | in human disease models using Pdgfd-knockout mice: increased risks of cardiac vasculature disorganization and arterial hypertension [430] | ← | 2 [4] | within a model of human diseases using transgenic mice overexpressing Pdgfd: improved skin wound healing [431] | → |
| 225 | *PDGFRA* | 1 [4] | increased risks of skeletal defects in newborns [432] | ← | 2 [4] | increased risks of fibrotic scar formation in infection and, thereby, infertility [433] | ← |
| 226 | *PDGFRB* | 1 [4] | within a model of human diseases using Pdgfrb-deficient mice: worsened post traumatic skin wound healing [434] | ← | 6 [4] | within a model of women fertility using female goats having whether two lambs or one lamb in offspring: improved fertility [435] | → |
| 227 | *PDGFRL* | | in renal cell carcinoma patients, lesser tumor mutation burden with lesser risks of worse prognosis, tumor metastasis and development [436] | → | 2 [4] | increased risks of hypertensive behavior and myocardial hypertrophy [437] | ← |
| 228 | *PDYN* | | in a model of human infertility using Zucker's fatty female rats, obese reduces Pdyn level compared to Zucker's lean female rats as a norm [438] | ← | 3 [4] | prevented behavior of conditioned fear [439] | → |
| 229 | *PENK* | 2 [4] | within a model of human behavior using Penk-knockout mice: increased risks of anxiety-like behavior in acute stress situations [440] | ← | 11 [4] | within a model of woman puberty using pre- and postpubertal female cattle: accelerated reproductive maturation [441] | → |
| 230 | *PGF* | 3 [4] | within a model of human development using Pgf-knockout mice treated with PGF-injections: increased risks of depression-like behavior [442] | ← | 7 [4] | within a model of human fertility using female buffaloes injected with PGF-analog: increased viable embryos rate [443] | → |
| 231 | *PGR* | 1 [3] | within a model of human diseases using PGR-knockout mice, infertility through embryo attachment impaired [444] | ← | 1 [3] | improved recidive-free survival after an estrogen receptor positive breast cancer recovery [445] | → |

***Genes***: *OPRK1* and *OPRM1*, opioid receptor κ1, and μ1, respectively; *OPRL1*, opioid related nociceptin receptor 1; *OSM* and *OSMR*, oncostatin M and its receptor, respectively; *OXTR*, oxytocin receptor; *PDGFA*, *PDGFB*, *PDGFC*, and *PDGFD*, platelet-derived growth factor subunits A, B, C, and D, respectively; *PDGFRA* and *PDGFRB*, platelet derived growth factor receptor α and β, respectively; *PDGFRL*, platelet derived growth factor receptor-like protein; *PDYN*, prodynorphin; *PENK*, proenkephalin; *PGF*, placental growth factor.; *PGR*, progesterone receptor;

**Table S1.** Cont.

| # | Human Gene | Deficit (↓) | | | Excess (↑) | | |
|---|---|---|---|---|---|---|---|
| | | $N_{SNP}$ | Effect on reproductive potential [Reference] | ♂♀ | $N_{SNP}$ | Effect on reproductive potential [Reference] | ♂♀ |
| 232 | *PLCXD1* | 15 [5] | increased risks of ischemic stroke and its complications in men of middle (reproductive) age [446] | ← | 35 [5] | within human cancer models using melanoma cells, transfection of a vector with PLCXD1 gene cDNA inhibits their proliferation [447] | → |
| 233 | *PNOC* | 1 [4] | within a model of human behavior using adult male mice with artificial pain induction: reduced pain sensitivity [448] | → | 3 [4] | within a model of men embryogenesis using mice embryos: improve fetal gubernaculum development and testis descent [449] | → |
| 234 | *POMC* | 4 [4] | within a model of human behavior using Pomc-deficient mice: increased acute aggressive response to social conflicts [450] | → | 1 [4] | in human behavior model using mice injected with viral construct carrying POMC: reduced both weight gain & adipose tissue reserves [451] | → |
| 235 | *PPP2R3B* | 3 [5] | within a model of human diseases using endemic for China carp fish *Gobiocypris rarus*, impaired spermatogenesis [452] | ← | 15 [5] | within human cancer models using melanoma cells, transfection of a plasmid with the PPP2R3B gene cDNA inhibits their growth [453] | → |
| 236 | *PROC* | 2 [3] | increased risks of life-threatening fulminant purpura in newborns [454] | ← | 6 [3] | within a model of human diseases using mice, increased risks of premature pregnancy loss [455] | ← |
| 237 | *PSPN* | | within a model of human diseases using Pspn-deficient mice: hypersensitivity to cerebral ischemia [456] | ← | 1 [4] | within a model of human behavior using organotypic spinal cord culture: reduced excitotoxic death of motor neurons in overload [457] | → |
| 238 | *P2RY8* | 2 [5] | increased risks of acute lymphoblastic leukemia in children [458] | ← | 2 [5] | increased risk of acute leukemia [459] | ← |
| 239 | *RET* | 3 [4] | in human disease models using Ret-knockout mice embryos: non-viability after birth via impaired development of the respiratory and nervous systems [460] | ← | 3 [4] | within a model of human diseases using transgenic male mice: increased risks of men sterility through spermatogenesis defects [461] | ← |
| 240 | *RPS4Y2* | 1 [5] | increased risks of male infertility [462] | ← | | increased risks of metabolic fatty liver diseases leading to liver cirrhosis and eventually cancer [463] | ← |
| 241 | *SHOX* | 5 [5] | increased risks of disproportionate short stature and Madelung's deformity as clubhand [464] | ← | 3 [5] | increased risks of pathoembryogenesis [465] | ← |
| 242 | *SLC6A3* | 1 [4] | within a model of human behavior using Slc6a3-knockout mice: increased risk-taking behavior [466] | ← | | in human behavior models using female mice & their male offspring under various diets: higher locomotion level regardless diets [467] | → |
| 243 | *SLC6A4* | 2 [4] | in models of human health using SLC6A4-knockout mice, improved both neuroplasticity and functioning of the small intestine [468] | → | 1 [4] | increased risks of depression, anxiety, spatial dullness, and cognitive inertia [469] | ← |
| 244 | *SLC25A6* | 1 [5] | increased risks of muscular dystrophy [470] | ← | 4 [5] | increased resistance to human herpesvirus type 5, which increases morbidity and mortality with weakened immunity [471] | → |
| 245 | *SNCA* | 5 [2] | within a model of human behavior using Snca-knockout mice: functional deficits in the nigrostriatal dopamine system [472] | ← | 3 [2] | within a model of human behavior using thransgenic mice overexpressing Snca: increased risk of motor deficits [473] | ← |
| 246 | *SOD1* | 1 [3] | decreased sperm motility and fertility *in vivo* [474] | ← | | increased both the bioavailability of copper in the germ cells and their protection against copper toxicity and oxidative stress [475] | → |
| 247 | *SPRY3* | | enhanced angiogenesis in tumors and cancer [476] | ← | 10 [5] | gender-specifically increased risks of autism among men compared to women [477] | ← |
| 248 | *STAR* | 1 [3] | increased risks of lipoid congenital adrenal hyperplasia [478] | ← | | increased risks of primary adrenal tumors [479] | ← |

***Genes***: *PLCXD1*, phosphatidylinositol-specific phospholipase C, X domain containing 1; *PNOC*, prepronociceptin; *POMC*, proopiomelanocortin; *PPP2R3B*, protein phosphatase 2 regulatory subunit β''β; *PROC*, protein C (synonym: inactivator of coagulation factors Va and VIIIa); *PSPN*, persephin; *P2RY8*, G-protein coupled purinergic P2Y receptor 8; *RET*, Ret proto-oncogene; *RPS4Y2*, ribosomal protein S4 Y-linked 2; *SHOX*, short stature homeobox; *SLC6A3* and *SLC6A4*, dopamine and serotonin transporters, respectively; *SLC25A6*, adenine nucleotide translocator 3; *SNCA*, synuclein α; *SOD1*, superoxide dismutase 1; *SPRY3*, sprouty RTK signaling antagonist 3; *STAR*, steroidogenic acute regulatory protein;.

**Table S1.** Cont.

| # | Human Gene | Deficit (↓) | | | Excess (↑) | | |
|---|---|---|---|---|---|---|---|
| | | $N_{SNP}$ | Effect on reproductive potential [Reference] | ♂♀ | $N_{SNP}$ | Effect on reproductive potential [Reference] | ♂♀ |
| 249 | *TAC1* | 1 [4] | within a model of human reproductive health using Tac1-knockout mice: delayed puberty onset [480] | ← | | within a cohort-based human brain tissue transcriptome study: behavioral dysfunction in depression [481] | ← |
| 250 | *TAC3* | | Tac3 receptor lack mice are subfertilite as a model of human diseases [482] | ← | 4 [4] | within a model of human fertility using female Damaraland mole-rats, antagonized to the socially induced infertility [483] | → |
| 251 | *TAC4* | 1 [4] | in human pain behavior models using rats injected with carrageenan: hyperalgesia and increased scratching post-injury behavior [484] | ← | 2 [4] | within a model of human pain using mice under artificial inflammatory orofacial pain: facial hyperallodynia [485] | ← |
| 252 | *TACR1* | | within a model of human pain using mice Tacr1-knockout mice: reduced liver fibrosis and biliary inflammation [486] | → | 1 [4] | within a model of human pain using mice administered with TACR1-agonists: advanced puberty onset [487] | → |
| 253 | *TACR2* | | within a model of human pain using mice Tacr2-knockout female mice: increased breeding intervals [488] | ← | 1 [4] | within a clinical cohort-based study: increased risks of recurrent major depressive disorder [489] | ← |
| 254 | *TBL1Y* | | increased risks of violations of both cardiogenesis and heart rate in men [490] | ← | 2 [5] | decreased risks of violations of both cardiogenesis and heart rate in men [490] | → |
| 255 | *TGFB1* | 2 [4] | in men subfertility models using busulfan-treated mice, which were next treated with small molecule TGFB1-inhibitor: repaired fertility [491] | → | | within a clinical cohort-based study: increased rsks of intervertebral disc degeneration [492] | ← |
| 256 | *TGFB2* | 1 [4] | within a model of human diseases using Tgfb2-knockout mice, increased risks of perinatal mortality [493] | ← | | impaired neuroregeneration through formation of post-traumatic collagen scar during wound healing [494] | ← |
| 257 | *TGFB3* | 1 [4] | within clinical study in assisted reproduction technologies, male infertility through reduced semen quality [495] | ← | 1 [4] | increased risks of infertility *via* uterine fibroids impairing decidualization [496] | ← |
| 258 | *TGFBI* | 4 [4] | within a model of human health using Tgfbi-deficient mice: suppressed tumor growth and metastasis [497] | → | 14 [4] | within a clinical cohort-based histopathological study: increased risks of oral squamous cell carcinoma [497] | ← |
| 259 | *TGFBR1* | 2 [4] | within a model of human diseases using Tgfbr1-knockout mice: severe aneurysmal degeneration of thoracic aortas [498] | ← | 4 [4] | in human disease models using primary human gingival fibroblasts ex vivo: improved neuroregeneration in oral tissue wound healing [499] | → |
| 260 | *TGFBR2* | 2 [4] | according to the ClinVar database, increased risks of thoracic aortic aneurysm and aortic dissection [500] | ← | | within a clinical cohort-based nasopharyngeal carcinoma tissue study: suppressed nasopharyngeal carcinoma progression [501] | → |
| 261 | *TGFBR3* | 2 [4] | within a model of human diseases using old male mice under maxillary molar tooth extractions: slowed oral cavity wound healing [502] | ← | 2 [4] | within a model of human diseases using rats with grafts: accelerated wound healing due to increased vascularization[504] | → |
| 262 | *TH* | 1 [2] | within a model of women reproductive health using female rats with small litters compared with those with normal litters: subfertility [505] | ← | | in human disease models using colitis rats treated with electroacupuncture: reduced pain hypersensitivity due to suppressed Th expression [506] | ← |
| 263 | *THBD* | 1 [3] | increased risks of placental insufficiency and fetal loss [507] | ← | | exogenous recombinant soluble human thrombomodulin is widely used as a drug against disseminated intravascular blood coagulation [508] | ← |
| 264 | *TMSB4Y* | | increased risks of prostate cancer [509] | ← | 1 [5] | gender-specific improved tumor suppression in men [510] | → |
| 265 | *TPH2* | | within a model of human newborn using Tph2-knockout mice: increased risks of mortality in childhood [511] | ← | 1 [4] | within a model of human behavior using transgenic mice: reduced aggressiveness [512] | ← |

***Genes***: *TAC1, TAC3, TAC4, TACR1,* and *TACR2*, tachykinin precursors 1, 3, 4, as well as receptors 1 and 2, respectively; *TBL1Y*, transducin β like 1 Y-linked; *TGFB1, TGFB2,* and *TGFB3*, transforming growth factors β1, β2, and β3, respectively; *TGFBI*, kerato-epithelin (synonym: as transforming growth factor β induced); *TGFBR1, TGFBR2,* and *TGFBR3*, transforming growth factor β receptor 1, 2, and 3, respectively; *TH*, tyrosine hydroxylase; *THBD*, thrombomodulin; *TMSB4Y*, thymosin β4 Y-linked; *TPH2,* tryptophan hydroxylase 2.

**Table S1.** Cont.

| # | Human Gene | Deficit (↓) | | | Excess (↑) | | |
|---|---|---|---|---|---|---|---|
| | | $N_{SNP}$ | Effect on reproductive potential [Reference] | ♂♀ | $N_{SNP}$ | Effect on reproductive potential [Reference] | ♂♀ |
| *266* | *TPI1* | 2 [3] | within a model of human diseases using mice, increased risks of asthenospermia [513] | ← | | increased risks of intrahepatic cholangiocarcinoma as the second most common primary tumor leading to liver cancer [514] | ← |
| *267* | *TSPY2* | 1 [5] | increased risks of male infertility [515] | ← | 2 [5] | increased risks of testicular maturation arrest [515] | ← |
| *268* | *TSPY4* | | increased risks of spermatogenesis disorders [516] | ← | 1 [5] | a synthetic agonist of gonadotropin-releasing hormone as a drug for male infertility increases TSPY4 level [516] | → |
| *269* | *USP9Y* | | within a model of human diseases using mice, reduced sperm quality [517] | ← | 1 [5] | increased risk of de novo heart failure in men [518] | ← |
| *270* | *UTY* | 1 [5] | within a model of human diseases using mice, increased risks of developmental defects in male embryos [519] | ← | | gender-specifically improve neurogenesis within the treatment of the nervous system in men [520] | → |
| *271* | *VAMP7* | 4 [5] | increased overall survival of patients with esophageal adenocarcinoma [521] | ← | 9 [5] | within a model of human diseases using mice, increased risks of subfertility [522] | ← |
| *272* | *VEGFA* | 4 [4] | within a clinical cohort-based study: antioxidant treatment with N-acetylcysteine protect ovarian follicles from ischemia-reperfusion injury due to reduced VEGFA expression [523] | → | 20 [4] | within a model of human diseases using rats with artificial wounds: improved skin wound healing[524] | → |
| *273* | *VEGFB* | | within a model of human embryogenesis using Vegfb-knockout mice: embryonic lethality [525] | ← | 2 [4] | within a model of human embryogenesis using mice under intracerebroventricular VEGFB administration: improved neurogenesis when brain injury causes central neuronal loss [526] | → |
| *274* | *ZBED1* | 1 [5] | increased risks of subfertility through adenovirus excess within spermatozoa in the later stages of infection [527] | ← | 11 [5] | increased risks of subfertility through adenovirus excess within spermatozoa in the early stages of infection [527] | ← |
| *275* | *ZFY* | | within a model of human diseases using bulls, reduced spermatozoa motility [528] | ← | 2 [5] | increased risks spermatocyte meiosis arrests leading to their apoptosis, azoospermia and, ultimately, infertility [529] | ← |

***Genes***: *TPI1*, triosephosphate isomerase 1; *TSPY2* and *TSPY4*, testis specific protein Y-linked 2 and 4, respectively; *USP9Y*, ubiquitin specific peptidase 9 Y-linked; *UTY*, ubiquitously transcribed tetratricopeptide repeat containing, histone demethylase UTY Y-linked; *VAMP7*, vesicle associated membrane protein 7 (synonym: synaptobrevin-like protein 1); *VEGFA* and *VEGFB*, vascular endothelial growth factor A and B, respectively; *ZBED1*, DNA replication-related element binding factor; *ZFY*, Zinc-finger protein Y-linked.

## References


1. Chadaeva, I., Ponomarenko, M., Rasskazov, D., Sharypova, E., Kashina, E., Matveeva, M., Arshinova, T., Ponomarenko, P., Arkova, O., Bondar, N. et al. Candidate SNP markers of aggressiveness-related complications and comorbidities of genetic diseases are predicted by a significant change in the affinity of TATA-binding protein for human gene promoters. *BMC Genomics*. **2016**; 17, 995.
2. Chadaeva, I., Rasskazov, D., Sharypova, E., Savinkova, L., Ponomarenko, P., Ponomarenko, M. Candidate SNP markers of social dominance, which may affect the affinity of the TATA-binding protein for human gene promoters. *Russ J Genet Appl Res*. **2017**; 7, 523-537.
3. Chadaeva, I., Ponomarenko, P., Rasskazov, D., Sharypova, E., Kashina, E., Zhechev, D., Drachkova, I., Arkova, O., Savinkova, L., Ponomarenko, M. et al. Candidate SNP markers of reproductive potential are predicted by a significant change in the affinity of TATA-binding protein for human gene promoters. *BMC Genomics*. **2018**; 19, 0.
4. Chadaeva, I., Ponomarenko, P., Rasskazov, D., Sharypova, E., Kashina, E., Kleshchev, M., Ponomarenko, M., Naumenko, V., Savinkova, L., Kolchanov N., et al. Natural selection equally supports the human tendencies in subordination and domination: a genome-wide study with in silico confirmation and in vivo validation in mice. *Front. Genet*. **2019**; 10, 73.

5. Ponomarenko, M., Kleshchev, M., Ponomarenko, P., Chadaeva, I., Sharypova, E., Rasskazov, D., Kolmykov, S., Drachkova, I., Vasiliev, G., Gutorova, N., *et al*. Disruptive natural selection by male reproductive potential prevents underexpression of protein-coding genes on the human Y chromosome as a self-domestication syndrome. *BMC Genetics*. **2020**; 21, 89.
6. Mogno, I., Vallania, F., Mitra, R.D., Cohen, B. TATA is a modular component of synthetic promoters. *Genome Res*. **2010**; 20, 1391-1397.
7. Ponomarenko, P., Suslov, V., Savinkova, L., Ponomarenko, M., Kolchanov, N. A precise equilibrium equation for four steps of binding between TBP and TATA-box allows for the prediction of phenotypical expression upon mutation. *Biofizika (Mosk)*. **2010**; 55, 358-369.
8. Velzing-Aarts, F.V., van der Dijs, F.P., Muskiet, F.A., Duits, A.J. The association of pre-eclampsia with the Duffy negative phenotype in women of West African descent. *BJOG*. **2002**; 109, 453-455.
9. Hernandez-Aguilera, A., Fibla, M., Cabre, N., Luciano-Mateo, F., Camps, J., Fernandez-Arroyo, S., Martin-Paredero, V., Menendez, J.A., Sirvent, J.J., Joven, J. Chemokine (C-C motif) ligand 2 and coronary artery disease: tissue expression of functional and atypical receptors. *Cytokine*. **2020**; 126, 154923.
10. Ross, R.A., Leon, S., Madara, J.C., Schafer, D., Fergani, C., Maguire, C.A., Verstegen, A.M., Brengle, E., Kong, D., Herbison, A.E., *et al*. PACAP neurons in the ventral premammillary nucleus regulate reproductive function in the female mouse. *Elife*. **2018**; 7, e35960.
11. Yamamoto, K., Hashimoto, H., Tomimoto, S., Shintani, N., Miyazaki, J., Tashiro, F., Aihara, H., Nammo, T., Li, M., Yamagata, K., *et al*. Overexpression of PACAP in transgenic mouse pancreatic beta-cells enhances insulin secretion and ameliorates streptozotocin-induced diabetes. *Diabetes*. 2003; 52, 1155-1162.
12. Jamen, F., Rodriguez-Henche, N., Pralong, F., Jegou, B., Gaillard, R., Bockaert, J., Brabet, P. PAC1 null females display decreased fertility. *Ann N Y Acad Sci*. **2000**; 921, 400-404.
13. Missig, G., Mei, L., Vizzard, M.A., Braas, K.M., Waschek, J.A., Ressler, K.J., Hammack, S.E., May, V. Parabrachial pituitary adenylate cyclase-activating polypeptide activation of amygdala endosomal extracellular signal-regulated kinase signaling regulates the emotional component of pain. *Biol Psychiatry*. **2017**; 81, 671-682.
14. Minelli, A., Liguori, L., Bellazza, I., Mannucci, R., Johansson, B., Fredholm, B.B. Involvement of A1 adenosine receptors in the acquisition of fertilizing capacity. *J Androl*. **2004**; 25, 286-292.
15. Minelli, A., Bellezza, I., Collodel, G., Fredholm, B.B. Promiscuous coupling and involvement of protein kinase C and extracellular signal-regulated kinase 1/2 in the adenosine A1 receptor signalling in mammalian spermatozoa. *Biochem Pharmacol*. 2008; 75, 931-941.
16. Azzinnari, D., Sigrist, H., Staehli, S., Palme, R., Hildebrandt, T., Leparc, G., Hengerer, B., Seifritz, E., Pryce, C.R. Mouse social stress induces increased fear conditioning, helplessness and fatigue to physical challenge together with markers of altered immune and dopamine function. *Neuropharmacology*. **2014**; 85, 328-341.
17. Sullivan, G.W., Fang, G., Linden, J., Scheld, W.M. A2A adenosine receptor activation improves survival in mouse models of endotoxemia and sepsis. *J Infect Dis*. **2004**; 189, 1897-1904.
18. Battistone, M.A., Merkulova, M., Park, Y.J., Peralta, M.A., Gombar, F., Brown, D., Breton, S. Unravelling purinergic regulation in the epididymis: activation of V-ATPase-dependent acidification by luminal ATP and adenosine. *J Physiol*. **2019**; 597, 1957-1973.
19. Budiono, B.P., See Hoe, L.E., Brunt, A.R., Peart, J.N., Headrick, J.P., Haseler, L.J. Coupling of myocardial stress resistance and signalling to voluntary activity and inactivity. *Acta Physiol (Oxf)*. **2016**; 218, 112-122.
20. Hofer, M., Pospisil, M., Dusek, L., Hoferova, Z., Weiterova, L., Komurkova, D. Erythropoiesis- and thrombopoiesis-characterizing parameters in adenosine A3 receptor knock-out mice. *Physiol Res*. **2013**; 62, 305-311.
21. Little, J.W., Ford, A., Symons-Liguori, A.M., Chen, Z., Janes, K., Doyle, T., Xie, J., Luongo, L., Tosh, D.K., Maione, S., *et al*. Endogenous adenosine A3 receptor activation selectively alleviates persistent pain states. *Brain*. **2015**; 138, 28-35.
22. Sanbe, A., Tanaka, Y., Fujiwara, Y., Tsumura, H., Yamauchi, J., Cotecchia, S., Koike, K., Tsujimoto, G., Tanoue, A. Alpha1-adrenoceptors are required for normal male sexual function. *Br J Pharmacol*. **2007**; 152, 332-340.
23. Doze, V.A., Handel, E.M., Jensen, K.A., Darsie, B., Luger, E.J., Haselton, J.R., Talbot, J.N., Rorabaugh, B.R. Alpha(1A)- and alpha(1B)-adrenergic receptors differentially modulate antidepressant-like behavior in the mouse. *Brain Res*. **2009**; 1285, 148-157.
24. Girven, M., Dugdale, H.F., Owens, D.J., Hughes, D.C., Stewart, C.E., Sharples, A.P. l-Glutamine improves skeletal muscle cell differentiation and prevents myotube atrophy after cytokine (TNF-$\alpha$) stress via reduced p38 MAPK signal transduction. *J Cell Physiol*. **2016**; 231, 2720-2732.
25. Sallinen, J., Haapalinna, A., Viitamaa, T., Kobilka, B.K., Scheinin, M. Adrenergic alpha2C-receptors modulate the acoustic startle reflex, prepulse inhibition, and aggression in mice. *J Neurosci*. **1998**; 18, 3035-3042.
26. Fernandois, D., Lara, H.E., Paredes, A.H. Blocking of β-adrenergic receptors during the subfertile period inhibits spontaneous ovarian cyst formation in rats. *Horm Metab Res*. **2012**; 44, 682-687.

27. Stemmelin, J., Cohen, C., Yalcin, I., Keane, P., Griebel, G. Implication of beta3-adrenoceptors in the antidepressant-like effects of amibegron using Adrb3 knockout mice in the chronic mild stress. *Behav Brain Res*. **2010**; 206, 310-312.
28. Claustre, Y., Leonetti, M., Santucci, V., Bougault, I., Desvignes, C., Rouquier, L., Aubin, N., Keane, P., Busch, S., Chen, Y., *et al*. Effects of the beta3-adrenoceptor (Adrb3) agonist SR58611A (amibegron) on serotonergic and noradrenergic transmission in the rodent: relevance to its antidepressant/anxiolytic-like profile. *Neuroscience*. **2008**; 156, 353-364.
29. Lee, B.P., Pilling, L.C., Bandinelli, S., Ferrucci, L., Melzer, D., Harries, L.W. The transcript expression levels of HNRNPM, HNRNPA0 and AKAP17A splicing factors may be predictively associated with ageing phenotypes in human peripheral blood. *Biogerontology*. **2019**; 20, 649-663.
30. Winge, S.B., Dalgaard, M.D., Jensen, J.M., Graem, N., Schierup, M.H., Juul, A., Rajpert-De, Meyts E., Almstrup, K. Transcriptome profiling of fetal Klinefelter testis tissue reveals a possible involvement of long non-coding RNAs in gonocyte maturation. *Hum Mol Genet*. **2018**; 27, 430-439.
31. Kimura, A., Hishimoto, A., Otsuka, I., Okazaki, S., Boku, S., Horai, T., Izumi, T., Takahashi, M., Ueno, Y., Shirakawa, O., Sora, I. Loss of chromosome Y in blood, but not in brain, of suicide completers. *PLoS One*. **2018**; 13, e0190667.
32. Chadwick, P., Acton, C. The use of amelogenin protein in the treatment of hard-to-heal wounds. *Br J Nurs*. **2009**; 18, S22, S24, S26, passim.
33. Peng, Y., Zhou, L., Cao, Y., Chen, P., Chen, Y., Zong, D., Ouyang, R. Relation between serum leptin levels, lipid profiles and neurocognitive deficits in Chinese OSAHS patients. *Int J Neurosci*. **2017**; 127, 981-987.
34. Manohar, M., Khan, H., Sirohi, V.K., Das, V., Agarwal, A., Pandey, A., Siddiqui, W.A., Dwivedi, A. Alteration in endometrial proteins during early- and mid-secretory phases of the cycle in women with unexplained infertility. *PLoS One*. **2014**; 9(11): e111687.
35. Harada, N., Minami, Y., Hanada, K., Hanaoka, R., Kobayashi, Y., Izawa, T., Sato, T., Kato, S., Inui, H., Yamaji, R. Relationship between gut environment, feces-to-food ratio, and androgen deficiency-induced metabolic disorders. *Gut Microbes*. **2020**; 12, 1817719.
36. Yang, Y.C., Fu, H.C., Wu, C.Y., Wei, K.T., Huang, K.E., Kang, H.Y. Androgen receptor accelerates premature senescence of human dermal papilla cells in association with DNA damage. *PLoS One*. **2013**; 8, e79434.
37. Honma, Y., Araki, T., Gianino, S., Bruce, A., Heuckeroth, R., Johnson, E., Milbrandt, J. Artemin is a vascular-derived neurotropic factor for developing sympathetic neurons. *Neuron*. **2002**; 35, 267-282.
38. Di Cesare Mannelli, L., Vivoli, E., Salvicchi, A., Schiavone, N., Koverech, A., Messano, M., Nicolai, R., Benatti, P., Bartolini, A., Ghelardini, C. Antidepressant-like effect of artemin in mice: a mechanism for acetyl-L-carnitine activity on depression. *Psychopharmacology (Berl)*. **2011**; 218, 347-356.
39. Wu, H.M., Zhao, C.C., Xie, Q.M., Xu, J., Fei, G.H. TLR2-melatonin feedback loop regulates the activation of NLRP3 inflammasome in murine allergic airway inflammation. *Front Immunol*. **2020**; 11, 172.
40. Gonzalez-Arto, M., Hamilton, T.R., Gallego, M., Gaspar-Torrubia, E., Aguilar, D., Serrano-Blesa, E., Abecia, J.A., Perez-Pe, R., Muino-Blanco, T., Cebrian-Perez, J.A., Casao, A. Evidence of melatonin synthesis in the ram reproductive tract. *Andrology*. **2016**; 4, 163-171.
41. Lau, Y.F., Zhang, J. Expression analysis of thirty one Y chromosome genes in human prostate cancer. *Mol Carcinog*. **2000**; 27, 308-321.
42. Firouzabadi, S.G., Kariminejad, R., Vameghi, R., Darvish, H., Ghaedi, H., Banihashemi, S., Firouzkouhi Moghaddam, M., Jamali, P., Mofidi Tehrani, H.F., Dehghani, H., *et al*. Copy number variants in patients with autism and additional clinical features: report of VIPR2 duplication and a novel microduplication syndrome. *Mol Neurobiol*. **2017**; 54, 7019-7027.
43. Tsuchiya, H., Fujimura, S., Fujiwara, Y., Koshimizu, T.A. Critical role of V1a vasopressin receptor in murine parturition. *Biol Reprod*. **2020**; 102, 923-934.
44. Wang, S.S., Kamphuis, W., Huitinga, I., Zhou, J.N., Swaab, D.F. Gene expression analysis in the human hypothalamus in depression by laser microdissection and real-time PCR: the presence of multiple receptor imbalances. *Mol Psychiatry*. **2008**; 13, 786-799, 741.
45. Sandgren, J.A., Deng, G., Linggonegoro, D.W., Scroggins, S.M., Perschbacher, K.J., Nair, A.R., Nishimura, T.E., Zhang, S.Y., Agbor, L.N., Wu, J., *et al*. Arginine vasopressin infusion is sufficient to model clinical features of preeclampsia in mice. *JCI Insight*. **2018**; 3, e99403.
46. Aydogan Kirmizi, D., Baser, E., Onat, T., Demir Caltekin, M., Yalvac, E.S., Kara, M., Gocmen, A.Y. Sexual function and depression in polycystic ovary syndrome: is it associated with inflammation and neuromodulators ? *Neuropeptides*. **2020**; 84, 102099.
47. Chow, R., Wessels, J.M., Foster, W.G. Brain-derived neurotrophic factor (BDNF) expression and function in the mammalian reproductive tract. *Hum Reprod Update*. **2020**; 26, 545-564.
48. Oaks, A.W., Zamarbide, M., Tambunan, D.E., Santini, E., Di Costanzo, S., Pond, H.L., Johnson, M.W., Lin, J., Gonzalez, D.M., Boehler, J.F., *et al*. Cc2d1a loss of function disrupts functional and morphological development in forebrain neurons leading to cognitive and social deficits. *Cereb Cortex*. **2017**; 27, 1670-1685.

49. Albert, P.R., Vahid-Ansari, F., Luckhart, C. Serotonin-prefrontal cortical circuitry in anxiety and depression phenotypes: pivotal role of pre- and post-synaptic 5-HT1A receptor expression. *Front Behav Neurosci*. **2014**; 8, 199.
50. Zamarbide, M., Oaks, A.W., Pond, H.L., Adelman, J.S., Manzini, M.C. Loss of the Intellectual Disability and Autism Gene Cc2d1a and Its Homolog Cc2d1b Differentially Affect Spatial Memory, Anxiety, and Hyperactivity. *Front Genet*. **2018**; 9, 65.
51. Kondaurova, E. M., Ilchibaeva, T. V., Tsybko, A. S., Kozhemyakina, R. V., Popova, N. K., & Naumenko, V. S. 5-HT1A receptor gene silencers Freud-1 and Freud-2 are differently expressed in the brain of rats with genetically determined high level of fear-induced aggression or its absence. *Behav Brain Res*. **2016**; 310, 20-25.
52. van Wanrooij, E.J., de Vos, P., Bixel, M.G., Vestweber, D., van Berkel, T.J., Kuiper J. Vaccination against CD99 inhibits atherogenesis in low-density lipoprotein receptor-deficient mice. *Cardiovasc Res*. **2008**; 78, 590-596.
53. Liu, J., Zhang, J., Ren, L., Wei, J., Zhu, Y., Duan, J., Jing, L., Sun, Z., Zhou, X. Fine particulate matters induce apoptosis via the ATM/P53/CDK2 and mitochondria apoptosis pathway triggered by oxidative stress in rat and GC-2spd cell. *Ecotoxicol Environ Saf*. **2019**; 180, 280-287.
54. Lindahl, M., Chalazonitis, A., Palm, E., Pakarinen, E., Danilova, T., Pham, T.D., Setlik, W., Rao, M., Voikar, V., Huotari, J., *et al*. Cerebral dopamine neurotrophic factor-deficiency leads to degeneration of enteric neurons and altered brain dopamine neuronal function in mice. *Neurobiol Dis*. **2020**; 134, 104696.
55. Lindholm, P., Voutilainen, M.H., Lauren, J., Peranen, J., Leppanen, V.M., Andressoo, J.O., Lindahl, M., Janhunen, S., Kalkkinen, N., Timmusk, T., *et al*. Novel neurotrophic factor CDNF protects and rescues midbrain dopamine neurons in vivo. *Nature*. **2007**; 448, 73-77.
56. Stahl, P.J., Mielnik, A.N., Barbieri, C.E., Schlegel, P.N., Paduch, D.A. Deletion or underexpression of the Y-chromosome genes CDY2 and HSFY is associated with maturation arrest in American men with nonobstructive azoospermia. *Asian J Androl*. **2012**; 14, 676-682.
57. Kleiman, S.E., Yogev, L., Hauser, R., Botchan, A., Bar-Shira, Maymon, B., Schreiber, L., Paz, G., Yavetz, H. Members of the CDY family have different expression patterns: CDY1 transcripts have the best correlation with complete spermatogenesis. *Hum Genet*. **2003**; 113, 486-492.
58. Plengpanich, W., Le Goff, W., Poolsuk, S., Julia, Z., Guerin, M., Khovidhunkit, W. CETP deficiency due to a novel mutation in the CETP gene promoter and its effect on cholesterol efflux and selective uptake into hepatocytes. *Atherosclerosis*. **2011**; 216, 370-373.
59. Silliman, K., Tall, A.R., Kretchmer, N., Forte, T.M. Unusual high-density lipoprotein subclass distribution during late pregnancy. *Metabolism*. **1993**; 42, 1592-1599.
60. Renz, B.W., Tanaka, T., Sunagawa, M., Takahashi, R., Jiang, Z., Macchini, M., Dantes, Z., Valenti, G., White, R.A., Middelhoff, M.A., *et al*. Cholinergic signaling via muscarinic receptors directly and indirectly suppresses pancreatic tumorigenesis and cancer stemness. *Cancer Discov*. **2018**; 8, 1458-1473.
61. Tanaka, S., Kuratsune, H., Hidaka, Y., Hakariya, Y., Tatsumi, K.I., Takano, T., Kanakura, Y., Amino, N. Autoantibodies against muscarinic cholinergic receptor in chronic fatigue syndrome. *Int J Mol Med*. **2003**; 12, 225-230.
62. Chauhan, P.S., Misra, U.K., Kalita, J., Chandravanshi, L.P., Khanna, V.K. Memory and learning seems to be related to cholinergic dysfunction in the JE rat model. *Physiol Behav*. **2016**; 156, 148-155.
63. Gibbons, A.S., Jeon, W.J., Scarr, E., Dean, B. Changes in muscarinic M2 receptor levels in the cortex of subjects with bipolar disorder and major depressive disorder and in rats after treatment with mood stabilisers and antidepressants. *Int J Neuropsychopharmacol*. **2016**; 19, pyv118.
64. Abiraman, K., Pol, S.U., O'Bara, M.A., Chen, G.D., Khaku, Z.M., Wang, J., Thorn, D., Vedia, B.H., Ekwegbalu, E.C., Li, J.X., *et al*. Anti-muscarinic adjunct therapy accelerates functional human oligodendrocyte repair. *J Neurosci*. **2015**; 35, 3676-3688.
65. Wang, N., Dong, B.J., Quan, Y., Chen, Q., Chu, M., Xu, J., Xue, W., Huang, Y.R., Yang, R., Gao, W.Q. Regulation of prostate development and benign prostatic hyperplasia by autocrine cholinergic signaling via maintaining the epithelial progenitor cells in proliferating status. *Stem Cell Reports*. **2016**; 6, 668-678.
66. Mulugeta, E., El-Bakri, N., Karlsson, E., Elhassan, A., Adem, A. Loss of muscarinic M4 receptors in spinal cord of arthritic rats: implications for a role of M4 receptors in pain response. *Brain Res*. 2003; 982, 284-287.
67. Chen, W.Y., Wen, Y.C., Lin, S.R., Yeh, H.L., Jiang, K.C., Chen, W.H., Lin, Y.S., Zhang, Q., Liew, P.L., Hsiao, M., *et al*. Nerve growth factor interacts with CHRM4 and promotes neuroendocrine differentiation of prostate cancer and castration resistance. *Commun Biol*. **2021**; 4, 22.
68. Erdogan, F., Ullmann, R., Chen, W., Schubert, M., Adolph, S., Hultschig, C., Kalscheuer, V., Ropers, H.H., Spaich, C., Tzschach, A. Characterization of a 5.3 Mb deletion in 15q14 by comparative genomic hybridization using a whole genome "tiling path" BAC array in a girl with heart defect, cleft palate, and developmental delay. *Am J Med Genet A*. **2007**;143A, 172-178.

69. Sakata, K., Overacre, A.E. Promoter IV-BDNF deficiency disturbs cholinergic gene expression of CHRNA5, CHRM2, and CHRM5: effects of drug and environmental treatments. *J Neurochem*. 2017; 143, 49-64.
70. Lefebvre, J.L., Ono, F., Puglielli, C., Seidner, G., Franzini-Armstrong, C., Brehm, P., Granato, M. Increased neuromuscular activity causes axonal defects and muscular degeneration. *Development*. **2004**; 131, 2605-2618.
71. Manzano, R., Toivonen, J.M., Olivan, S., Calvo, A.C., Moreno-Igoa, M., Munoz, M.J., Zaragoza, P., Garcia-Redondo, A., Osta, R. Altered expression of myogenic regulatory factors in the mouse model of amyotrophic lateral sclerosis. *Neurodegener Dis*. **2011**; 8, 386-396.
72. Mojica, C., Bai, Y., Lotfipour, S. Maternal nicotine exposure effects on adolescent learning and memory are abolished in alpha($\alpha$)2* nicotinic acetylcholine receptor-null mutant mice. *Neuropharmacology*. **2018**; 135, 529-535.
73. Aridon, P., Marini, C., Di Resta, C., Brilli, E., De Fusco, M., Politi, F., Parrini, E., Manfredi, I., Pisano, T., Pruna, D., *et al*. Increased sensitivity of the neuronal nicotinic receptor alpha 2 subunit causes familial epilepsy with nocturnal wandering and ictal fear. *Am J Hum Genet*. **2006**; 79, 342-350.
74. Louder, M.I.M., Hauber, M.E., Balakrishnan, C.N. Early social experience alters transcriptomic responses to species-specific song stimuli in female songbirds. *Behav Brain Res*. **2018**; 347, 69-76.
75. Grondahl, M.L., Borup, R., Vikesa, J., Ernst, E., Andersen, C.Y., Lykke-Hartmann, K. The dormant and the fully competent oocyte: comparing the transcriptome of human oocytes from primordial follicles and in metaphase II. *Mol Hum Reprod*. **2013**; 19, 600-617.
76. Ross, S.A., Wong, J.Y., Clifford, J.J., Kinsella, A., Massalas, J.S., Horne, M.K., Scheffer, I.E., Kola, I., Waddington, J.L., Berkovic, S.F., Drago, J. Phenotypic characterization of an alpha 4 neuronal nicotinic acetylcholine receptor subunit knock-out mouse. *J Neurosci*. **2000**; 20, 6431-6441.
77. Andersson, K.B., Florholmen, G., Winer, L.H., Tonnessen, T., Christensen, G. Regulation of neuronal type genes in congestive heart failure rats. *Acta Physiol (Oxf)*. **2006**; 186, 17-27.
78. Vincler, M.A., Eisenach, J.C. Knock down of the alpha 5 nicotinic acetylcholine receptor in spinal nerve-ligated rats alleviates mechanical allodynia. *Pharmacol Biochem Behav*. **2005**; 80, 135-143.
79. Mann, P.E. Gene expression profiling during pregnancy in rat brain tissue. *Brain Sci*. **2014**; 4, 125-135.
80. Tomas-Roig, J., Piscitelli, F., Gil, V., Quintana, E., Ramio-Torrenta, L.L., Del Rio, J.A., Moore, T.P., Agbemenyah, H., Salinas, G., Pommerenke, C., *et al*. Effects of repeated long-term psychosocial stress and acute cannabinoid exposure on mouse corticostriatal circuitries: Implications for neuropsychiatric disorders. *CNS Neurosci Ther*. **2018**;24, 528-538.
81. Rabenstein, R.L., Caldarone, B.J., Picciotto, M.R. The nicotinic antagonist mecamylamine has antidepressant-like effects in wild-type but not beta2- or alpha7-nicotinic acetylcholine receptor subunit knockout mice. *Psychopharmacology (Berl)*. **2006**; 189, 395-401.
82. Uteshev, V.V. $\alpha$7 Nicotinic ACh receptors as a ligand-gated source of Ca(2+) ions: the search for a Ca(2+) optimum. *Adv Exp Med Biol*. **2012**; 740, 603-638.
83. Mohammadi, S.A., Christie, M.J. Conotoxin interactions with $\alpha$9$\alpha$10-nAChRs: is the $\alpha$9$\alpha$10-nicotinic acetylcholine receptor an important therapeutic target for pain management ? *Toxins (Basel)*. **2015**; 7, 3916-3932.
84. Vastrad, B., Vastrad, C., Godavarthi, A., Chandrashekar, R. Molecular mechanisms underlying gliomas and glioblastoma pathogenesis revealed by bioinformatics analysis of microarray data. *Med Oncol*. **2017**; 34, 182.
85. Vincler, M., Wittenauer, S., Parker, R., Ellison, M., Olivera, B.M., McIntosh, JM. Molecular mechanism for analgesia involving specific antagonism of alpha9alpha10 nicotinic acetylcholine receptors. *Proc Natl Acad Sci USA*. **2006**; 103, 17880-17884.
86. Hecker, A., Kullmar, M., Wilker, S., Richter, K., Zakrzewicz, A., Atanasova, S., Mathes, V., Timm, T., Lerner, S., Klein, J., *et al*. Phosphocholine-modified macromolecules and canonical nicotinic agonists inhibit ATP-induced IL-1β release. *J Immunol*. **2015**; 195, 2325-2334.
87. Agerholm, J.S., McEvoy, F.J., Menzi, F., Jagannathan, V., Drogemuller, C. A CHRNB1 frameshift mutation is associated with familial arthrogryposis multiplex congenita in Red dairy cattle. *BMC Genomics*. **2016**; 17, 479.
88. Burniston, J.G., Meek, T.H., Pandey, S.N., Broitman-Maduro, G., Maduro, M.F., Bronikowski, A.M., Garland, T. Jr., Chen, Y.W. Gene expression profiling of gastrocnemius of "minimuscle" mice. *Physiol Genomics*. **2013**; 45, 228-236.
89. Han, S., Yang, S.H., Kim, J.Y., Mo, S., Yang, E., Song, K.M., Ham, B.J., Mechawar, N., Turecki, G., Lee, H.W., Kim, H. Down-regulation of cholinergic signaling in the habenula induces anhedonia-like behavior. *Sci Rep*. **2017**; 7, 900.
90. Kamens, H.M., Miyamoto, J., Powers, M.S., Ro, K., Soto, M., Cox, R., Stitzel, J.A., Ehringer, M.A. The β3 subunit of the nicotinic acetylcholine receptor: modulation of gene expression and nicotine consumption. *Neuropharmacology*. **2015**; 99: 639-649.

91. Vinals, X., Molas, S., Gallego, X., Fernandez-Montes, R.D., Robledo, P., Dierssen, M., Maldonado, R. Overexpression of α3/α5/β4 nicotinic receptor subunits modifies impulsive-like behavior. *Drug Alcohol Depend*. **2012**; 122, 247-252.
92. Webster, R., Maxwell, S., Spearman, H., Tai, K., Beckstein, O., Sansom, M., Beeson, D. A novel congenital myasthenic syndrome due to decreased acetylcholine receptor ion-channel conductance. *Brain*. **2012**; 135, 1070-80.
93. Maclennan, C.A., Vincent, A., Marx, A., Willcox, N., Gilhus, N.E., Newsom-Davis, J., Beeson, D. Preferential expression of AChR epsilon-subunit in thymomas from patients with myasthenia gravis. *J Neuroimmunol*. **2008**; 201-202, 28-32.
94. Hoffmann, K., Muller, J.S., Stricker, S., Megarbane, A., Rajab, A., Lindner, T.H., Cohen, M., Chouery, E., Adaimy, L., Ghanem, I., *et al*. Escobar syndrome is a prenatal myasthenia caused by disruption of the acetylcholine receptor fetal gamma subunit. *Am J Hum Genet*. **2006**; 79, 303-312.
95. Chang, B., Li, F., Pang, L.J., Hu, W.H., Lu, T.C., Li, H.A., Wang, J., Zhu, X.Z., Shi, D.R. Study on the diagnostic significance of detecting the expression of AChR-gamma mRNA in rhabdomyosarcoma tissues. *Zhonghua Bing Li Xue Za Zhi (Chinese Journal of Pathology)*. **2003**; 32, 413-416.
96. Bowers, M.E., Ressler, K.J. Sex-dependence of anxiety-like behavior in cannabinoid receptor 1 (Cnr1) knockout mice. *Behav Brain Res*. **2016**; 300, 65-69.
97. Schneider, M., Kasanetz, F., Lynch, D.L., Friemel, C.M., Lassalle, O., Hurst, D.P., Steindel, F., Monory, K., Schafer, C., Miederer, I., *et al*. Enhanced functional activity of the cannabinoid type-1 receptor mediates adolescent behavior. *J Neurosci*. **2015**; 35, 13975-13988.
98. Peruga, I., Hartwig, S., Merkler, D., Thone, J., Hovemann, B., Juckel, G., Gold, R., Linker, R.A. Endogenous ciliary neurotrophic factor modulates anxiety and depressive-like behavior. *Behav Brain Res*. **2012**; 229, 325-332.
99. Dulz, S., Bassal, M., Flachsbarth, K., Riecken, K., Fehse, B., Schlichting, S., Bartsch, S., Bartsch, U. Intravitreal co-administration of GDNF and CNTF confers synergistic and long-lasting protection against injury-induced cell death of retinal ganglion cells in mice. Cells. **2020**; 9, 2082.
100. Wilhelm, C.J., Choi, D., Huckans, M., Manthe, L., Loftis, J.M. Adipocytokine signaling is altered in Flinders sensitive line rats, and adiponectin correlates in humans with some symptoms of depression. *Pharmacol Biochem Behav*. **2013**; 103, 643-651.
101. Desbonnet, L., Tighe, O., Karayiorgou, M., Gogos, J.A., Waddington, J.L., O'Tuathaigh, C.M. Physiological and behavioural responsivity to stress and anxiogenic stimuli in COMT-deficient mice. *Behav Brain Res*. **2012**; 228, 351-358.
102. Lay, M.K., Cespedes, P.F., Palavecino, C.E., Leon, M.A., Diaz, R.A., Salazar, F.J., Mendez, G.P., Bueno, S.M., Kalergis, A.M. Human metapneumovirus infection activates the TSLP pathway that drives excessive pulmonary inflammation and viral replication in mice. *Eur J Immunol*. **2015**; 45, 1680-1695.
103. Dou, H., Chen, X., Huang, Y., Su, Y., Lu, L., Yu, J., Yin, Y., Bao, L. Prognostic significance of P2RY8-CRLF2 and CRLF2 overexpression may vary across risk subgroups of childhood B-cell acute lymphoblastic leukemia. *Genes Chromosomes Cancer*. **2017**; 56, 135-146.
104. Arumugam, P., Suzuki, T., Shima, K., McCarthy, C., Sallese, A., Wessendarp, M., Ma, Y., Meyer, J., Black, D., Chalk, C., *et al*. Long-term safety and efficacy of gene-pulmonary macrophage transplantation therapy of PAP in Csf2ra-/- mice. *Mol Ther*. **2019**; 27, 1597-1611.
105. Huang, G.J., Edwards, A., Tsai, C.Y., Lee, Y.S., Peng, L., Era, T., Hirabayashi, Y., Tsai, C.Y., Nishikawa, S., Iwakura, Y., *et al*. Ectopic cerebellar cell migration causes maldevelopment of Purkinje cells and abnormal motor behaviour in Cxcr4 null mice. *PLoS One*. **2014**; 9, e86471.
106. Yang, F., Sun, W., Yang, Y., Wang, Y., Li, C.L., Fu, H., Wang, X.L., Yang, F., He, T., Chen, J. SDF1-CXCR4 signaling contributes to persistent pain and hypersensitivity via regulating excitability of primary nociceptive neurons: involvement of ERK-dependent Nav1.8 up-regulation. *J Neuroinflammation*. **2015**; 12, 219.
107. Xie, C., Wen, X., Ding, P., Liu, T., He, Y., Niu, Z., Lin, J., Yuan, S., Guo, X., Jia, D., Chen W. Influence of CYP2A6*4 genotypes on maternal serum cotinine among Chinese nonsmoking pregnant women. *Nicotine Tob Res*. **2014**; 16, 406-412.
108. Lee, D., Seo, S.B., Lee, H.J., Park, T.S., Shim, S.M. Using phytochemicals to investigate the activation of nicotine detoxification via upregulation of CYP2A6 in animal models exposed tobacco smoke condensate by intratracheal instillation. *Evid Based Complement Alternat Med*. **2018**; 2018, 7635197.
109. Yan, H., Li, Z., Shen, Q., Wang, Q., Tian, J., Jiang, Q., Gao, L. Aberrant expression of cell cycle and material metabolism related genes contributes to hepatocellular carcinoma occurrence. *Pathol Res Pract*. **2017**; 213, 316-321.
110. Yoshioka, M., Takenouchi, T., Kitani, H., Guruge, K.S., Yamanaka, N. Synergistic induction of drug-metabolizing enzymes in co-cultures of bovine hepatocytic and sinusoidal cell lines. *In Vitro Cell Dev Biol Anim*. **2020**; 56, 2-9.
111. Marsh, C.A., Auchus, R.J. Fertility in patients with genetic deficiencies of cytochrome P450c17 (CYP17A1): combined 17-hydroxylase/17,20-lyase deficiency and isolated 17,20-lyase deficiency. *Fertil Steril*. **2014**; 101, 317-322.

112. Nna, V.U., Bakar, A.B.A., Ahmad, A., Umar, U.Z., Suleiman, J.B., Zakaria, Z., Othman, Z.A., Mohamed, M. Malaysian propolis and metformin mitigate subfertility in streptozotocin-induced diabetic male rats by targeting steroidogenesis, testicular lactate transport, spermatogenesis and mating behaviour. *Andrology*. **2020**; 8, 731-746.
113. Hajian, B., Scocchera, E., Shoen, C., Krucinska, J., Viswanathan, K., G-Dayanandan, N., Erlandsen, H., Estrada, A., Mikusova, K., Kordulakova, J., *et al*. Drugging the folate pathway in mycobacterium tuberculosis: the role of multi-targeting agents. *Cell Chem Biol*. **2019**; 26, 781-791.e6.
114. Skubisz, M.M., Tong, S. Of leaves and butterflies: how methotrexate came to be the savior of women. *Obstet Gynecol*. **2011**; 118, 1169-1173.
115. Zhang, G., Luo, Y., Li, G., Wang, L., Na, D., Wu, X., Zhang, Y., Mo, X., Wang, L. DHRSX, a novel non-classical secretory protein associated with starvation induced autophagy. *Int J Med Sci*. **2014**; 11, 962-970.
116. Tian, Y., Stamova, B., Jickling, G.C., Xu, H., Liu, D., Ander, B.P., Bushnell, C., Zhan, X., Turner, R.J., Davis, R.R., *et al*. Y chromosome gene expression in the blood of male patients with ischemic stroke compared with male controls. *Gend Med*. **2012**; 9, 68-75.e3.
117. Awada, H., Mahfouz, R.Z., Kishtagari, A., Kuzmanovic, T., Durrani, J., Kerr, C.M., Patel, B.J., Visconte, V., Radivoyevitch, T., Lichtin, A., *et al*. Extended experience with a non-cytotoxic DNMT1-targeting regimen of decitabine to treat myeloid malignancies. *Br J Haematol*. **2020**; 188, 924-929.
118. Matrisciano, F,. Tueting, P., Dalal, I., Kadriu, B., Grayson, D.R., Davis, J.M., Nicoletti, F., Guidotti, A. Epigenetic modifications of GABAergic interneurons are associated with the schizophrenia-like phenotype induced by prenatal stress in mice. *Neuropharmacology*. **2013**; 68, 184-194.
119. Chou, L.Y., Chen, C.H., Chuang, S.C., Cheng, T.L., Lin, Y.H., Chou, H.C., Fu, Y.C., Wang, Y.H., Wang, C.Z. Discoidin domain receptor 1 regulates Runx2 during Osteogenesis of osteoblasts and promotes bone ossification via phosphorylation of p38. *Int J Mol Sci*. **2020**; 21, 7210.
120. Freund, N., Thompson, B.S., Sonntag, K., Meda, S., Andersen, S.L. When the party is over: depressive-like states in rats following termination of cortical D1 receptor overexpression. *Psychopharmacology (Berl)*. **2016**; 233, 1191-1201.
121. Graham, D.L., Durai, H.H., Garden, J.D., Cohen, E.L., Echevarria, F.D., Stanwood, G.D. Loss of dopamine D2 receptors increases parvalbumin-positive interneurons in the anterior cingulate cortex. *ACS Chem Neurosci*. **2015**; 6, 297-305.
122. Chen, C., Yang, J.M., Hu, T.T., Xu, T.J., Xu, W.P., Wei, W. Elevated dopamine D2 receptor in prefrontal cortex of CUMS rats is associated with downregulated cAMP-independent signaling pathway. *Can J Physiol Pharmacol*. **2013**; 91, 750-758.
123. Seo, J.H., Kuzhikandathil, E.V. Dopamine D3 receptor mediates preadolescent stress-induced adult psychiatric disorders. *PLoS One*. **2015**; 10, e0143908.
124. Ikeda, E., Matsunaga, N., Kakimoto, K., Hamamura, K., Hayashi, A., Koyanagi, S., Ohdo, S. Molecular mechanism regulating 24-hour rhythm of dopamine D3 receptor expression in mouse ventral striatum. *Mol Pharmacol*. 2013; 83, 959-967.
125. Keck, T.M., Suchland, K.L., Jimenez, C.C., Grandy, D.K. Dopamine D4 receptor deficiency in mice alters behavioral responses to anxiogenic stimuli and the psychostimulant methylphenidate. *Pharmacol Biochem Behav*. **2013**; 103, 831-841.
126. Azadmarzabadi, E., Haghighatfard, A., Mohammadi, A. Low resilience to stress is associated with candidate gene expression alterations in the dopaminergic signalling pathway. *Psychogeriatrics*. **2018**; 18, 190-201.
127. Inoue, T., Izumi, T., Maki, Y., Muraki, I., Koyama, T. Effect of the dopamine D(1/5) antagonist SCH 23390 on the acquisition of conditioned fear. *Pharmacol Biochem Behav*. **2000**; 66, 573-578.
128. Schneider, T., Ilott, N., Brolese, G., Bizarro, L., Asherson, P.J., Stolerman, I.P. Prenatal exposure to nicotine impairs performance of the 5-choice serial reaction time task in adult rats. *Neuropsychopharmacology*. **2011**; 36, 1114-1125.
129. Ivanski, F., de Oliveira, V.M., de Oliveira, I.M., de Araujo Ramos, A.T., de Oliveira, Tonete, S.T., de Oliveira Hykavei, G., Bargi-Souza, P., Schiessel, D.L., Martino-Andrade, A.J., *et al*. Prepubertal acrylamide exposure causes dose-response decreases in spermatic production and functionality with modulation of genes involved in the spermatogenesis in rats. *Toxicology*. **2020**; 436, 152428.
130. Bai, X., Hippensteel, J., Leavitt, A., Maloney, J.P., Beckham, D., Garcia, C., Li, Q., Freed, B.M., Ordway, D., Sandhaus, R.A., Chan, E.D. Hypothesis: alpha-1-antitrypsin is a promising treatment option for COVID-19. *Med Hypotheses*. **2020**; 110394.
131. Huang, Q.T., Chen, J.H., Hang, L.L., Liu, S.S., Zhong, M. Activation of PAR-1/NADPH oxidase/ROS signaling pathways is crucial for the thrombin-induced sFlt-1 production in extravillous trophoblasts: possible involvement in the pathogenesis of preeclampsia. *Cell Physiol Biochem*. **2015**; 35, 1654-1662.
132. Yu, Q., Yang, X., Zhang, C., Zhang, X., Wang, C., Chen, L., Liu, X., Gu, Y., He, X., Hu, L., *et al*. AMPK activation by ozone therapy inhibits tissue factor-triggered intestinal ischemia and ameliorates chemotherapeutic enteritis. *FASEB J*. **2020**; 34, 13005-13021.

133. Arnaud, E., Barbalat, V., Nicaud, V., Cambien, F., Evans, A., Morrison, C., Arveiler, D., Luc, G., Ruidavets, J.B., Emmerich, J., *et al*. Polymorphisms in the 5' regulatory region of the tissue factor gene and the risk of myocardial infarction and venous thromboembolism: the ECTIM and PATHROS studies. Etude Cas-Temoins de l'Infarctus du Myocarde. Paris thrombosis case-control study. *Arterioscler Thromb Vasc Biol*. **2000**; 20, 892-898.
134. Senol, B.K., Zulfikar, B. Clinical problems and surgical interventions in inherited factor VII deficiency. *Turk Pediatri Ars*. **2020**; 55, 184-190.
135. Burad, J., Bhakta, P., Sharma, J.. Timely 'off-label' use of recombinant activated factor VII (NovoSeven(®)) can help in avoiding hysterectomy in intractable obstetric bleeding complicated with disseminated intravascular coagulation: A case report and review of the literature. *Indian J Anaesth*. **2012**; 56, 69-71.
136. Gonzalez-Ramos, I.A., Mantilla-Capacho, J.M., Luna-Zaizar, H., Mundo-Ayala, J.N., Lara-Navarro, I.J., Ornelas-Ricardo, D., Gonzalez Alcazar, J.A., Evangelista-Castro, N., Jaloma-Cruz, A.R. Genetic analysis for carrier diagnosis in hemophilia A and B in the Mexican population: 25 years of experience. *Am J Med Genet C Semin Med Genet*. 2020; doi: 10.1002/ajmg.c.31854
137. Schambeck, C.M., Grossmann, R., Zonnur, S., Berger, M., Teuchert, K., Spahn, A., Walter, U. High factor VIII (FVIII) levels in venous thromboembolism: role of unbound FVIII. *Thromb Haemost*. **2004**; 92, 42-46.
138. Lin, H.F., Maeda, N., Smithies, O., Straight, D.L., Stafford, D.W. A coagulation factor IX-deficient mouse model for human hemophilia B. *Blood*. **1997**; 90, 3962-3966.
139. Ameri, A., Kurachi, S., Sueishi, K., Kuwahara, M., Kurachi, K. Myocardial fibrosis in mice with overexpression of human blood coagulation factor IX. *Blood*. **2003**; 101, 1871-1873.
140. Khealani, B., Farhat, Z., Mozaffar, T. Factor XI deficiency-related spontaneous primary intraventricular hemorrhage. *South Med J*. **2000**; 93, 1017-1018.
141. Witzke, G., Bork, K., Benes, P., Bockers, M. Hereditary angioneurotic oedema and blood-coagulation: interaction between C1-esterase-inhibitor and the activation factors of the proteolytic enzyme systems. *Klin Wochenschr*. **1983**; 61, 1131-1135.
142. Peplow, P.V., Baxter, G.D. Gene expression and release of growth factors during delayed wound healing: a review of studies in diabetic animals and possible combined laser phototherapy and growth factor treatment to enhance healing. *Photomed Laser Surg*. **2012**; 30, 617-636.
143. Sun, L., Xu, L., Chang, H., Henry, F.A., Miller, R.M., Harmon, J.M., Nielsen, T.B. Transfection with aFGF cDNA improves wound healing. *J Invest Dermatol*. 1997; 108, 313-318.
144. Goldshmit, Y., Sztal, T.E., Jusuf, P.R., Hall, T.E., Nguyen-Chi, M., Currie, P.D. Fgf-dependent glial cell bridges facilitate spinal cord regeneration in zebrafish. *J Neurosci*. **2012**; 32, 7477-7492.
145. Zhang, C., Yang, T., Jiang, H. miR-511 inhibits proliferation and metastasis of breast cancer cells by targeting FGF4. *J Gene Med*. **2020**; 22, e3168.
146. Srivastava, A.S., Shenouda, S., Mishra, R., Carrier, E. Transplanted embryonic stem cells successfully survive, proliferate, and migrate to damaged regions of the mouse brain. Stem Cells. **2006**; 24, 1689-1694.
147. Zhu, Z., Wu, Q., Zhang, M., Tong, J., Zhong, B., Yuan, K. Hsa_circ_0016760 exacerbates the malignant development of non-small cell lung cancer by sponging miR-145-5p/FGF5. *Oncol Rep*. **2020**; 45, 501–512.
148. Sautter, N.B., Delaney, K.L., Hausman, F.A., Trune, D.R. Tissue remodeling gene expression in a murine model of chronic rhinosinusitis. *Laryngoscope*. **2012**; 122, 711-717.
149. Brooks L.R., Pals, H.L., Enix, C.L., Woolaver, R.A., Paul, E.D., Lowry, C.A., Tsai, P.S. Fibroblast growth factor 8 deficiency compromises the functional response of the serotonergic system to stress. *PLoS One*. **2014**; 9, e101420.
150. Daphna-Iken, D., Shankar, D.B., Lawshe, A., Ornitz, D.M., Shackleford, G.M., MacArthur, C.A. MMTV-Fgf8 transgenic mice develop mammary and salivary gland neoplasia and ovarian stromal hyperplasia. *Oncogene*. **1998**; 17, 2711-2717.
151. Komi-Kuramochi, A., Kawano, M., Oda, Y., Asada, M., Suzuki, M., Oki, J., Imamura, T. Expression of fibroblast growth factors and their receptors during full-thickness skin wound healing in young and aged mice. *J Endocrinol*. **2005**; 186, 273-289.
152. Yang, F., Whelan, E.C., Guan, X., Deng, B., Wang, S., Sun, J., Avarbock, M.R., Wu, X., Brinster, R.L. FGF9 promotes mouse spermatogonial stem cell proliferation mediated by p38 MAPK signalling. *Cell Prolif*. **2021**; 54, e12933.
153. Teague, W.J., Jones, M.L.M., Hawkey, L., Smyth, I.M., Catubig, A., King, S.K., Sarila, G., Li, R., Hutson, J.M. FGF10 and the mystery of duodenal atresia in humans. *Front Genet*. **2018**; 9, 530.
154. Tejedor, G., Laplace-Builhe, B., Luz-Crawford, P., Assou, S., Barthelaix, A., Mathieu, M., Kissa, K., Jorgensen, C., Collignon, J., Chuchana, P., Djouad, F. Whole embryo culture, transcriptomics and RNA interference identify TBX1 and FGF11 as novel regulators of limb development in the mouse. *Sci Rep*. 2020; 10, 3597.
155. Lin, H.Y., Peng, C.W., Wu, W.W. Fibrous hydrogel scaffolds with cells embedded in the fibers as a potential tissue scaffold for skin repair. *J Mater Sci Mater Med*. **2014**; 25, 259-269.
156. McCreary, J.K., Truica, L.S., Friesen, B., Yao, Y., Olson, D.M., Kovalchuk, I., Cross, A.R., Metz, G.A. Altered brain morphology and functional connectivity reflect a vulnerable affective state after cumulative multigenerational stress in rats. *Neuroscience*. **2016**; 330, 79-89.

157. Merhi, Z., Polotsky, A.J., Bradford, A.P., Buyuk, E., Chosich, J., Phang, T., Jindal, S., Santoro, N. Adiposity alters genes important in inflammation and cell cycle division in human cumulus granulosa cell. *Reprod Sci*. 2015; 22, 1220-1228.
158. Sinden, D.S., Holman, C.D., Bare, C.J., Sun, X., Gade, A.R., Cohen, D.E., Pitt, G.S. Knockout of the X-linked Fgf13 in the hypothalamic paraventricular nucleus impairs sympathetic output to brown fat and causes obesity. *FASEB J*. **2019**; 33, 11579-11594.
159. Li, J., Wang, Q., Wang, H., Wu, Y., Yin, J., Chen, J., Zheng, Z., Jiang, T., Xie, L., Wu, F., *et al*. Lentivirus mediating FGF13 enhances axon regeneration after spinal cord injury by stabilizing microtubule and improving mitochondrial function. *J Neurotrauma*. **2018**; 35, 548-559.
160. Bosch, M.K., Carrasquillo, Y., Ransdell, J.L., Kanakamedala, A., Ornitz, D.M., Nerbonne, J.M. Intracellular FGF14 (iFGF14) is required for spontaneous and evoked firing in cerebellar purkinje neurons and for motor coordination and balance. *J Neurosci*. **2015**; 35, 6752-6769.
170. Turkowski, K., Herzberg, F., Gunther, S., Brunn, D., Weigert, A., Meister, M., Muley, T., Kriegsmann, M., Schneider, M.A., Winter, H., et al. Fibroblast growth factor-14 acts as tumor suppressor in lung adenocarcinomas. *Cells*. **2020**; 9, 1755.
171. Scearce-Levie, K., Roberson, E.D., Gerstein, H., Cholfin, J.A., Mandiyan, V.S., Shah, N.M., Rubenstein, J.L., Mucke, L. Abnormal social behaviors in mice lacking Fgf17. *Genes Brain Behav*. **2008**; 7, 344-354.
172. Heer, R., Douglas, D., Mathers, M.E., Robson, C.N., Leung, H.Y. Fibroblast growth factor 17 is overexpressed in human prostate cancer. *J Pathol*. **2004**; 204, 578-586.
173. Hagan, A.S., Boylan, M., Smith, C., Perez-Santamarina, E., Kowalska, K., Hung, I.H., Lewis, R.M., Hajihosseini, M.K., Lewandoski, M., Ornitz, D.M. Generation and validation of novel conditional flox and inducible Cre alleles targeting fibroblast growth factor 18 (Fgf18). *Dev Dyn*. **2019**; 248, 882-893.
174. Ellsworth, J.L., Garcia, R., Yu, J., Kindy, M.S. Fibroblast growth factor-18 reduced infarct volumes and behavioral deficits after transient occlusion of the middle cerebral artery in rats. *Stroke*. **2003**; 34, 1507-1512.
175. Hao, Y., Zhou, J., Zhou, M., Ma, X., Lu, Z., Gao, M., Pan, X., Tang, J., Bao, Y., Jia, W. Serum levels of fibroblast growth factor 19 are inversely associated with coronary artery disease in chinese individuals. *PLoS One*. **2013**; 8, e72345.
176. Ovadia, C., Perdones-Montero, A., Fan, H.M., Mullish, B.H., McDonald, J.A.K., Papacleovoulou, G., Wahlstrom, A., Stahlman, M., Tsakmaki, A., Clarke, L.C.D., *et al*. Ursodeoxycholic acid enriches intestinal bile salt hydrolase-expressing Bacteroidetes in cholestatic pregnancy. *Sci Rep*. **2020**; 10, 3895.
177. Elo, T., Lindfors, P.H., Lan, Q., Voutilainen, M., Trela, E., Ohlsson, C., Huh, S.H., Ornitz, D.M., Poutanen, M., Howard, B.A., Mikkola, M.L. Ectodysplasin target gene Fgf20 regulates mammary bud growth and ductal invasion and branching during puberty. *Sci Rep*. **2017**; 7, 5049.
178. Sleeman, I.J., Boshoff, E.L., Duty, S. Fibroblast growth factor-20 protects against dopamine neuron loss in vitro and provides functional protection in the 6-hydroxydopamine-lesioned rat model of Parkinson's disease. *Neuropharmacology*. **2012**; 63, 1268-1277.
179. Oost, L.J., Kustermann, M., Armani, A., Blaauw, B., Romanello, V. Fibroblast growth factor 21 controls mitophagy and muscle mass. *J Cachexia Sarcopenia Muscle*. **2019**; 10, 630-642.
180. Chang, H.H., Chen, P.S., Cheng, Y.W., Wang, T.Y., Yang, Y.K., Lu, R.B. FGF21 is associated with metabolic effects and treatment response in depressed bipolar ii disorder patients treated with valproate. *Int J Neuropsychopharmacol*. **2018**; 21, 319-324.
181. Xu, Y.H., Yu, M., Wei, H., Yao, S., Chen, S.Y., Zhu, X.L., Li, Y.F. Fibroblast growth factor 22 is a novel modulator of depression through interleukin-1β. *CNS Neurosci Ther*. **2017**; 23, 907-916.
182. Yamaguchi ,T.P., Harpal, K., Henkemeyer, M., Rossant, J. Fgfr-1 is required for embryonic growth and mesodermal patterning during mouse gastrulation. *Genes Dev*. **1994**; 8, 3032-3044.
183. Cobden, S.B., Ozturk, K., Duman, S., Esen, H., Aktan, T.M., Avunduk, M.C., Elsurer, C. Treatment of acute vocal fold injury with platelet-rich plasma. *J Voice*. **2016**; 30, 731-735.
184. Filant, J., DeMayo, F.J., Pru, J.K., Lydon, J.P., Spencer, T.E. Fibroblast growth factor receptor two (FGFR2) regulates uterine epithelial integrity and fertility in mice. *Biol Reprod*. **2014**; 90, 7.
185. Kuroda, K., Yashiro, M., Miki, Y., Sera, T., Yamamoto, Y., Sugimoto, A., Nishimura, S., Kushiyama, S., Togano, S., Okuno, T., Ohira, M. Circulating tumor cells with FGFR2 expression might be useful to identify patients with existing FGFR2-overexpressing tumor. *Cancer Sci*. **2020**; 111, 4500-4509.
186. Chakraborty, D., Zhu, H., Jungel, A., Summa, L., Li, Y.N., Matei, A.E., Zhou, X., Huang, J., Trinh-Minh, T., Chen, C.W., *et al*. Fibroblast growth factor receptor 3 activates a network of profibrotic signaling pathways to promote fibrosis in systemic sclerosis. *Sci Transl Med*. **2020**; 12, eaaz5506.
187. Western, P.S., Ralli, R.A., Wakeling, S.I., Lo, C., van den Bergen, J.A., Miles, D.C., Sinclair, A.H. Mitotic arrest in teratoma susceptible fetal male germ cells. PLoS One. 2011;6(6):e20736.
188. Easter, M., Garth, J., Harris, E.S., Shei, R.J., Helton, E.S., Wei, Y., Denson, R., Zaharias, R., Rowe, S.M., Geraghty, P., *et al*. Fibroblast growth factor receptor 4 deficiency mediates airway inflammation in the adult healthy lung ? *Front Med (Lausanne)*. **2020**; 7, 317.

189. Saito, A., Higuchi, I., Nakagawa, M., Saito, M., Uchida, Y., Inose, M., Kasai, T., Niiyama, T., Fukunaga, H., Arimura, K., Osame, M. An overexpression of fibroblast growth factor (FGF) and FGF receptor 4 in a severe clinical phenotype of facioscapulohumeral muscular dystrophy. *Muscle Nerve*. **2000**; 23, 490-497.
190. Bluteau, G., Zhuang, L., Amann, R., Trueb, B. Targeted disruption of the intracellular domain of receptor FgfrL1 in mice. *PLoS One*. **2014**; 9, e105210.
191. Wang, L., He, J., Hu, H., Tu, L., Sun, Z., Liu, Y., Luo, F. Lung CSC-derived exosomal miR-210-3p contributes to a pro-metastatic phenotype in lung cancer by targeting FGFRL1. *J Cell Mol Med*. **2020**; 24, 6324-6339.
192. Nikuei, P., Rajaei, M., Roozbeh, N., Mohseni, F., Poordarvishi, F., Azad, M., Haidari, S. Diagnostic accuracy of sFlt1/PlGF ratio as a marker for preeclampsia. *BMC Pregnancy Childbirth*. **2020**; 20, 80.
193. Lee, J.Y., Hong, S.H., Shin, M., Heo, H.R., Jang, I.H. Blockade of FLT4 suppresses metastasis of melanoma cells by impaired lymphatic vessels. *Biochem Biophys Res Commun*. **2016**; 478, 733-738.
194. Leedom, A.J., Sullivan, A.B., Dong, B., Lau, D., Gronert, K. Endogenous LXA4 circuits are determinants of pathological angiogenesis in response to chronic injury. *Am J Pathol*. **2010**; 176, 74-84.
195. Zhang, M., Ueki, S., Kimura, S., Yoshida, O., Castellaneta, A., Ozaki, K.S., Demetris, A.J., Ross, M., Vodovotz, Y., Thomson, A.W., *et al*. Roles of dendritic cells in murine hepatic warm and liver transplantation-induced cold ischemia/reperfusion injury. *Hepatology*. **2013**; 57, 1585-1596.
196. Bohannon, J., Cui, W., Sherwood, E., Toliver-Kinsky, T. Dendritic cell modification of neutrophil responses to infection after burn injury. *J Immunol*. **2010**; 185, 2847-2853.
197. Levi, N.J., Wilson, C.W., Redweik, G.A.J., Gray, N.W., Grzybowski, C.W., Lenkey, J.A., Moseman, A.W., Bertsch, A.D., Dao, N., Walsh, H.E. Obesity-related cellular stressors regulate gonadotropin releasing hormone gene expression via c-Fos/AP-1. *Mol Cell Endocrinol*. **2018**; 478, 97-105.
198. Chermula, B., Brazert, M., Jeseta, M., Ozegowska, K., Kocherova, I., Jankowski, M., Celichowski, P., Sujka-Kordowska, P., Konwerska, A., Piotrowska-Kempisty, H., *et al*. Transcriptomic pattern of genes regulating protein response and status of mitochondrial activity are related to oocyte maturational competence - a transcriptomic study. *Int J Mol Sci*. **2019**; 20, 2238.
199. Lin, H.C., Mao, S.C., Gean, P.W. Block of gamma-aminobutyric acid-A receptor insertion in the amygdala impairs extinction of conditioned fear. *Biol Psychiatry*. **2009**; 66, 665-673.
200. Lainez, S., Valente, P., Ontoria-Oviedo, I., Estevez-Herrera, J., Camprubi-Robles, M., Ferrer-Montiel, A., Planells-Cases, R. GABAA receptor associated protein (GABARAP) modulates TRPV1 expression and channel function and desensitization. *FASEB J*. **2010**; 24, 1958-1970.
201. de Brito Alves, J.L., de Oliveira, J.M., Ferreira, D.J., Barros, M.A., Nogueira, V.O., Alves, D.S., Vidal, H., Leandro, C.G., Lagranha, C.J., Pirola, L., da Costa-Silva, J.H. Maternal protein restriction induced-hypertension is associated to oxidative disruption at transcriptional and functional levels in the medulla oblongata. *Clin Exp Pharmacol Physiol*. **2016**; 43, 1177-1184.
202. Jamart, C., Naslain, D., Gilson, H., Francaux, M. Higher activation of autophagy in skeletal muscle of mice during endurance exercise in the fasted state. *Am J Physiol Endocrinol Metab*. **2013**; 305, E964-E974.
203. Brigger, D., Torbett, B.E., Chen, J., Fey, M.F., Tschan, M.P.. Inhibition of GATE-16 attenuates ATRA-induced neutrophil differentiation of APL cells and interferes with autophagosome formation. *Biochem Biophys Res Commun*. **2013**; 438, 283-288.
204. Cho, H.S., Park, S.Y., Kim, S.M., Kim, W.J., Jung, J.Y. Autophagy-related protein MAP1LC3C plays a crucial role in odontogenic differentiation of human dental pulp cells. *Tissue Eng Regen Med*. **2020**; doi: 10.1007/s13770-020-00310-3
205. Fatemi, S.H., Folsom ,T.D., Thuras, P.D. Deficits in GABA(B) receptor system in schizophrenia and mood disorders: a postmortem study. *Schizophr Res*. **2011**; 128, 37-43.
206. Hanack, C., Moroni, M., Lima, W.C., Wende, H., Kirchner, M., Adelfinger, L., Schrenk-Siemens, K., Tappe-Theodor, A., Wetzel, C., Kuich, P.H., *et al*. GABA blocks pathological but not acute TRPV1 pain signals. *Cell*. **2015**; 160, 759-770.
207. Heskin-Sweezie, R., Titley, H.K., Baizer, J.S., Broussard, D.M. Type B GABA receptors contribute to the restoration of balance during vestibular compensation in mice. *Neuroscience*. **2010**; 169, 302-314.
208. Chau, D.K., Choi, A.Y., Yang, W., Leung, W.N., Chan, C.W. Downregulation of glutamatergic and GABAergic proteins in valproric acid associated social impairment during adolescence in mice. *Behav Brain Res*. **2017**; 316, 255-260.
209. Homanics, G.E., Elsen, F.P., Ying, S.W., Jenkins, A., Ferguson, C., Sloat, B., Yuditskaya, S., Goldstein, P.A., Kralic, J.E., Morrow, A.L., Harrison, N.L. A gain-of-function mutation in the GABA receptor produces synaptic and behavioral abnormalities in the mouse. *Genes Brain Behav*. 2005; 4, 10-19.
210. Obradovic, A.L., Scarpa, J., Osuru, H.P., Weaver, J.L., Park, J.Y., Pathirathna, S., Peterkin, A., Lim, Y., Jagodic, M.M., Todorovic, S.M., Jevtovic-Todorovic, V. Silencing the $\alpha$2 subunit of $\gamma$-aminobutyric acid type A receptors in rat dorsal root ganglia reveals its major role in antinociception posttraumatic nerve injury. *Anesthesiology*. 2015; 123, 654-667.

211. Galyamina, A.G., Kovalenko, I.L., Smagin, D.A., Kudryavtseva, N.N. Altered expression of neurotransmitters systems' genes in the ventral tegmental area of depressive male mice: data of RNA-Seq. *Zh Vyssh Nerv Deiat Im I P Pavlova.* **2017**; 67, 113-128.
212. Zheng, X., Liang, L., Hei, C., Yang, W., Zhang, T., Wu, K., Qin, Y., Chang, Q. Bilateral olfactory mucosa damage induces the disappearance of olfactory glomerulus and reduces the expression of extrasynaptic α5GABAARs in the hippocampus in early postnatal sprague dawley rats. *Neurotox Res*. **2018**; 34, 353-362.
213. Liu, M., Fitzgibbon, M., Wang, Y., Reilly, J., Qian, X., O'Brien, T., Clapcote, S., Shen, S., Roche, M. Ulk4 regulates GABAergic signaling and anxiety-related behavior. *Transl Psychiatry*. **2018**; 8, 43.
214. Kim, H., Kim, Y., Bae, S., Lim, S.H., Jang, M., Choi, J., Jeon, J., Hwang, Y.I., Kang, J.S., Lee, W.J. Vitamin C deficiency causes severe defects in the development of the neonatal cerebellum and in the motor behaviors of Gulo(-/-) mice. *Antioxid Redox Signal*. **2015**; 23, 1270-1283.
215. Muinos-Gimeno, M., Espinosa-Parrilla, Y., Guidi, M., Kagerbauer, B., Sipila, T., Maron, E., Pettai, K., Kananen, L., Navines, R., Martin-Santos, R., *et al*. Human microRNAs miR-22, miR-138-2, miR-148a, and miR-488 are associated with panic disorder and regulate several anxiety candidate genes and related pathways. *Biol Psychiatry*. **2011**; 69, 526-533.
216. Li, Z., Cogswell, M., Hixson, K., Brooks-Kayal, A.R., Russek, S.J. Nuclear respiratory factor 1 (NRF-1) controls the activity dependent transcription of the GABA-A receptor beta 1 subunit gene in neurons. *Front Mol Neurosci*. **2018**; 11, 285.
217. Kimura, T., Ishikawa, K., Sakasegawa, Y., Teruya, K., Sata, T., Schatzl, H., Doh-ura, K. GABAA receptor subunit beta1 is involved in the formation of protease-resistant prion protein in prion-infected neuroblastoma cells. *FEBS Lett*. **2010**; 584, 1193-1198.
218. Bo, T., Wang, T.M., Zhu, X.H., Li, J., Li, X.F., Chen, Y., Mao, D.A. Short-term effects of recurrent neonatal seizures on gamma-aminobutyric acid A receptor alpha1 and beta2 subunit expression in the rat brain. *Zhongguo Dang Dai Er Ke Za Zhi (Chinese Journal of Contemporary Pediatrics)*. **2008**; 10, 371-375.
219. Homanics, G.E., DeLorey, T.M., Firestone, L.L., Quinlan, J.J., Handforth, A., Harrison, N.L., Krasowski, M.D., Rick, C.E., Korpi, E.R., Makela, R., *et al*. Mice devoid of gamma-aminobutyrate type A receptor beta3 subunit have epilepsy, cleft palate, and hypersensitive behavior. *Proc Natl Acad Sci USA*. **1997**; 94, 4143-4148.
220. Tunc-Ozcan, E., Ullmann, T.M., Shukla, P.K., Redei, E.E. Low-dose thyroxine attenuates autism-associated adverse effects of fetal alcohol in male offspring's social behavior and hippocampal gene expression. *Alcohol Clin Exp Res*. **2013**; 37, 1986-1995.
221. Xu, W., Wang, K., Chen, Y., Liang, X.T., Yu, M.K., Yue, H., Tierney, M.L. Sperm gamma-aminobutyric acid type A receptor delta subunit (GABRD) and its interaction with purinergic P2X2 receptors in progesterone-induced acrosome reaction and male fertility. *Reprod Fertil Dev*. **2017**; 29, 2060-2072.
222. Aoki, C., Sabaliauskas, N., Chowdhury, T., Min, J.Y., Colacino, A.R., Laurino, K., Barbarich-Marsteller, N.C. Adolescent female rats exhibiting activity-based anorexia express elevated levels of GABA(A) receptor α4 and δ subunits at the plasma membrane of hippocampal CA1 spines. *Synapse*. **2012**; 66, 391-407.
223. Hengen, K.B., Nelson, N.R., Stang, K.M., Johnson, S.M., Crader, S.M., Watters, J.J., Mitchell, G.S., Behan, M. Increased GABA(A) receptor ε-subunit expression on ventral respiratory column neurons protects breathing during pregnancy. *PLoS One*. **2012**; 7, e30608.
224. Irnaten, M., Walwyn, W.M., Wang, J., Venkatesan, P., Evans, C., Chang, K.S., Andresen, M.C., Hales, T.G., Mendelowitz, D. Pentobarbital enhances GABAergic neurotransmission to cardiac parasympathetic neurons, which is prevented by expression of GABA(A) epsilon subunit. *Anesthesiology*. **2002**; 97, 717-724.
225. Kratsman, N., Getselter, D., Elliott, E. Sodium butyrate attenuates social behavior deficits and modifies the transcription of inhibitory/excitatory genes in the frontal cortex of an autism model. *Neuropharmacology*. **2016**; 102, 136-145.
226. Ren, Z., Sahir, N., Murakami, S., Luellen, B.A., Earnheart, J.C., Lal, R., Kim, J.Y., Song, H., Luscher, B. Defects in dendrite and spine maturation and synaptogenesis associated with an anxious-depressive-like phenotype of GABAA receptor-deficient mice. *Neuropharmacology*. **2015**; 88, 171-179.
227. van der Kooij, M.A., Grosse, J., Zanoletti, O., Papilloud, A., Sandi, C. The effects of stress during early postnatal periods on behavior and hippocampal neuroplasticity markers in adult male mice. *Neuroscience*. **2015**; 311, 508-518.
228. Delahanty, R.J., Zhang, Y., Bichell, T.J., Shen, W., Verdier, K., Macdonald, R.L., Xu, L., Boyd, K., Williams, J., Kang, J.Q. Beyond epilepsy and autism: disruption of GABRB3 causes ocular hypopigmentation. *Cell Rep*. **2016**; 17, 3115-3124.
229. Manohar, S., Dahar, K., Adler, H.J., Dalian, D., Salvi, R. Noise-induced hearing loss: neuropathic pain via Ntrk1 signaling. *Mol Cell Neurosci*. **2016**; 75, 101-112.
230. Li, Y., Huang, Y., Zhou, C., Jiang, P.C., Pan, W. MiR-320c prevents the malignant development of cervical cancer by regulating GABRP level. *Eur Rev Med Pharmacol Sci*. **2020**; 24, 8731-8739.
231. Sung, H.Y., Yang, S.D., Ju, W., Ahn, J.H. Aberrant epigenetic regulation of GABRP associates with aggressive phenotype of ovarian cancer. *Exp Mol Med*. **2017**; 49, e335.

232. Zheng, W., Xie, W., Zhang, J., Strong, J.A., Wang, L., Yu, L., Xu, M., Lu, L. Function of gamma-aminobutyric acid receptor/channel rho 1 subunits in spinal cord. *J Biol Chem*. **2003**; 278, 48321-48329.
233. Zhu, F., Feng, M., Sinha, R., Murphy, M.P., Luo, F., Kao, K.S., Szade, K., Seita, J., Weissman, I.L. The GABA receptor GABRR1 is expressed on and functional in hematopoietic stem cells and megakaryocyte progenitors. *Proc Natl Acad Sci USA*. **2019**; 116, 18416-18422.
234. Tadavarty, R., Hwang, J., Rajput, P.S., Soja, P.J., Kumar, U., Sastry, B.R. Are presynaptic GABA-Cϱ2 receptors involved in anti-nociception? *Neurosci Lett*. **2015**; 606, 145-150.
235. Mann, P.E. Gene expression profiling during pregnancy in rat brain tissue. *Brain Sci*. **2014**; 4, 125-135.
236. Pienimaeki-Roemer, A., Konovalova, T., Musri, M.M., Sigruener, A., Boettcher, A., Meister, G., Schmitz, G. Transcriptomic profiling of platelet senescence and platelet extracellular vesicles. *Transfusion*. **2017**; 57, 144-156.
237. Michalovicz, L.T., Konat, G.W. Peripherally restricted acute phase response to a viral mimic alters hippocampal gene expression. *Metab Brain Dis*. **2014**; 29, 75-86.
238. Sugiyama, C., Yamamoto, M., Kotani, T., Kikkawa, F., Murata, Y., Hayashi, Y.. Fertility and pregnancy-associated ß-cell proliferation in mice deficient in proglucagon-derived peptides. *PLoS One.* **2012**; 7, e43745.
239. Sun, S., Liu, S., Luo, J., Chen, Z., Li, C., Loor, J.J., Cao, Y. Repeated pregnant mare serum gonadotropin-mediated oestrous synchronization alters gene expression in the ovaries and reduces reproductive performance in dairy goats. *Reprod Domest Anim*. **2019**; 54, 873-881.
240. Cui, W., He, X., Zhai, X., Zhang, H., Zhang, Y., Jin, F., Song, X., Wu, D., Shi, Q., Li, L. CARF promotes spermatogonial self-renewal and proliferation through Wnt signaling pathway. *Cell Discov*. **2020**; 6, 85.
241. Wang, M., Guo, Y., Wang, M., Zhou, T., Xue, Y., Du, G., Wei, X., Wang, J., Qi, L., Zhang, H., *et al*. The Glial Cell-Derived Neurotrophic Factor (GDNF)-responsive phosphoprotein landscape identifies raptor phosphorylation required for spermatogonial progenitor cell proliferation. *Mol Cell Proteomics*. **2017**; 16, 982-997.
242. Ravula, A.R., Yenugu S. Effect of long-term treatment with a mixture of pyrethroids on the expression of genes that govern male germ cell production in rats. *J Biochem Mol Toxicol*. **2020**; 14, e22654.
243. Kaushik, A., Anand, S., Bhartiya, D. Altered biology of testicular VSELs and SSCs by neonatal endocrine disruption results in defective spermatogenesis, reduced fertility and tumor initiation in adult mice. *Stem Cell Rev Rep*. **2020**; 16, 893-908.
244. McDonagh, S.C., Lee, J., Izzo, A., Brubaker, P.L. Role of glial cell-line derived neurotropic factor family receptor alpha2 in the actions of the glucagon-like peptides on the murine intestine. *Am J Physiol Gastrointest Liver Physiol*. **2007**; 293, G461-G468.
245. Oppenheim, R.W., Houenou, L.J., Parsadanian, A.S., Prevette, D., Snider, W.D., Shen, L. Glial cell line-derived neurotrophic factor and developing mammalian motoneurons: regulation of programmed cell death among motoneuron subtypes. *J Neurosci*. **2000**; 20, 5001-5011.
246. Wang, S., Albers, K.M. Behavioral and cellular level changes in the aging somatosensory system. *Ann N Y Acad Sci*. **2009**; 1170, 745-749.
247. Jankowski, M.P., Rau, K.K., Soneji, D.J., Anderson, C.E., Koerber, H.R. Enhanced artemin/GFRα3 levels regulate mechanically insensitive, heat-sensitive C-fiber recruitment after axotomy and regeneration. *J Neurosci*. **2010**; 30, 16272-16283.
248. Lindfors, P.H., Lindahl, M., Rossi, J., Saarma, M., Airaksinen, M.S. Ablation of persephin receptor glial cell line-derived neurotrophic factor family receptor alpha4 impairs thyroid calcitonin production in young mice. *Endocrinology*. **2006**;147, 2237-2244.
249. Yang, J., Lindahl, M., Lindholm, P., Virtanen, H., Coffey, E., Runeberg-Roos, P., Saarma, M. PSPN/GFRalpha4 has a significantly weaker capacity than GDNF/GFRalpha1 to recruit RET to rafts, but promotes neuronal survival and neurite outgrowth. *FEBS Lett*. **2004**; 569, 267-271.
250. Jorgensen, J.O.L., Juul, A. Therapy of endocrine disease: growth hormone replacement therapy in adults: 30 years of personal clinical experience. *Eur J Endocrinol*. **2018**; 179, R47-R56.
251. Regan, S.L.P., Knight, P.G., Yovich, J.L., Arfuso, F., Dharmarajan, A. Growth hormone during in vitro fertilization in older women modulates the density of receptors in granulosa cells, with improved pregnancy outcomes. *Fertil Steril*. **2018**; 110, 1298-1310.
252. Kirchhoff, S., Kim, J.S., Hagendorff, A., Thonnissen, E., Kruger, O., Lamers, W.H., Willecke, K. Abnormal cardiac conduction and morphogenesis in connexin40 and connexin43 double-deficient mice. *Circ Res*. **2000**; 87, 399-405.
253. Lanner, F., Lee, K.L., Ortega, G.C., Sohl, M., Li, X., Jin, S., Hansson, E.M., Claesson-Welsh, L., Poellinger, L., Lendahl, U., Farnebo, F. Hypoxia-induced arterial differentiation requires adrenomedullin and notch signaling. *Stem Cells Dev*. **2013**; 22, 1360-1369.
254. Khan, M.M., Zaheer, S., Thangavel, R., Patel, M., Kempuraj, D., Zaheer, A. Absence of glia maturation factor protects dopaminergic neurons and improves motor behavior in mouse model of parkinsonism. *Neurochem Res*. **2015**; 40, 980-990.

255. Xu, F.F., Zhang, Z.B., Wang, Y.Y., Wang, T.H. Brain-derived glia maturation factor β participates in lung injury induced by acute cerebral ischemia by increasing ROS in endothelial cells. *Neurosci Bull*. **2018**; 34, 1077-1090.
256. Cheng, Z., Combs, M., Zhu, Q., Xia, P., Opheim, Z., Parker, J., Mack, C.P., Taylor, J.M. Genome-wide RNAi screen identifies regulators of cardiomyocyte necrosis. *ACS Pharmacol Transl Sci*. **2019**; 2, 361-371.
257. Skinner MK, Schmidt M, Savenkova MI, Sadler-Riggleman I, Nilsson EE. Regulation of granulosa and theca cell transcriptomes during ovarian antral follicle development. Mol Reprod Dev. 2008 Sep;75(9):1457-72.
258. Zuo, G., Zhang, D., Mu, R., Shen, H., Li, X., Wang, Z., Li, H., Chen, G. Resolvin D2 protects against cerebral ischemia/reperfusion injury in rats. *Mol Brain*. **2018**; 11, 9.
259. Malek, N., Kostrzewa, M., Makuch, W., Pajak, A., Kucharczyk, M., Piscitelli, F., Przewlocka, B., Di Marzo, V., Starowicz, K. The multiplicity of spinal AA-5-HT anti-nociceptive action in a rat model of neuropathic pain. *Pharmacol Res*. **2016**; 111, 251-263.
260. Cecconi, S., Rossi, G., Oddi, S., Di Nisio, V., Maccarrone, M. Role of major endocannabinoid-binding receptors during mouse oocyte maturation. *Int J Mol Sci*. **2019**; 20, 2866.
261. Naderi, N., Majidi, M., Mousavi, Z., Khoramian Tusi, S., Mansouri, Z., Khodagholi, F. The interaction between intrathecal administration of low doses of palmitoylethanolamide and AM251 in formalin-induced pain related behavior and spinal cord IL1-β expression in rats. *Neurochem Res*. **2012**; 37, 778-785.
262. Higuchi, S., Ahmad, T.R., Argueta, D.A., Perez, P.A., Zhao, C., Schwartz, G.J., DiPatrizio, N.V., Haeusler, R.A. Bile acid composition regulates GPR119-dependent intestinal lipid sensing and food intake regulation in mice. *Gut*. **2020**; 69, 1620-1628.
263. Overton, H.A., Babbs, A.J., Doel, S.M., Fyfe, M.C., Gardner, L.S., Griffin, G., Jackson, H.C., Procter, M.J., Rasamison, C.M., Tang-Christensen, M., *et al*. Deorphanization of a G protein-coupled receptor for oleoylethanolamide and its use in the discovery of small-molecule hypophagic agents. *Cell Metab*. **2006**; 3, 167-175.
264. He, D., Liu, L., Wang, Y., Sheng, M. A novel genes signature associated with the progression of polycystic ovary syndrome. *Pathol Oncol Res*. **2020**; 26, 575-582.
265. Rivera, P., Tovar, R., Ramirez-Lopez, M.T., Navarro, J.A., Vargas, A., Suarez, J., Fonseca, F.R. Sex-specific anxiety and prefrontal cortex glutamatergic dysregulation are long-term consequences of pre-and postnatal exposure to hypercaloric diet in a rat model. *Nutrients*. **2020**; 12, 1829.
266. Mead, A.N., Stephens, D.N. Involvement of AMPA receptor GluR2 subunits in stimulus-reward learning: evidence from glutamate receptor Gria2 knock-out mice. *J Neurosci*. **2003**; 23, 9500-9507.
267. Arslan, A.A., Gold, L.I., Mittal, K., Suen, T.C., Belitskaya-Levy, I., Tang, M.S., Toniolo, P. Gene expression studies provide clues to the pathogenesis of uterine leiomyoma: new evidence and a systematic review. Hum Reprod. 2005 Apr;20(4):852-63.
268. Sagata, N., Iwaki, A., Aramaki, T., Takao, K., Kura, S., Tsuzuki, T., Kawakami, R., Ito, I., Kitamura, T., Sugiyama, H., *et al*. Comprehensive behavioural study of GluR4 knockout mice: implication in cognitive function. *Genes Brain Behav*. **2010**; 9, 899-909.
269. Gray, A.L., Hyde, T.M., Deep-Soboslay, A., Kleinman, J.E., Sodhi, M.S. Sex differences in glutamate receptor gene expression in major depression and suicide. *Mol Psychiatry*. **2015**; 20, 1057-1068.
270. Descalzi, G., Chen, T., Koga, K., Li, X.Y., Yamada, K., Zhuo, M. Cortical GluK1 kainate receptors modulate scratching in adult mice. *J Neurochem*. **2013**; 126, 636-650.
271. Kolber, B.J., Montana, M.C., Carrasquillo, Y., Xu, J., Heinemann, S.F., Muglia, L.J., Gereau, R.W. 4th. Activation of metabotropic glutamate receptor 5 in the amygdala modulates pain-like behavior. *J Neurosci*. **2010**; 30, 8203-8213.
272. Aristidou, C., Theodosiou, A., Ketoni, A., Bak, M., Mehrjouy, M.M., Tommerup, N., Sismani, C. Cryptic breakpoint identified by whole-genome mate-pair sequencing in a rare paternally inherited complex chromosomal rearrangement. *Mol Cytogenet*. **2018**; 11, 34.
273. Zhang, W.G., Zhang, L.C., Peng, Z.D., Zeng, Y.M. Intrathecal injection of GluR6 antisense oligodeoxynucleotides alleviates acute inflammatory pain of rectum in rats. *Neurosci Bull*. **2009**; 25, 319-323.
274. Takenouchi, T., Hashida, N., Torii, C., Kosaki, R., Takahashi, T., Kosaki, K. 1p34.3 deletion involving GRIK3: Further clinical implication of GRIK family glutamate receptors in the pathogenesis of developmental delay. *Am J Med Genet A*. **2014**; 164A, 456-460.
275. Feng, Q., Li, Y., Lu, X., Yu, Y., Yuan, G., Sun, J., Tian, C., Hu, L., Xu, G., An, L., Du, P. Agaricus blazei polypeptide exerts a protective effect on D-galactose-induced aging mice via the Keap1/Nrf2/ARE and P53/Trim32 signaling pathways. *J Food Biochem*. **2021**; 45, e13555.
276. Xu, Y., Wang, J., Rao, S., Ritter, M., Manor, L.C., Backer, R., Cao, H., Cheng, Z., Liu, S., Liu, Y., et al. An integrative computational approach to evaluate genetic markers for bipolar disorder. *Sci Rep*. **2017**; 7, 6745.

277. Wei, B., Li, L., He, A., Zhang, Y., Sun, M., Xu, Z. Hippocampal NMDAR-Wnt-Catenin signaling disrupted with cognitive deficits in adolescent offspring exposed to prenatal hypoxia. *Brain Res*. 2**016**; 1631, 157-164.
278. Hu, L., Han, B., Zhao, X., Mi, L., Song, Q., Wang, J., Song, T., Huang, C. Chronic early postnatal scream sound stress induces learning deficits and NMDA receptor changes in the hippocampus of adult mice. *Neuroreport*. **2016**; 27, 397-403.
279. Holehonnur, R., Phensy, A.J., Kim, L.J., Milivojevic, M., Vuong, D., Daison, D.K., Alex, S., Tiner, M., Jones, L.E., Kroener, S., Ploski, J.E. Increasing the GluN2A/GluN2B ratio in neurons of the mouse basal and lateral amygdala inhibits the modification of an existing fear memory trace. *J Neurosci*. **2016**; 36, 9490-9504.
280. Kleiber, M.L., Wright, E., Singh, S.M. Maternal voluntary drinking in C57BL/6J mice: advancing a model for fetal alcohol spectrum disorders. *Behav Brain Res*. **2011**; 223, 376-387.
281. Liu, J., Shelkar, G.P., Sarode, L.P., Gawande, D.Y., Zhao, F., Clausen, R.P., Ugale, R.R., Dravid, S.M. Facilitation of GluN2C-containing NMDA receptors in the external globus pallidus increases firing of fast spiking neurons and improves motor function in a hemiparkinsonian mouse model. *Neurobiol Dis*. **2021**; 150, 105254.
282. Salimando, G.J., Hyun, M., Boyt, K.M., Winder, D.G. BNST GluN2D-Containing NMDA receptors influence anxiety- and depressive-like behaviors and modulatecell-specific excitatory/inhibitory synaptic balance. *J Neurosci*. **2020**; 40, 3949-3968.
283. Kaneko, T., Kaneko, M., Chokechanachaisakul, U., Kawamura, J., Kaneko, R., Sunakawa, M., Okiji, T., Suda, H. Artificial dental pulp exposure injury up-regulates antigen-presenting cell-related molecules in rat central nervous system. *J Endod*. **2010**; 36, 459-464.
284. Creed, M., Kaufling, J., Fois, G.R., Jalabert, M., Yuan, T., Luscher, C., Georges, F., Bellone, C. Cocaine exposure enhances the activity of ventral tegmental area dopamine neurons via calcium-impermeable NMDARs. *J Neurosci*. **2016**; 36, 10759-10768.
285. Dela Pena, I., Bang, M., Lee, J., de la Pena, J.B., Kim, B.N., Han, D.H., Noh, M., Shin, C.Y., Cheong, J.H. Common prefrontal cortical gene expression profiles between adolescent SHR/NCrl and WKY/NCrl rats which showed inattention behavior. *Behav Brain Res*. **2015**; 291, 268-276.
286. Niemann, S., Kanki, H., Fukui, Y., Takao, K., Fukaya, M., Hynynen, M.N., Churchill, M.J., Shefner, J.M., Bronson, R.T., Brown, R.H. Jr, *et al*. Genetic ablation of NMDA receptor subunit NR3B in mouse reveals motoneuronal and nonmotoneuronal phenotypes. *Eur J Neurosci*. **2007**; 26, 1407-1420.
287. Li, M.D., Wang, J., Niu, T., Ma, J.Z., Seneviratne, C., Ait-Daoud, N., Saadvandi, J., Morris, R., Weiss, D., Campbell, J., *et al*. Transcriptome profiling and pathway analysis of genes expressed differentially in participants with or without a positive response to topiramate treatment for methamphetamine addiction. *BMC Med Genomics*. **2014**; 7, 65.
288. Goswami, D.B., Jernigan, C.S., Chandran, A., Iyo, A.H., May, W.L., Austin, M.C., Stockmeier, C.A., Karolewicz, B. Gene expression analysis of novel genes in the prefrontal cortex of major depressive disorder subjects. *Prog Neuropsychopharmacol Biol Psychiatry*. **2013**; 43, 126-133.
289. Huang, H., Qing, X., Li, H. Isoflurane preconditioning protects the myocardium against ischemia and reperfusion injury by upregulating GRM1 expression. Curr Neurovasc Res. 2020;17(2):171-176.
290. Richard, J.M., Berridge, K.C. Metabotropic glutamate receptor blockade in nucleus accumbens shell shifts affective valence towards fear and disgust. *Eur J Neurosci*. **2011**; 33, 736-747.
291. Davidson, S., Golden, J.P., Copits, B.A., Ray, P.R., Vogt, S.K., Valtcheva, M.V., Schmidt, R.E., Ghetti, A., Price, T.J., Gereau, R.W. 4th. Group II mGluRs suppress hyperexcitability in mouse and human nociceptors. *Pain*. **2016**; 157, 2081-2088.
292. Fujioka, R., Nii, T., Iwaki, A., Shibata, A., Ito, I., Kitaichi, K., Nomura, M., Hattori, S., Takao, K., Miyakawa, T., Fukumaki, Y. Comprehensive behavioral study of mGluR3 knockout mice: implication in schizophrenia related endophenotypes. *Mol Brain*. **2014**; 7, 31.
293. Walker, A.G., Wenthur, C.J., Xiang, Z., Rook, J.M., Emmitte, K.A., Niswender, C.M., Lindsley, C.W., Conn, P.J. Metabotropic glutamate receptor 3 activation is required for long-term depression in medial prefrontal cortex and fear extinction. *Proc Natl Acad Sci USA*. **2015**; 112, 1196-1201.
294. Ponnazhagan, R., Harms, A.S., Thome, A.D., Jurkuvenaite, A., Gogliotti, R., Niswender, C.M., Conn, P.J., Standaert, D.G. The Metabotropic glutamate receptor 4 positive allosteric modulator ADX88178 inhibits inflammatory responses in primary microglia. *J Neuroimmune Pharmacol*. **2016**; 11, 231-237.
295. Chandley, M.J., Szebeni, A., Szebeni, K., Crawford, J.D., Stockmeier, C.A., Turecki, G., Kostrzewa, R.M., Ordway, G.A. Elevated gene expression of glutamate receptors in noradrenergic neurons from the locus coeruleus in major depression. *Int J Neuropsychopharmacol*. **2014**; 17, 1569-1578.
296. Lax, N.C., George, D.C., Ignatz, C., Kolber, B.J. The mGluR5 antagonist fenobam induces analgesic conditioned place preference in mice with spared nerve injury. *PLoS One*. **2014**; 9, e103524.
297. Buonaguro, E.F., Morley-Fletcher, S., Avagliano, C., Vellucci, L., Iasevoli, F., Bouwalerh, H., Van Camp, G., Nicoletti, F., Maccari, S., de Bartolomeis, A. Glutamatergic postsynaptic density in early life stress programming: Topographic gene expression of mGlu5 receptors and Homer proteins. *Prog Neuropsychopharmacol Biol Psychiatry*. **2020**; 96, 109725.

298. Marciniak, M., Chruscicka, B., Lech, T., Burnat, G., Pilc, A. Expression of group III metabotropic glutamate receptors in the reproductive system of male mice. *Reprod Fertil Dev*. **2016**; 28, 369-374.
299. Xia, W., Liu, Y., Jiao, J. GRM7 regulates embryonic neurogenesis via CREB and YAP. *Stem Cell Reports*. **2015**; 4, 795-810.
300. Fendt, M., Imobersteg, S., Peterlik, D., Chaperon, F., Mattes, C., Wittmann, C., Olpe, H.R., Mosbacher, J., Vranesic, I., van der Putten, H., *et al*. Differential roles of mGlu(7) and mGlu(8) in amygdala-dependent behavior and physiology. *Neuropharmacology*. **2013**; 72, 215-223.
301. Rossi, F., Marabese, I., De Chiaro, M., Boccella, S., Luongo, L., Guida, F., De Gregorio, D., Giordano, C., de Novellis, V, Palazzo, E., Maione, S. Dorsal striatum metabotropic glutamate receptor 8 affects nocifensive responses and rostral ventromedial medulla cell activity in neuropathic pain conditions. *J Neurophysiol*. **2014**; 111, 2196-2209.
302. Cui, Z., Agarwal, A., da Silva, B.F., Sharma, R., Sabanegh, E. Evaluation of seminal plasma proteomics and relevance of FSH in identification of nonobstructive azoospermia: a preliminary study. *Andrologia*. **2018**; 50, e12999.
303. Dickinson, S.E., Griffin, B.A., Elmore, M.F., Kriese-Anderson, L., Elmore, J.B., Dyce, P.W., Rodning, S.P., Biase, F.H. Transcriptome profiles in peripheral white blood cells at the time of artificial insemination discriminate beef heifers with different fertility potential. *BMC Genomics*. **2018**; 19, 129.
304. Vawter, M.P., Harvey, P.D., DeLisi, L.E. Dysregulation of X-linked gene expression in Klinefelter's syndrome and association with verbal cognition. *Am J Med Genet B Neuropsychiatr Genet*. **2007**; 144B, 728-734.
305. Lynn, R. New evidence for dysgenic fertility for intelligence in the United States. *Soc Biol*. **1999**; 46, 146-153.
306. Takhviji, V., Zibara, K., Azarkeivan, A., Mehrvar, N., Mehrvar, N., Mezginejad, F., Khosravi, A. Fertility and pregnancy in Iranian thalassemia patients: An update on transfusion complications. *Transfus Med*. **2020**; 30, 352-360.
307. Wang F., Yu H., Huang S., Zheng L., Zheng P., Zhang S., Li S., Chen J. Jian-Pi-Yi-Shen regulates EPO and iron recycling protein expressions in anemic rats with chronic kidney disease: accumulation of hypoxia inducible factor-2α via ERK signaling. Evid Based Complement Alternat Med. 2020; 2020: 8894257.
308. Peltoketo, H., Piao, Y., Mannermaa, A., Ponder, B.A., Isomaa, V., Poutanen, M., Winqvist, R., Vihko, R. A point mutation in the putative TATA box, detected in nondiseased individuals and patients with hereditary breast cancer, decreases promoter activity of the 17 beta-hydroxysteroid dehydrogenase type 1 gene 2 (EDH17B2) in vitro. *Genomics*. **1994**; 23, 250-252.
309. He, W., Gauri, M., Li, T., Wang, R., Lin, S.X. Current knowledge of the multifunctional 17β-hydroxysteroid dehydrogenase type 1 (HSD17B1). *Gene*. **2016**; 588, 54-61.
310. Meunier, C.N.J., Cancela, J.M., Fossier, P. Lack of GSK3β activation and modulation of synaptic plasticity by dopamine in 5-HT1A-receptor KO mice. *Neuropharmacology*. **2017**; 113, 124-136.
311. Ahmed, A.A., Ma, W., Ni, Y., Zhou, Q., Zhao, R. Embryonic exposure to corticosterone modifies aggressive behavior through alterations of the hypothalamic pituitary adrenal axis and the serotonergic system in the chicken. *Horm Behav*. **2014**; 65, 97-105.
312. Saudou, F., Amara, D.A., Dierich, A., LeMeur, M., Ramboz, S., Segu, L., Buhot, M.C., Hen, R. Enhanced aggressive behavior in mice lacking 5-HT1B receptor. *Science*. **1994**; 265, 1875-1878.
313. Field, S.L., Marrero, M.G., Dado-Senn, B., Skibiel, A.L., Ramos, P.M., Scheffler, T.L., Laporta, J. Peripheral serotonin regulates glucose and insulin metabolism in Holstein dairy calves. *Domest Anim Endocrinol*. **2021**; 74, 106519.
314. Granados-Soto, V., Arguelles, C.F., Rocha-Gonzalez, H.I., Godinez-Chaparro, B., Flores-Murrieta, F.J., Villalon, C.M. The role of peripheral 5-HT1A, 5-HT1B, 5-HT1D, 5-HT1E and 5-HT1F serotonergic receptors in the reduction of nociception in rats. *Neuroscience*. **2010**; 165, 561-568.
315. Shong, K.E., Oh, C.M., Namkung, J., Park, S., Kim, H. Serotonin regulates de novo lipogenesis in adipose tissues through serotonin receptor 2A. *Endocrinol Metab (Seoul)*. **2020**; 35, 470-479.
316. Gao, W., Guo, N., Zhao, S., Chen, Z., Zhang, W., Yan, F., Liao, H., Chi, K. HTR2A promotes the development of cardiac hypertrophy by activating PI3K-PDK1-AKT-mTOR signaling. *Cell Stress Chaperones*. **2020**; 25, 899-908.
317. Kaushik, G., Xia, Y., Pfau, J.C., Thomas, M.A. Dysregulation of autism-associated synaptic proteins by psychoactive pharmaceuticals at environmental concentrations. *Neurosci Lett*. **2017**; 661, 143-148.
318. Popova, N.K., Naumenko, V.S., Kozhemyakina, R.V., Plyusnina, I.Z. Functional characteristics of serotonin 5-HT2A and 5-HT2C receptors in the brain and the expression of the 5-HT2A and 5-HT2C receptor genes in aggressive and non-aggressive rats. *Neurosci Behav Physiol*. **2010**; 40, 357-361.
319. Park, H., Oh, C.M., Park, J., Park, H., Cui, S., Kim, H.S., Namkung, J., Park, S.K., Pak, H.N., Lee, M.H., et al. Deletion of the serotonin receptor type 3a in mice leads to sudden cardiac death during pregnancy. *Circ J*. **2015**; 79, 1807-1815.

320. Kondo, M., Nakamura, Y., Ishida, Y., Shimada, S. The 5-HT3 receptor is essential for exercise-induced hippocampal neurogenesis and antidepressant effects. *Mol Psychiatry*. **2015**; 20, 1428-1437.
321. Guo, Y., Zhang, H., Gao, J., Wei, S., Song, C., Sun, P., Qiao, M. Study of genes associated with the 'anger-in' and 'anger-out' emotions of humans using a rat model. *Exp Ther Med*. **2015**; 9, 1448-1454.
322. Jin, Y., Wang, L., Duan, Q., Gong, Z., Yang, F., Song, Y. Differential expression of 5-HT-related genes in symptomatic pulmonary embolism patients. *Int J Clin Exp Med*. 2015; 8, 512-518.
323. Niesler, B., Walstab, J., Combrink, S., Moller, D., Kapeller, J., Rietdorf, J., Bonisch, H., Gothert, M., Rappold, G., Bruss, M. Characterization of the novel human serotonin receptor subunits 5-HT3C,5-HT3D, and 5-HT3E. *Mol Pharmacol*. **2007**; 72, 8-17.
324. Bi, W.K., Luan, S.S., Wang, J., Wu, S.S., Jin, X.C., Fu, Y.L., Gao, L., Zhao, J.J., He, Z. FSH signaling is involved in affective disorders. *Biochem Biophys Res Commun*. **2020**; 525, 915-920.
325. Hromadkova, J., Suzuki, Y., Pletts, S., Pyo, J., Ma, T., Chen, Y., Steele, M.A., Guan, L.L. Effect of colostrum feeding strategies on the expression of neuroendocrine genes and active gut mucosa-attached bacterial populations in neonatal calves. *J Dairy Sci*. **2020**; 103, 8629-8642.
326. Littlejohn, B.P., Price, D.M., Neuendorff, D.A., Carroll, J.A., Vann, R.C., Riggs, P.K., Riley, D.G., Long, C.R., Randel, R.D., Welsh, T.H. Influence of prenatal transportation stress-induced differential DNA methylation on the physiological control of behavior and stress response in suckling Brahman bull calves. *J Anim Sci*. **2020**; 98, skz368.
327. Ranzil, S., Ellery, S., Walker, D.W., Vaillancourt, C., Alfaidy, N., Bonnin, A., Borg, A., Wallace, E.M., Ebeling, P.R., Erwich, J.J., Murthi, P. Disrupted placental serotonin synthetic pathway and increased placental serotonin: Potential implications in the pathogenesis of human fetal growth restriction. *Placenta*. **2019**; 84, 74-83.
328. Zhu, H., Xiao, X., Chai, Y., Li, D., Yan, X., Tang, H. MiRNA-29a modulates visceral hyperalgesia in irritable bowel syndrome by targeting HTR7. *Biochem Biophys Res Commun*. **2019**; 511, 671-678.
329. Hernandez, L.L., Grayson, B.E., Yadav, E., Seeley, R.J., Horseman, N.D. High fat diet alters lactation outcomes: possible involvement of inflammatory and serotonergic pathways. *PLoS One*. **2012**; 7, e32598.
330. Wathes, D.C., Cheng, Z., Salavati, M., Buggiotti, L., Takeda, H., Tang, L., Becker, F., Ingvartsen, K.I., Ferris, C., Hostens, M., *et al*. Relationships between metabolic profiles and gene expression in liver and leukocytes of dairy cows in early lactation. *J Dairy Sci*. **2021;** S0022-0302(21)00032-1.
331. Ouyang, X., You, S., Zhang, Y., Zhang, C., Zhang, G., Shao, X., He, F., Hu, L. Transplantation of human amnion epithelial cells improves endometrial regeneration in rat model of intrauterine adhesions. *Stem Cells Dev*. **2020**; 29, 1346-1362.
332. Pashaiasl, M., Ebrahimi, M., Ebrahimie, E. Identification of the key regulating genes of diminished ovarian reserve (DOR) by network and gene ontology analysis. *Mol Biol Rep*. **2016**; 43, 923-937.
333. Muhammad, T., Wan, Y., Sha, Q., Wang, J., Huang, T., Cao, Y., Li, M., Yu, X., Yin, Y., Chan, W.Y., *et al*. IGF2 improves the developmental competency and meiotic structure of oocytes from aged mice. *Aging (Albany NY)*. **2020**; 12, doi: 10.18632/aging.202214.
334. Ramin, N., Thieme, R., Fischer, S., Schindler, M., Schmidt, T., Fischer, B., Navarrete Santos, A. Maternal diabetes impairs gastrulation and insulin and IGF-I receptor expression in rabbit blastocysts. *Endocrinology*. **2010**; 151, 4158-4167.
335. Kuo, Y.C., Au, H.K., Hsu, J.L., Wang, H.F., Lee, C.J., Peng, S.W., Lai, S.C., Wu, Y.C., Ho, H.N., Huang, Y.H. IGF-1R promotes symmetric self-renewal and migration of alkaline phosphatase+ germ stem cells through HIF-2α-OCT4/CXCR4 loop under hypoxia. *Stem Cell Reports*. **2018**; 10, 524-537.
336. Sasaki, Y., Otsuka, K., Arimochi, H., Tsukumo, S.I., Yasutomo, K. Distinct roles of IL-1β and IL-18 in NLRC4-induced autoinflammation. *Front Immunol*. **2020**; 11, 591713.
337. Olkkonen, J., Kouri, V.P., Hynninen, J., Konttinen, Y.T., Mandelin, J. Differentially expressed in chondrocytes 2 (DEC2) increases the expression of IL-1β and is abundantly present in synovial membrane in rheumatoid arthritis. *PLoS One*. **2015**; 10, e0145279.
338. Wang, M., Wu, H., Duan, M., Yang, Y., Wang, G., Che, F., Liu, B., He, W., Li, Q., Zhang, L. SS30, a novel thioaptamer targeting CD123, inhibits the growth of acute myeloid leukemia cells. *Life Sci*. **2019**; 232, 116663.
339. Stirewalt, D.L., Meshinchi, S., Kopecky, K.J., Fan, W., Pogosova-Agadjanyan, E.L., Engel, J.H., Cronk, M.R., Dorcy, K.S., McQuary, A.R., Hockenbery, D., *et al*. Identification of genes with abnormal expression changes in acute myeloid leukemia. *Genes Chromosomes Cancer*. **2008**; 47, 8-20.
340. Liu, B., Zhu, X., Cui, Y., Wang, W., Liu, H., Li, Z., Guo, Z., Ma, S., Li, D., Wang, C., Shi, Y. Consumption of dietary fiber from different sources during pregnancy alters sow gut microbiota and improves performance and reduces inflammation in sows and piglets. *mSystems*. **2021**; 6, e00591-20.
341. Lliberos, C., Liew, S.H., Zareie, P., La Gruta, N.L., Mansell, A., Hutt, K. Evaluation of inflammation and follicle depletion during ovarian ageing in mice. *Sci Rep*. **2021**; 11, 278.

342. Wang, L., Wang, E., Wang, Y., Mines, R., Xiang, K., Sun, Z., Zhou, G., Chen, K.Y., Rakhilin, N., Chao, S., *et al*. miR-34a is a microRNA safeguard for Citrobacter-induced inflammatory colon oncogenesis. *Elife*. **2018**; 7, e39479.
343. Li, S., Fu, X., Wu, T., Yang, L., Hu, C., Wu, R. Role of interleukin-6 and its receptor in endometriosis. *Med Sci Monit*. **2017**; 23, 3801-3807.
344. Sackett, S.D., Otto, T., Mohs, A., Sander, L.E., Strauch, S., Streetz, K.L., Kroy, D.C., Trautwein, C. Myeloid cells require gp130 signaling for protective anti-inflammatory functions during sepsis. *FASEB J*. **2019**; 33, 6035-6044.
345. Gray, S.R., Robinson, M., Nimmo, M.A. Response of plasma IL-6 and its soluble receptors during submaximal exercise to fatigue in sedentary middle-aged men. *Cell Stress Chaperones*. **2008**; 13, 247-51.
346. Sun, Y., Liu, S., Hu, R., Zhou, Q., Li, X. Decreased placental IL9 and IL9R in preeclampsia impair trophoblast cell proliferation, invasion, and angiogenesis. *Hypertens Pregnancy*. **2020**; 39, 228-235.
347. Osterfeld, H., Ahrens, R., Strait, R., Finkelman, F.D., Renauld, J.C., Hogan, S.P. Differential roles for the IL-9/IL-9 receptor alpha-chain pathway in systemic and oral antigen-induced anaphylaxis. *J Allergy Clin Immunol*. **2010**; 125, 469-476.e2.
348. Winship, A., Menkhorst, E., Van Sinderen, M., Dimitriadis, E. Interleukin 11 blockade during mid to late gestation does not affect maternal blood pressure, pregnancy viability or subsequent fertility in mice. *Reprod Biomed Online*. **2018**; 36, 250-258.
349. Zhao, Y., Park, S., Bagchi, M.K., Taylor, R.N., Katzenellenbogen, B.S. The coregulator, repressor of estrogen receptor activity (REA), is a crucial regulator of the timing and magnitude of uterine decidualization. *Endocrinology*. **2013**; 154, 1349-1360.
350. Agthe, M., Garbers, Y., Putoczki, T., Garbers, C. Interleukin-11 classic but not trans-signaling is essential for fertility in mice. *Placenta*. **2017**; 57, 13-16.
351. Liu, T., Ma, Q., Zhang, Y., Ke, S., Yan, K., Chen, X., Wen, Y., Fan, Q., Qiu, X. Interleukin-11 receptor $\alpha$ is overexpressed in human osteosarcoma, and near-infrared-labeled IL-11R$\alpha$ imaging agent could detect osteosarcoma in mouse tumor xenografts. *Tumour Biol*. **2015**; 36, 2369-2375.
352. Hu, X., Zhu, Q., Wang, Y., Wang, L., Li, Z., Mor, G., Liao, A. Newly characterized decidual Tim-3+ Treg cells are abundant during early pregnancy and driven by IL-27 coordinately with Gal-9 from trophoblasts. *Hum Reprod*. **2020**; 35, 2454-2466.
353. Yin, N., Wang, H., Zhang, H., Ge, H., Tan, B., Yuan, Y., Luo, X., Olson, D.M., Baker, P.N., Qi, H. IL-27 induces a pro-inflammatory response in human fetal membranes mediating preterm birth. *Int Immunopharmacol*. **2017**; 50, 361-369.
354. Bassett, J.M., Hanson, C. Prevention of hypoinsulinemia modifies catecholamine effects in fetal sheep. *Am J Physiol Regul Integr Comp Physiol*. **2000**; 278, R1171-R1181.
355. Landrum, M.J., Lee, J.M., Riley, G.R., Jang, W., Rubinstein, W.S., Church, D.M., Maglott, D.R. ClinVar: public archive of relationships among sequence variation and human phenotype. *Nucleic Acids Res*. **2014**; 42, D980-D985.
356. Komura, K., Yoshikawa, Y., Shimamura, T., Chakraborty, G., Gerke, T.A., Hinohara, K., Chadalavada, K., Jeong, S.H., Armenia, J., Du, S.Y., *et al*. ATR inhibition controls aggressive prostate tumors deficient in Y-linked histone demethylase KDM5D. *J Clin Invest*. **2018**; 128, 2979-2995.
357. Mokou, M., Klein, J., Makridakis, M., Bitsika, V., Bascands, J.L., Saulnier-Blache, J.S., Mullen, W., Sacherer, M., Zoidakis, J., Pieske, B., *et al*. Proteomics based identification of KDM5 histone demethylases associated with cardiovascular disease. *EBioMedicine*. **2019**; 41, 91-104.
358. Chou, S.H., Chamberland, J.P., Liu, X., Matarese, G., Gao, C., Stefanakis, R., Brinkoetter, M.T., Gong, H., Arampatzi, K., Mantzoros, C.S. Leptin is an effective treatment for hypothalamic amenorrhea. *Proc Natl Acad Sci USA*. **2011**;108 (16): 6585-6590.
359. Montserrat-de la Paz, S., Perez-Perez, A., Vilarino-Garcia, T., Jimenez-Cortegana, C., Muriana, F.J.G., Millan-Linares, M.C., Sanchez-Margalet, V. Nutritional modulation of leptin expression and leptin action in obesity and obesity-asociated complications. *J Nutr Biochem*. **2020**; 26, 108561.
360. Xue, S., Maluenda, J., Marguet, F., Shboul, M., Quevarec, L., Bonnard, C., Ng, A.Y., Tohari, S., Tan, T.T., Kong, M.K., *et al*. Loss-of-function mutations in LGI4, a secreted ligand involved in schwann cell myelination, are responsible for arthrogryposis multiplex congenita. *Am J Hum Genet*. **2017**; 100, 659-665.
361. Margioula-Siarkou, C., Prapas, Y., Petousis, S., Milias, S., Ravanos, K., Dagklis, T., Kalogiannidis, I., Mavromatidis, G., Haitoglou, C., Prapas, N., Rousso, D. LIF endometrial expression is impaired in women with unexplained infertility while LIF-R expression in all infertility sub-groups. *Cytokine*. **2017**; 96, 166-172.
362. Christiansen, O.B., Nielsen, H.S., Lund, M., Steffensen, R., Varming, K. Mannose-binding lectin-2 genotypes and recurrent late pregnancy losses. *Hum Reprod*. **2009**; 24, 291-299.
363. Chatterjee, S.K., Saha, S., Munoz, M.N.M. Molecular pathogenesis, immunopathogenesis and novel therapeutic strategy against COVID-19. *Front Mol Biosci*. **2020**; 7, 196.
364. Larsen, P., Yong, V.W. The expression of matrix metalloproteinase-12 by oligodendrocytes regulates their maturation and morphological differentiation. *J Neurosci*. **2004**; 24, 7597-7603.

365. Hiden, U., Eyth, C.P., Majali-Martinez, A., Desoye, G., Tam-Amersdorfer, C., Huppertz, B., Ghaffari Tabrizi-Wizsy, N. Expression of matrix metalloproteinase 12 is highly specific for non-proliferating invasive trophoblasts in the first trimester and temporally regulated by oxygen-dependent mechanisms including HIF-1A. *Histochem Cell Biol*. **2018**; 149, 31-42.
366. Mikael, L.G., Pancer, J., Jiang, X., Wu, Q., Caudill, M., Rozen, R. Low dietary folate and methylenetetrahydrofolate reductase deficiency may lead to pregnancy complications through modulation of ApoAI and IFN-$\gamma$ in spleen and placenta, and through reduction of methylation potential. *Mol Nutr Food Res*. **2013**; 57, 661-670.
367. Perez-Sepulveda, A., Espana-Perrot, P.P., Fernandez, X.B., Ahumada, V., Bustos, V., Arraztoa, J.A., Dobierzewska, A., Figueroa-Diesel, H., Rice, G.E., Illanes, S.E. Levels of key enzymes of methionine-homocysteine metabolism in preeclampsia. *Biomed Res Int*. **2013**; 2013, 731962.
368. Mandel, S., Rechavi, G., Gozes, I. Activity-dependent neuroprotective protein (ADNP) differentially interacts with chromatin to regulate genes essential for embryogenesis. *Dev Biol*. **2007**; 303, 814-824.
369. Sotomayor-Zarate, R., Dorfman, M., Paredes, A., Lara, H.E. Neonatal exposure to estradiol valerate programs ovarian sympathetic innervation and follicular development in the adult rat. *Biol Reprod*. **2008**; 78, 673-680.
370. Gong, Y., Wang, L., Chippada-Venkata, U., Dai, X., Oh, W.K., Zhu, J. Constructing Bayesian networks by integrating gene expression and copy number data identifies NLGN4Y as a novel regulator of prostate cancer progression. *Oncotarget*. **2016**; 7, 68688-68707.
371. Bhat, M.A., Sharma, J.B., Roy, K.K., Sengupta, J., Ghosh, D. Genomic evidence of Y chromosome microchimerism in the endometrium during endometriosis and in cases of infertility. *Reprod Biol Endocrinol*. **2019**; 17, 22.
372. Tsutsui, M., Shimokawa, H., Morishita, T., Nakashima, Y., Yanagihara, N. Development of genetically engineered mice lacking all three nitric oxide synthases. *J Pharmacol Sci*. **2006**; 102, 147-154.
374. Wojcik, M., Zieleniak, A., Zurawska-Klis, M., Cypryk, K., Wozniak, L.A. Increased expression of immune-related genes in leukocytes of patients with diagnosed gestational diabetes mellitus (GDM). *Exp Biol Med (Maywood)*. **2016**; 241, 457-465.
375. Hahm, S., Mizuno, T.M., Wu, T.J., Wisor, J.P., Priest, C.A., Kozak, C.A., Boozer, C.N., Peng, B., McEvoy, R.C., Good, P., *et al*. Targeted deletion of the Vgf gene indicates that the encoded secretory peptide precursor plays a novel role in the regulation of energy balance. *Neuron*. **1999**; 23, 537-548.
376. Sainsbury, A., Schwarzer, C., Couzens, M., Herzog, H. Y2 receptor deletion attenuates the type 2 diabetic syndrome of ob/ob mice. *Diabetes*. **2002**; 51, 3420-3427.
377. Nguyen, L.T., Saad, S., Tan, Y., Pollock, C., Chen, H. Maternal high-fat diet induces metabolic stress response disorders in offspring hypothalamus. *J Mol Endocrinol*. **2017**; 59, 81-92.
378. Smiley, R., McCallum, R,. Showkat Ali, M. Decreased level of neuropeptide Y is associated with gastroparesis in male diabetic rats. *Gastroenterology Res*. **2020**; 13, 246-252.
379. Lay, A.C., Barrington, A.F., Hurcombe, J.A., Ramnath, R.D., Graham, M., Lewis, P.A., Wilson, M.C., Heesom, K.J., Butler, M.J., Perrett, R.M., *et al*. A role for NPY-NPY2R signaling in albuminuric kidney disease. *Proc Natl Acad Sci USA*. **2020**; 117, 15862-15873.
380. Edvinsson, A., Hellgren, C., Kunovac Kallak, T., Akerud, H., Skalkidou, A., Stener-Victorin, E., Fornes, R., Spigset, O., Lager, S., Olivier, J., Sundstrom-Poromaa, I. The effect of antenatal depression and antidepressant treatment on placental tissue: a protein-validated gene expression study. *BMC Pregnancy Childbirth*. **2019**; 19, 479.
381. Elnar, A.A., Desor, F., Marin, F., Soulimani, R., Nemos, C. Lactational exposure to low levels of the six indicator non-dioxin-like polychlorinated biphenyls induces DNA damage and repression of neuronal activity, in juvenile male mice. *Toxicology*. **2015**; 328, 57-65.
382. Shebanits, K., Andersson-Assarsson, J.C., Larsson, I., Carlsson, L.M.S., Feuk, L., Larhammar, D. Copy number of pancreatic polypeptide receptor gene NPY4R correlates with body mass index and waist circumference. *PLoS One*. **2018**; 13, e0194668.
383. Mashiko, S., Ishihara, A., Iwaasa, H., Moriya, R., Kitazawa, H., Mitobe, Y., Ito, J., Gomori, A., Matsushita, H., Takahashi, T., *et al*. Effects of a novel Y5 antagonist in obese mice: combination with food restriction or sibutramine. *Obesity (Silver Spring)*. **2008**; 16, 1510-1515.
384. Nagaraja, M.R., Gubbala, S.P., Delphine Silvia, C.R.W., Amanchy, R. Molecular diagnostics of disorders of sexual development: an Indian survey and systems biology perspective. *Syst Biol Reprod Med*. **2019**; 65, 105-120.
385. Jameson, J.L. Of mice and men: The tale of steroidogenic factor-1. *J Clin Endocrinol Metab*. **2004**; 89, 5927-5929.
386. Gregoire, E.P., Stevant, I., Chassot, A.A., Martin, L., Lachambre, S., Mondin, M., de Rooij, D.G., Nef, S., Chaboissier, M.C. NRG1 signalling regulates the establishment of Sertoli cell stock in the mouse testis. *Mol Cell Endocrinol*. **2018**; 478, 17-31.
387. Abe, Y., Namba, H., Kato, T., Iwakura, Y., Nawa, H. Neuregulin-1 signals from the periphery regulate AMPA receptor sensitivity and expression in GABAergic interneurons in developing neocortex. *J Neurosci*. **2011**; 31, 5699-5709.

388. Yan, L., Shamir, A., Skirzewski, M., Leiva-Salcedo, E., Kwon, O.B., Karavanova, I., Paredes, D., Malkesman, O., Bailey, K.R., et al. Neuregulin-2 ablation results in dopamine dysregulation and severe behavioral phenotypes relevant to psychiatric disorders. *Mol Psychiatry*. **2018**; 23, 1233-1243.
389. Diaz-Moran, S., Palencia, M., Mont-Cardona, C., Canete, T., Blazquez, G., Martinez-Membrivesm E., Lopez-Aumatell, R., Sabariego, M., Donaire, R., Moron, I., *et al*. Gene expression in hippocampus as a function of differential trait anxiety levels in genetically heterogeneous NIH-HS rats. *Behav Brain Res*. **2013**; 257, 129-139.
390. Hayes, L.N., Shevelkin, A., Zeledon, M., Steel, G., Chen, P.L., Obie, C., Pulver, A., Avramopoulos, D., Valle, D., Sawa, A., Pletnikov, M.V. Neuregulin 3 knockout mice exhibit behaviors consistent with psychotic disorders. *Mol Neuropsychiatry*. **2016**; 2, 79-87.
391. Jin, Y.J., Pan, X.Y., Jin, Y.J. Neuregulin promotes spermatogonia proliferation in mice. *Zhonghua Nan Ke Xue (National Journal of Andrology)*. **2011**; 17, 406-409.
392. Nugroho, D.B., Ikeda, K., Barinda, A.J., Wardhana, D.A., Yagi, K., Miyata, K., Oike, Y., Hirata, K.I., Emoto, N. Neuregulin-4 is an angiogenic factor that is critically involved in the maintenance of adipose tissue vasculature. *Biochem Biophys Res Commun*. **2018**; 503, 378-384.
393. Kurek Eken, M., Sahin Ersoy, G., Yayla Abide, C., Sanverdi, I., Devranoglu, B., Kutlu, T., Cevik, O. Association between circulating neuregulin 4 levels and metabolic, aterogenic, and AMH profile of polycystic ovary syndrome. *J Obstet Gynaecol*. **2019**; 39, 975-980.
394. Giacobini, P., Parkash, J., Campagne, C., Messina, A., Casoni, F., Vanacker, C., Langlet, F., Hobo, B., Cagnoni, G., Gallet, S., *et al*. Brain endothelial cells control fertility through ovarian-steroid-dependent release of semaphorin 3A. *PLoS Biol*. **2014**; 12, e1001808.
395. Zhao, L., Chen, H., Lu, L., Zhao, C., Malichewe, C.V., Wang, L., Guo, X., Zhang, X. Design and screening of a novel neuropilin-1 targeted penetrating peptide for anti-angiogenic therapy in glioma. *Life Sci*. **2021**; 25, 119113.
396. Schulz, A., Gorodetska, I., Behrendt, R., Fuessel, S., Erdmann, K., Foerster, S., Datta, K., Mayr, T., Dubrovska, A., Muders, M.H. Linking NRP2 with EMT and chemoradioresistance in bladder cancer. *Front Oncol*. **2020**; 9, 1461.
397. Pellet-Many, C., Mehta, V., Fields, L., Mahmoud, M., Lowe, V., Evans, I., Ruivo, J., Zachary, I. Neuropilins 1 and 2 mediate neointimal hyperplasia and re-endothelialization following arterial injury. *Cardiovasc Res*. **2015**; 108, 288-298.
398. Heuckeroth, R.O., Enomoto, H., Grider, J.R., Golden, J.P., Hanke, J.A., Jackman, A., Molliver, D.C., Bardgett, M.E., Snider, W.D., Johnson, E.M. Jr., Milbrandt, J. Gene targeting reveals a critical role for neurturin in the development and maintenance of enteric, sensory, and parasympathetic neurons. *Neuron*. **1999**; 22, 253-263.
399. Wang, T., Jing, X., DeBerry, J.J., Schwartz, E.S., Molliver, D.C., Albers, K.M., Davis, B.M. Neurturin overexpression in skin enhances expression of TRPM8 in cutaneous sensory neurons and leads to behavioral sensitivity to cool and menthol. *J Neurosci*. **2013**; 33, 2060-2070.
400. Oglodek, E.A., Just, M.J., Szromek, A.R., Araszkiewicz, A. Melatonin and neurotrophins NT-3, BDNF, NGF in patients with varying levels of depression severity. *Pharmacol Rep*. **2016**; 68, 945-951.
401. Selim, A.O., Selim, S.A., Shalaby, S.M., Mosaad, H., Saber, T. Neuroprotective effects of placenta-derived mesenchymal stromal cells in a rat model of experimental autoimmune encephalomyelitis. *Cytotherapy*. **2016**; 18, 1100-1113.
402. Ginsberg, S.D., Malek-Ahmadi, M.H., Alldred, M.J., Che, S., Elarova, I., Chen, Y., Jeanneteau, F., Kranz, T.M., Chao, M.V., Counts, S.E., Mufson, E.J. Selective decline of neurotrophin and neurotrophin receptor genes within CA1 pyramidal neurons and hippocampus proper: Correlation with cognitive performance and neuropathology in mild cognitive impairment and Alzheimer's disease. *Hippocampus*. **2019**; 29, 422-439.
403. Fragoso, J., Carvalho Jurema Santos, G., Thomaz da Silva, H., Oliveira Nogueira, V., Loizon, E., Vidal, H., Costa-Silva, J.H., da Silva Aragao, R., Pirola, L., Leandro, C.G. Maternal physical activity-induced adaptive transcriptional response in brain and placenta of mothers and rat offspring. *J Dev Orig Health Dis*. **2020**; 11, 108-117.
404. Radzinsky, V., Orazov, M., Sharapova, O., Khamoshina, M. Pathogenesis of pelvic pain syndrome associated with endometriosis in patients resistant to surgical treatment. *Gynecol Endocrinol*. **2020**; 36, 12-15.
405. Wu, C.H., Ho, W.Y., Lee, Y.C., Lin, C.L., Hsieh, Y.L. EXPRESS: NGF-trkA signaling modulates the analgesic effects of prostatic acid phosphatase in resiniferatoxin-induced neuropathy. *Mol Pain*. **2016**; 12, 1744806916656846.
406. Dorfman, M.D., Garcia-Rudaz, C., Alderman, Z., Kerr, B., Lomniczi, A., Dissen, G.A., Castellano, J.M., Garcia-Galiano, D., Gaytan, F., Xu, B., *et al*. Loss of Ntrk2/Kiss1r signaling in oocytes causes premature ovarian failure. *Endocrinology*. **2014**; 155, 3098-3111.
407. Lepa, C., Hoppe, S., Stober, A., Skryabin, B.V., Sievers, L.K., Heitplatz, B., Ciarimboli, G., Neugebauer, U., Lindenmeyer, M.T., Cohen, C.D., *et al*. TrkC Is Essential for nephron function and trans-activates Igf1R signaling. *J Am Soc Nephrol*. **2021**; 32, 357-374.
408. Tang, B., Jia, H., Kast, R.J., Thomas, E.A. Epigenetic changes at gene promoters in response to immune activation in utero. *Brain Behav Immun*. **2013**; 30, 168-175.

409. Campbell, P., Morton, P.E., Takeichi, T., Salam, A., Roberts, N., Proudfoot, L.E., Mellerio, J.E., Aminu, K., Wellington, C., Patil, S.N., et al. Epithelial inflammation resulting from an inherited loss-of-function mutation in EGFR. *J Invest Dermatol*. **2014**; 134, 2570-2578.
410. McLaughlin, P.J., Keiper, C.L., Verderame, M.F., Zagon, I.S. Targeted overexpression of OGFr in epithelium of transgenic mice suppresses cell proliferation and impairs full-thickness wound closure. *Am J Physiol Regul Integr Comp Physiol*. **2012**; 302, R1084-R1090.
411. Filliol, D., Ghozland, S., Chluba, J., Martin, M., Matthes, H.W., Simonin, F., Befort, K., Gaveriaux-Ruff, C., Dierich, A., LeMeur, M., *et al*. Mice deficient for delta- and mu-opioid receptors exhibit opposing alterations of emotional responses. *Nat Genet*. **2000**; 25, 195-200.
412. Olabarrieta, E., Totorikaguena, L., Romero-Aguirregomezcorta, J., Agirregoitia, N., Agirregoitia, E. Delta and kappa opioid receptors on mouse sperm cells: expression, localization and involvement on in vitro fertilization. *Reprod Toxicol*. **2020**; 93, 211-218.
413. Riters, L.V., Cordes, M.A., Stevenson, S.A. Prodynorphin and kappa opioid receptor mRNA expression in the brain relates to social status and behavior in male European starlings. *Behav Brain Res*. **2017**; 320, 37-47.
414. Montagna, G., Gupta, H.V., Hannum, M., Tan, K.S., Lee, J., Scarpa, J.R., Plitas, G., Irie, T., McCormick, P.J., Fischer, G.W., *et al*. Intraoperative opioids are associated with improved recurrence-free survival in triple-negative breast cancer. *Br J Anaesth*. **2021**; 126, 367-376.
415. Andero, R., Brothers, S.P., Jovanovic, T., Chen, Y.T., Salah-Uddin, H., Cameron, M., Bannister, T.D., Almli, L., Stevens, J.S., Bradley, B., *et al*. Amygdala-dependent fear is regulated by Oprl1 in mice and humans with PTSD. *Sci Transl Med*. **2013**; 5, 188ra73.
416. Zhao, C., Quan, X., He, J., Zhao, R., Zhang, Y., Li, X., Sun, S., Ma, R., Zhang, Q. Identification of significant gene biomarkers of low back pain caused by changes in the osmotic pressure of nucleus pulposus cells. *Sci Rep*. **2020**; 10, 3708.
417. Sun, J., Chen, S.R., Pan, H.L. μ-Opioid receptors in primary sensory neurons are involved in supraspinal opioid analgesia. *Brain Res*. **2020**; 1729, 146623.
418. Olabarrieta, E., Totorikaguena, L., Romero-Aguirregomezcorta, J., Agirregoitia, N., Agirregoitia, E. Mu opioid receptor expression and localisation in murine spermatozoa and its role in IVF. *Reprod Fertil Dev*. **2020**; 32, 349-354.
419. Yanpeng, W., Qiongxiao, H., Sheng, X.U., Jing, S. Establishment of mouse endometrial injury model by curettage or coagulation. *Zhejiang Da Xue Xue Bao Yi Xue Ban (Journal of Zhejiang University: Medical sciences)*. **2017**; 46, 186-191.
420. Li, Y., Chen, F., Xie, Y., Yang, Q., Luo, H., Jia, P., Shi, Z., Wang, S., Zheng, X. Feiyangchangweiyan capsule protects against ulcerative colitis in mice by modulating the OSM/OSMR pathway and improving gut microbiota. *Phytomedicine*. **2021**; 80, 153372.
421. Dillon, S.R., Sprecher, C., Hammond, A., Bilsborough, J., Rosenfeld-Franklin, M., Presnell, S.R., Haugen, H.S., Maurer, M., Harder, B., Johnston, J., et al. Interleukin 31, a cytokine produced by activated T cells, induces dermatitis in mice. *Nat Immunol*. **2004**; 5, 752-760.
422. Caldwell, H.K., Aulino, E.A., Freeman, A.R., Miller, T.V., Witchey, S.K. Oxytocin and behavior: lessons from knockout mice. *Dev Neurobiol*. **2017**; 77, 190-201.
423. Naik, R.R., de Jong, T.R. Transient and persistent behavioral and molecular changes in primiparous female Wistar rats. *Eur J Neurosci*. **2017**; 45, 797-804.
424. Liao, C.H., Akazawa, H., Tamagawa, M., Ito, K., Yasuda, N., Kudo, Y., Yamamoto, R., Ozasa, Y., Fujimoto, M., Wang, P., *et al*. Cardiac mast cells cause atrial fibrillation through PDGF-A-mediated fibrosis in pressure-overloaded mouse hearts. *J Clin Invest*. **2010**; 120, 242-253.
425. Paule, S., Nebl, T., Webb, A.I., Vollenhoven, B., Rombauts, L.J., Nie, G. Proprotein convertase 5/6 cleaves platelet-derived growth factor A in the human endometrium in preparation for embryo implantation. *Mol Hum Reprod*. **2015**; 21, 262-270.
426. Bouet, P.E., Boueilh, T., de la Barca, J.M.C., Boucret, L., Blanchard, S., Ferre-L'Hotellier, V., Jeannin, P., Descamps, P., Procaccio, V., Reynier, P., May-Panloup, P. The cytokine profile of follicular fluid changes during ovarian ageing. *J Gynecol Obstet Hum Reprod*. **2020**; 49, 101704.
427. Niklasson, M., Bergstrom, T., Zhang, X.Q., Gustafsdottir, S.M., Sjogren, M., Edqvist, P.H., Vennstrom, B., Forsberg, M., Forsberg-Nilsson, K. Enlarged lateral ventricles and aberrant behavior in mice overexpressing PDGF-B in embryonic neural stem cells. *Exp Cell Res*. **2010**; 316, 2779-2789.
428. Ouyang, X., You, S., Zhang, Y., Zhang, C., Zhang, G., Shao, X., He, F., Hu, L. Transplantation of human amnion epithelial cells improves endometrial regeneration in rat model of intrauterine adhesions. *Stem Cells Dev*. **2020**; 29, 1346-1362.
429. Bruland, O., Fluge, O., Akslen, L.A., Eiken, H.G., Lillehaug, J.R., Varhaug, J.E., Knappskog, P.M. Inverse correlation between PDGFC expression and lymphocyte infiltration in human papillary thyroid carcinomas. *BMC Cancer*. **2009**; 9, 425.
430. Gladh, H., Folestad, E.B., Muhl, L., Ehnman, M., Tannenberg, P., Lawrence, A.L., Betsholtz, C., Eriksson, U. Mice lacking platelet-derived growth factor D display a mild vascular phenotype. *PLoS One*. **2016**; 11, e0152276.

431. Uutela, M., Wirzenius, M., Paavonen, K., Rajantie, I., He, Y., Karpanen, T., Lohela, M., Wiig, H., Salven, P., Pajusola, K., et al. PDGF-D induces macrophage recruitment, increased interstitial pressure, and blood vessel maturation during angiogenesis. *Blood*. **2004**; 104, 3198-3204.
432. Qian, C., Wong, C.W.Y., Wu, Z., He, Q., Xia, H., Tam, P.K.H., Wong, K.K.Y., Lui, V.C.H. Stage specific requirement of platelet-derived growth factor receptor-$\alpha$ in embryonic development. *PLoS One*. **2017**; 12, e0184473.
433. Foster, D.S., Marshall, C.D., Gulati, G.S., Chinta, M.S., Nguyen, A., Salhotra, A., Jones, R.E., Burcham, A., Lerbs, T., Cui, L., et al. Elucidating the fundamental fibrotic processes driving abdominal adhesion formation. *Nat Commun*. **2020**; 11, 4061.
434. Gao, Z., Sasaoka, T., Fujimori, T., Oya, T., Ishii, Y., Sabit, H., Kawaguchi, M., Kurotaki, Y., Naito, M., Wada, T., *et al*. Deletion of the PDGFR-beta gene affects key fibroblast functions important for wound healing. *J Biol Chem*. **2005**; 280, 9375-9389.
435. Yang, W., Yan, H., Wang, K., Cui, Y., Zhou, T., Xu, H., Zhu, H., Liu, J., Lan, X., Qu, L., *et al*. Goat PDGFRB: unique mRNA expression profile in gonad and significant association between genetic variation and litter size. *R Soc Open Sci*. **2019**; 6, 180805.
436. Li, L., Chen, X., Hao, L., Chen, Q., Liu, H., Zhou, Q. Exploration of immune-related genes in high and low tumor mutation burden groups of chromophobe renal cell carcinoma. *Biosci Rep*. **2020**; 40, bsr20201491.
437. Coan, P.M., Hummel, O., Garcia Diaz, A., Barrier, M., Alfazema, N., Norsworthy, P.J., Pravenec, M., Petretto, E., Hubner, N., Aitman, T.J. Genetic, physiological and comparative genomic studies of hypertension and insulin resistance in the spontaneously hypertensive rat. *Dis Model Mech*. **2017**; 10, 297-306.
438. Nakao, K., Iwata, K., Takeshita, T., Ozawa, H. Expression of hypothalamic kisspeptin, neurokinin B, and dynorphin A neurons attenuates in female Zucker fatty rats. *Neurosci Lett*. **2018**; 665, 135-139.
439. Szklarczyk, K., Korostynski, M., Golda, S., Solecki, W., Przewlocki, R. Genotype-dependent consequences of traumatic stress in four inbred mouse strains. *Genes Brain Behav*. **2012;** 11, 977-985.
440. Melo, I., Drews, E., Zimmer, A., Bilkei-Gorzo, A. Enkephalin knockout male mice are resistant to chronic mild stress. *Genes Brain Behav*. **2014**; 13, 550-558.
441. DeAtley, K.L., Colgrave, M.L., Canovas, A., Wijffels, G., Ashley, R.L., Silver, G.A., Rincon, G., Medrano, J.F., Islas-Trejo, A., Fortes, M.R.S., *et al*. Neuropeptidome of the hypothalamus and pituitary gland of indicine × taurine heifers: evidence of differential neuropeptide processing in the pituitary gland before and after puberty. *J Proteome Res*. **2018**; 17, 1852-1865.
442. Kay, V.R., Cahill, L.S., Hanif, A., Sled, J.G., Carmeliet, P., Tayade, C., Croy, B.A. Adult Pgf-/- mice behaviour and neuroanatomy are altered by neonatal treatment with recombinant placental growth factor. *Sci Rep*. **2019**; 9, 9285.
443. Soares de Carvalho, J.G., Tonizza de Carvalho, N.A., Souza, D.C., Martins Junior, B., Macedo, G.G., Vieira, L.M., Sales, J.N.S., Ghuman, S.S., D'Occhio, M.J., Baruselli, P.S. Administration of PGF2$\alpha$ during the periovulatory period increased fertilization rate in superovulated buffaloes. *Theriogenology*. **2020**; 145, 138-143.
444. Gebril, M., Hirota, Y., Aikawa, S., Fukui, Y., Kaku, T., Matsuo, M., Hirata, T., Akaeda, S., Hiraoka, T., Shimizu-Hirota, R., *et al*. Uterine epithelial progesterone receptor governs uterine receptivity through epithelial cell differentiation. *Endocrinology*. **2020**; 161, bqaa195.
445. Wu, J.R., Zhao, Y., Zhou, X.P., Qin, X. Estrogen receptor 1 and progesterone receptor are distinct biomarkers and prognostic factors in estrogen receptor-positive breast cancer: evidence from a bioinformatic analysis. *Biomed Pharmacother*. **2020**; 121, 109647.
446. Tian, Y., Stamova, B., Jickling, G.C., Xu, H., Liu, D., Ander, B.P., Bushnell, C., Zhan, X., Turner, R.J., Davis, R.R., *et al*. Y chromosome gene expression in the blood of male patients with ischemic stroke compared with male controls. *Gend Med*. **2012**; 9, 68-75.e3.
447. Mithani, S.K., Smith, I.M., Califano, J.A. Use of integrative epigenetic and cytogenetic analyses to identify novel tumor-suppressor genes in malignant melanoma. *Melanoma Res*. **2011**; 21, 298-307.
448. Kawashima, N., Fugate, J., Kusnecov, A.W. Immunological challenge modulates brain orphanin FQ/nociceptin and nociceptive behavior. *Brain Res*. **2002**; 949, 71-78.
449. Johnson, K.J., Robbins, A.K., Wang, Y., McCahan, S.M., Chacko, J.K., Barthold, J.S. Insulin-like 3 exposure of the fetal rat gubernaculum modulates expression of genes involved in neural pathways. *Biol Reprod*. **2010**; 83, 774-782.
450. Vaanholt, L.M., Turek, F.W., Meerlo, P. Beta-endorphin modulates the acute response to a social conflict in male mice but does not play a role in stress-induced changes in sleep. *Brain Res*. **2003**; 978, 169-176.
451. Andino, L.M., Ryder, D.J., Shapiro, A., Matheny, M.K., Zhang, Y., Judge, M.K., Cheng, K.Y., Tumer, N., Scarpace, P.J. POMC overexpression in the ventral tegmental area ameliorates dietary obesity. *J Endocrinol*. **2011**; 210, 199-207.

452. Gao, J., Zhang, Y., Zhang, T., Yang, Y., Yuan, C., Jia, J., Wang, Z. Responses of gonadal transcriptome and physiological analysis following exposure to 17α-ethynylestradiol in adult rare minnow Gobiocypris rarus. *Ecotoxicol Environ Saf*. **2017**; 141, 209-215.
453. van Kempen, L.C., Redpath, M., Elchebly, M., Klein, K.O., Papadakis, A.I., Wilmott, J.S., Scolyer, R.A., Edqvist, P.H., Ponten, F., Schadendorf, D., *et al*. The protein phosphatase 2A regulatory subunit PR70 is a gonosomal melanoma tumor suppressor gene. *Sci Transl Med*. **2016**; 8, 369ra177.
454. Dinarvand, P., Moser, K.A. Protein C deficiency. *Arch Pathol Lab Med*. **2019**; 143, 1281-1285.
455. Lay, A.J., Liang, Z., Rosen, E.D., Castellino, F.J. Mice with a severe deficiency in protein C display prothrombotic and proinflammatory phenotypes and compromised maternal reproductive capabilities. *J Clin Invest*. 2005; **115**, 1552-1561.
456. Tomac, A.C., Agulnick, A.D., Haughey, N., Chang, C.F., Zhang, Y., Backman, C., Morales, M., Mattson, M.P., Wang, Y., Westphal, H., Hoffer, B.J. Effects of cerebral ischemia in mice deficient in Persephin. *Proc Natl Acad Sci USA*. **2002**; 99, 9521-9526.
457. Ho, T.W., Bristol, L.A., Coccia, C., Li, Y., Milbrandt, J., Johnson, E., Jin, L., Bar-Peled, O., Griffin, J.W., Rothstein, JD. TGFbeta trophic factors differentially modulate motor axon outgrowth and protection from excitotoxicity. *Exp Neurol*. **2000**; 161, 664-675.
458. Lundin, C., Hjorth, L., Behrendtz, M., Nordgren, A., Palmqvist, L., Andersen, M.K., Biloglav, A., Forestier, E., Paulsson, K., Johansson, B. High frequency of BTG1 deletions in acute lymphoblastic leukemia in children with down syndrome. *Genes Chromosomes Cancer*. **2012**; 51, 196-206.
459. Fujiwara, S., Yamashita, Y., Choi, Y.L., Watanabe, H., Kurashina, K., Soda, M., Enomoto, M., Hatanaka, H., Takada, S., Ozawa, K., Mano, H. Transforming activity of purinergic receptor P2Y, G protein coupled, 8 revealed by retroviral expression screening. *Leuk Lymphoma*. **2007**; 48, 978-986.
460. Viemari, J.C., Maussion, G., Bevengut, M., Burnet, H., Pequignot, J.M., Nepote, V., Pachnis, V., Simonneau, M., Hilaire, G. Ret deficiency in mice impairs the development of A5 and A6 neurons and the functional maturation of the respiratory rhythm. *Eur J Neurosci*. **2005**; 22, 2403-2412.
461. Sinha, D., Kalimutho, M., Bowles, J., Chan, A.L., Merriner, D.J., Bain, A.L., Simmons, J.L., Freire, R., Lopez, J.A., Hobbs, R.M., *et al*. Cep55 overexpression causes male-specific sterility in mice by suppressing Foxo1 nuclear retention through sustained activation of PI3K/Akt signaling. *FASEB J*. **2018**; 32, 4984-4999.
462. Ahmadi Rastegar, D., Sharifi Tabar, M., Alikhani, M., Parsamatin, P., Sahraneshin Samani, F., Sabbaghian, M., Sadighi Gilani, M.A., Mohammad Ahadi, A., Mohseni Meybodi, A., Piryaei, A., *et al*. Isoform-level gene expression profiles of human Y chromosome azoospermia factor genes and their X chromosome paralogs in the testicular tissue of non-obstructive azoospermia patients. *J Proteome Res*. **2015**; 14, 3595-3605.
463. Yang, H., Li, Q., Zhang, L., Zhu, M., Niu, J., Xue, F., Yang, L., Qu, Q., Lao, Y., Ding, Z., *et al*. LncPRYP4-3 serves as a novel diagnostic biomarker for dissecting subtypes of metabolic associated fatty liver disease by targeting RPS4Y2. *Clin Exp Med*. **2020**; 20, 587-600.
464. Papageorghiou, A., Mansour, S. SHOX haploinsufficiency presenting with isolated short long bones in the second and third trimester. *Eur J Hum Genet*. **2018**; 26, 350-358.
465. Brosens, E., de Jong, E.M., Barakat, T.S., Eussen, B.H., D'haene, B., De Baere, E., Verdin, H., Poddighe, P.J., Galjaard, R.J., Gribnau, J., *et al*. Structural and numerical changes of chromosome X in patients with esophageal atresia. *Eur J Hum Genet*. **2014**; 22, 1077-1084.
466. Young, J.W., van Enkhuizen, J., Winstanley, C.A., Geyer, M.A. Increased risk-taking behavior in dopamine transporter knockdown mice: further support for a mouse model of mania. *J Psychopharmacol*. **2011**; 25, 934-943.
467. Feistauer, V., Fisch, J., da Silva Oliveira, C.K., Giovenardi, M., Almeida, S. Restriction and hyperlipidic diets during pregnancy, lactation and adult life modified the expression of dopaminergic system related genes both in female mice and their adult offspring. *Brain Res Bull*. **2020**; 162, 245-252.
468. Greig, C.J., Zhang, L., Cowles, R.A. Potentiated serotonin signaling in serotonin re-uptake transporter knockout mice increases enterocyte mass and small intestinal absorptive function. *Physiol Rep*. **2019**; 7, e14278.
469. Morgan, J.A., Singhal, G., Corrigan, F., Jaehne, E.J., Jawahar, M.C., Breen, J., Pederson, S., Baune, B.T. Ceasing exercise induces depression-like, anxiety-like, and impaired cognitive-like behaviours and altered hippocampal gene expression. *Brain Res Bull*. **2019**; 148, 118-130.
470. Clemencon, B., Babot, M., Trezeguet, V. The mitochondrial ADP/ATP carrier (SLC25 family): pathological implications of its dysfunction. *Mol Aspects Med*. **2013**; 34, 485-493.
471. Guo, X., Huang, Y., Qi, Y., Liu, Z., Ma, Y., Shao, Y., Jiang, S., Sun, Z., Ruan, Q. Human cytomegalovirus miR-UL36-5p inhibits apoptosis via downregulation of adenine nucleotide translocator 3 in cultured cells. *Arch Virol*. **2015**; 160, 2483-2490.
472. Abeliovich, A., Schmitz, Y., Farinas, I., Choi-Lundberg, D., Ho, W.H., Castillo, P.E., Shinsky, N., Verdugo, J.M., Armanini, M., Ryan, A., *et al*. Mice lacking alpha-synuclein display functional deficits in the nigrostriatal dopamine system. *Neuron*. **2000**; 25, 239-252.
473. Vartiainen, S., Aarnio, V., Lakso, M., Wong, G. Increased lifespan in transgenic Caenorhabditis elegans overexpressing human alpha-synuclein. *Exp Gerontol*. **2006**; 41, 871-876.

474. Garratt, M., Bathgate, R., de Graaf, S.P., Brooks, R.C. Copper-zinc superoxide dismutase deficiency impairs sperm motility and in vivo fertility. *Reproduction*. **2013**; 146, 297-304.
475. Herman, S., Lipinski, P., Ogorek, M., Starzynski, R., Grzmil, P., Bednarz, A., Lenartowicz, M. Molecular regulation of copper homeostasis in the male gonad during the process of spermatogenesis. *Int J Mol Sci*. **2020**; 21, E9053.
476. Wang, J., Jiang, Q., Faleti, O.D., Tsang, C.M., Zhao, M., Wu, G., Tsao, S.W., Fu, M., Chen, Y., Ding, T., *et al*. Exosomal delivery of AntagomiRs targeting viral and cellular microRNAs synergistically inhibits cancer angiogenesis. *Mol Ther Nucleic Acids*. **2020**; 22, 153-165.
477. Ning, Z., Williams, J.M., Kumari, R., Baranov, P.V., Moore, T. Opposite expression patterns of spry3 and p75NTR in cerebellar vermis suggest a male-specific mechanism of autism pathogenesis. *Front Psychiatry*. **2019**; 10, 416.
478. Caron, K.M., Soo, S.C., Wetsel, W.C., Stocco, D.M., Clark, B.J., Parker, K.L. Targeted disruption of the mouse gene encoding steroidogenic acute regulatory protein provides insights into congenital lipoid adrenal hyperplasia. *Proc Natl Acad Sci USA*. **1997**; 94, 11540-11545.
479. Horvath, A., Mathyakina, L., Vong, Q., Baxendale, V., Pang, A.L., Chan, W.Y., Stratakis, C.A. Serial analysis of gene expression in adrenocortical hyperplasia caused by a germline PRKAR1A mutation. *J Clin Endocrinol Metab*. **2006**; 91, 584-596.
480. Leon, S., Fergani, C., Talbi, R., Maguire, C.A., Gerutshang, A., Seminara, S.B., Navarro, V.M. Tachykinin signaling is required for the induction of the preovulatory LH surge and normal LH pulses. *Neuroendocrinology*. **2020**, doi: 10.1159/000509222.
481. Bludau, S., Muhleisen, T.W., Eickhoff, S.B., Hawrylycz, M.J., Cichon, S., Amunts, K. Integration of transcriptomic and cytoarchitectonic data implicates a role for MAOA and TAC1 in the limbic-cortical network. *Brain Struct Funct*. **2018**; 223, 2335-2342.
482. Yang, J.J., Caligioni, C.S., Chan, Y.M., Seminara, S.B. Uncovering novel reproductive defects in neurokinin B receptor null mice: closing the gap between mice and men. *Endocrinology*. **2012**; 153, 1498-1508.
483. Voigt, C., Bennett, N. Reproductive status-dependent dynorphin and neurokinin B gene expression in female Damaraland mole-rats. *J Chem Neuroanat*. **2019**; 102, 101705.
484. Naono-Nakayama, R., Sunakawa, N., Ikeda, T., Matsushima, O., Nishimori, T. Subcutaneous injection of endokinin C/D attenuates carrageenan-induced inflammation. *Peptides*. **2010**; 31, 1767-1771.
485. Aczel, T., Kecskes, A., Kun, J., Szenthe, K., Banati, F., Szathmary, S., Herczeg, R., Urban, P., Gyenesei, A., Gaszner, B., *et al*. Hemokinin-1 gene expression is upregulated in trigeminal ganglia in an inflammatory orofacial pain model: potential role in peripheral sensitization. *Int J Mol Sci*. **2020**; 21, 2938.
486. Ceci, L., Francis, H., Zhou, T., Giang, T., Yang, Z., Meng, F., Wu, N., Kennedy, L., Kyritsi, K., Meadows, V., et al. Knockout of the tachykinin receptor 1 in the Mdr2-/- (Abcb4-/-) mouse model of primary sclerosing cholangitis reduces biliary damage and liver fibrosis. *Am J Pathol*. **2020**; 190, 2251-2266.
487. Simavli, S., Thompson, I.R., Maguire, C.A., Gill, J.C., Carroll, R.S., Wolfe, A., Kaiser, U.B., Navarro, V.M. Substance p regulates puberty onset and fertility in the female mouse. *Endocrinology*. **2015**; 156, 2313-2322.
488. Torres, E., Velasco, I., Franssen, D., Heras, V., Gaytan, F., Leon, S., Navarro, V., Pineda, R., Candenas, M.L., Romero-Ruiz, A., Tena-Sempere, M. Congenital ablation of Tacr2 reveals overlapping and redundant roles of NK2R signaling in the control of reproductive axis. *Am J Physiol Endocrinol Metab*. **2021,** doi: 10.1152/ajpendo.00346.2020.
489. Bardelli, C., Amoruso, A., Manzetti, E., Fresu, L.G., Valsesia, R., Zeppegno, P., Brunelleschi, S. Recurrent major depressive disorder: Imbalance of neurokinin (NK)-1 and NK-2 receptor expression in monocytes. *Pharmacol Res*. **2013**; 68, 24-30.
490. Meyfour, A., Ansari, H., Pahlavan, S., Mirshahvaladi, S., Rezaei-Tavirani, M., Gourabi, H., Baharvand, H., Salekdeh, G.H. Y chromosome missing protein, TBL1Y, may play an important role in cardiac differentiation. *J Proteome Res*. **2017**; 16, 4391-4402.
491. Moraveji, S.F., Esfandiari, F., Taleahmad, S., Nikeghbalian, S., Sayahpour, F.A., Masoudi, N.S., Shahverdi, A., Baharvand, H. Suppression of transforming growth factor-beta signaling enhances spermatogonial proliferation and spermatogenesis recovery following chemotherapy. *Hum Reprod*. **2019**; 34, 2430-2442.
492. Li, H., Li, W., Liang, B., Wei, J., Yin, D., Fan, Q. Role of AP-2α/TGF-β1/Smad3 axis in rats with intervertebral disc degeneration. *Life Sci*. **2020**; 263, 118567.
493. Sanford, L.P., Ormsby, I., Gittenberger-de Groot, A.C., Sariola, H., Friedman, R., Boivin, G.P., Cardell, E.L., Doetschman, T. TGFbeta2 knockout mice have multiple developmental defects that are non-overlapping with other TGFbeta knockout phenotypes. *Development*. 1997; 124, 2659-2670.
494. Kim, J.H., Lee, Y.W., Park, Y.M., Park, K.A., Park, S.H., Lee, W.T., Lee, J.E. Agmatine-reduced collagen scar area accompanied with surface righting reflex recovery after complete transection spinal cord injury. *Spine (Phila Pa 1976)*. **2011**; 36, 2130-2138.
495. Nilsson, L.L., Hornstrup, M.B., Perin, T.L., Lindhard, A., Funck, T., Bjerrum, P.J., Mule, H.T., Scheike, T., Nielsen, H.S., Hviid, T.V.F. Soluble HLA-G and TGF-β in couples attending assisted reproduction - a possible role of TGF-β isoforms in semen? *J Reprod Immunol*. **2020**; 137, 102857.

496. Ciebiera, M., Wlodarczyk, M., Wrzosek, M., Męczekalski, B., Nowicka, G., Lukaszuk, K., Ciebiera, M., Słabuszewska-Jozwiak ,A., Jakiel, G. Role of transforming growth factor β in uterine fibroid biology. *Int J Mol Sci*. **2017**; 18, 2435.
497. Wang, B.J., Chi, K.P., Shen RL, Zheng SW, Guo Y, Li JF, Fei J, He Y. TGFBI Promotes tumor growth and is associated with poor prognosis in oral squamous cell carcinoma. *J Cancer*. **2019**; 10, 4902-4912.
498. Yang, P., Schmit, B.M., Fu, C., DeSart, K., Oh, S.P., Berceli, S.A., Jiang, Z. Smooth muscle cell-specific Tgfbr1 deficiency promotes aortic aneurysm formation by stimulating multiple signaling events. *Sci Rep*. **2016**; 6, 35444.
499. Agis, H., Collins, A., Taut, A.D., Jin, Q., Kruger, L., Gorlach, C., Giannobile, W.V. Cell population kinetics of collagen scaffolds in ex vivo oral wound repair. *PLoS One*. **2014**; 9, e112680.
500. Landrum, M.J., Lee, J.M., Riley, G.R., Jang, W., Rubinstein, W.S., Church, D.M., Maglott, D.R. ClinVar: public archive of relationships among sequence variation and human phenotype. *Nucleic Acids Res*. **2014**; 42, D980-D985.
501. Li, W., Lu, H., Wang, H., Ning, X., Liu, Q., Zhang, H., Liu, Z., Wang, J., Zhao, W., Gu, Y., *et al*. Circular RNA TGFBR2 acts as a ceRNA to suppress nasopharyngeal carcinoma progression by sponging miR-107. *Cancer Lett*. **2021**; 499, 301-313.
502. Yamano, S., Kuo, W.P., Sukotjo, C. Downregulated gene expression of TGF-βs in diabetic oral wound healing. *J Craniomaxillofac Surg*. **2013**; 41, e42-e48.
504. Wehrhan, F., Grabenbauer, G.G., Rodel, F., Amann, K., Schultze-Mosgau, S. Exogenous modulation of TGF-beta(1) influences TGF-betaR-III-associated vascularization during wound healing in irradiated tissue. *Strahlenther Onkol*. **2004**; 180, 526-533.
505. Stopa, L.R.S., de Souza, C.F., Martins, A.B., Lopes, G.M., Costa, N.O., Gerardin, D.C.C., de Carvalho, G.G., Zaia, D.A.M., Zaia, C.T.B.V., Uchoa, E.T., Leite, C.M. Neonatal overfeeding reduces estradiol plasma levels and disrupts noradrenergic-kisspeptin-GnRH pathway and fertility in adult female rats. *Mol Cell Endocrinol*. **2020**; 524, 111147.
506. Wang, Y.L., Su, Y.S., He, W., Jing, X.H. Electroacupuncture relieved visceral and referred hindpaw hypersensitivity in colitis rats by inhibiting tyrosine hydroxylase expression in the sixth lumbar dorsal root ganglia. *Neuropeptides*. **2019**; 77, 101957.
507. Sood, R., Sholl, L., Isermann, B., Zogg, M., Coughlin, S.R., Weiler, H. Maternal Par4 and platelets contribute to defective placenta formation in mouse embryos lacking thrombomodulin. *Blood*. **2008**; 112, 585-591.
508. Asai, Y., Yamamoto, T., Kito, D., Ichikawa, K., Abe, Y. Factors influencing the effectiveness of recombinant human soluble thrombomodulin on disseminated intravascular coagulation: a retrospective study. *J Pharm Health Care Sci*. **2020**; 6, 26.
509. Dasari, V.K., Goharderakhshan, R.Z., Perinchery, G., Li, L.C., Tanaka, Y., Alonzo, J., Dahiya, R. Expression analysis of Y chromosome genes in human prostate cancer. *J Urol*. **2001**; 165, 1335-1341.
510. Wong, H.Y., Wang, G.M., Croessmann, S., Zabransky, D.J., Chu, D., Garay, J.P., Cidado, J., Cochran, R.L., Beaver, J.A., Aggarwal, A., *et al*. TMSB4Y is a candidate tumor suppressor on the Y chromosome and is deleted in male breast cancer. *Oncotarget*. **2015**; 6, 44927-44940.
511. Alenina, N., Kikic, D., Todiras, M., Mosienko, V., Qadri, F., Plehm, R., Boye, P., Vilianovitch, L., Sohr, R., Tenner, K., *et al*. Growth retardation and altered autonomic control in mice lacking brain serotonin. *Proc Natl Acad Sci USA*. **2009**; 106, 10332-10337.
512. Audero, E., Mlinar, B., Baccini, G., Skachokova, Z.K., Corradetti, R., Gross, C. Suppression of serotonin neuron firing increases aggression in mice. *J Neurosci*. **2013**; 33, 8678-8688.
513. Ijiri, T.W., Vadnais, M.L., Lin, A.M., Huang, A.P., Cao, W., Merdiushev, T., Gerton, G.L. Male mice express spermatogenic cell-specific triosephosphate isomerase isozymes. *Mol Reprod Dev*. **2013**; 80, 862-870.
514. Yu, W.L., Yu, G., Dong, H., Chen, K., Xie, J., Yu, H., Ji, Y., Yang, G.S., Li, A.J., Cong, W.M, Jin, G.Z. Proteomics analysis identified TPI1 as a novel biomarker for predicting recurrence of intrahepatic cholangiocarcinoma. *J Gastroenterol*. **2020**; 55, 1171-1182.
515. Halder, A., Kumar, P., Jain, M., Iyer, V.K. Copy number variations in testicular maturation arrest. *Andrology*. **2017**; 5, 460-472.
516. Gegenschatz-Schmid, K., Verkauskas, G., Stadler, M.B., Hadziselimovic, F. Genes located in Y-chromosomal regions important for male fertility show altered transcript levels in cryptorchidism and respond to curative hormone treatment. *Basic Clin Androl*. **2019**; 29, 8.
517. Zhang, X.F., Zhang, T., Wang, L., Zhang, H.Y., Chen, Y.D., Qin, X.S., Feng, Y.M., Feng, Y.N., Shen, W., Li, L. Effects of diethylhexyl phthalate (DEHP) given neonatally on spermatogenesis of mice. *Mol Biol Rep*. **2013**; 40, 6509-6517.
518. Heidecker, B., Lamirault, G., Kasper, E.K., Wittstein, I.S., Champion, H.C., Breton, E., Russell, S.D., Hall, J., Kittleson, M.M., Baughman, K.L., Hare, J.M. The gene expression profile of patients with new-onset heart failure reveals important gender-specific differences. *Eur Heart J*. **2010**; 31, 1188-1196.

519. Shpargel, K.B., Sengoku, T., Yokoyama, S., Magnuson, T. UTX and UTY demonstrate histone demethylase-independent function in mouse embryonic development. *PLoS Genet*. **2012**; 8, e1002964.
520. Pottmeier, P., Doszyn, O., Peuckert, C., Jazin, E. Increased expression of Y-encoded demethylases during differentiation of human male neural stem cells. *Stem Cells Dev*. **2020**; 29, 1497-1509.
521. Zhu, L., Dong, L., Feng, M., Yang, F., Jiang, W., Huang, Z., Liu, F., Wang, L., Wang, G., Li, Q. Profiles of autophagy-related genes in esophageal adenocarcinoma. *BMC Cancer*. **2020**; 20, 943.
522. Tannour-Louet, M., Han, S., Louet, J.F., Zhang, B., Romero, K., Addai, J., Sahin, A., Cheung, S.W., Lamb, D.J. Increased gene copy number of VAMP7 disrupts human male urogenital development through altered estrogen action. *Nat Med*. **2014**; 20, 715-724.
523. Olesen, H., Pors, S.E., Jensen, L.B., Gronning, A.P., Lemser, C.E., Nguyen Heimburger, M.T.H., Mamsen, L.S., Getreu, N., Christensen, S.T., Andersen, C.Y., Kristensen, S.G. N-acetylcysteine protects ovarian follicles from ischemia-reperfusion injury in xenotransplanted human ovarian tissue. *Hum Reprod*. **2021**; 36, 429-443.
524. Wang, C., Wang, Q., Gao, W., Zhang, Z., Lou, Y., Jin, H., Chen, X., Lei, B., Xu, H., Mao, C. Highly efficient local delivery of endothelial progenitor cells significantly potentiates angiogenesis and full-thickness wound healing. *Acta Biomater*. **2018**; 69, 156-169.
525. Bellomo, D., Headrick, J.P., Silins, G.U., Paterson, C.A., Thomas, P.S., Gartside, M., Mould, A., Cahill, M.M., Tonks, I.D., Grimmond, S.M., *et al*. Mice lacking the vascular endothelial growth factor-B gene (Vegfb) have smaller hearts, dysfunctional coronary vasculature, and impaired recovery from cardiac ischemia. *Circ Res*. **2000**; 86, E29-E35.
526. Sun, Y., Jin, K., Childs, J.T., Xie, L., Mao, X.O., Greenberg, D.A. Vascular endothelial growth factor-B (VEGFB) stimulates neurogenesis: evidence from knockout mice and growth factor administration. *Dev Biol*. **2006**; 289, 329-335.
527. Radko, S., Koleva, M., James, K.M., Jung, R., Mymryk, J.S., Pelka, P. Adenovirus E1A targets the DREF nuclear factor to regulate virus gene expression, DNA replication, and growth. *J Virol*. **2014**; 88, 13469-13481.
528. Xi, J.F., Wang, X.Z., Zhang, Y.S., Jia, B., Li, C.C., Wang, X.H., Ying, R.W. Sex control by Zfy siRNA in the dairy cattle. *Anim Reprod Sci*. **2019**; 200, 1-6.
529. Jan, S.Z., Jongejan, A., Korver, C.M., van Daalen, S.K.M., van Pelt, A.M.M., Repping, S., Hamer, G. Distinct prophase arrest mechanisms in human male meiosis. *Development*. **2018**; 145, dev160614.
530. Slaets, H., Nelissen, S., Janssens, K., Vidal, P.M., Lemmens, E., Stinissen, P., Hendrix, S., Hellings, N. Oncostatin M reduces lesion size and promotes functional recovery and neurite outgrowth after spinal cord injury. *Mol Neurobiol*. **2014**; 50, 1142-1151.

© 2021 by the authors. Submitted for possible open access publication under the terms and conditions of the Creative Commons Attribution (CC BY) license (http://creativecommons.org/licenses/by/4.0/).
